# Discovery of selective, antimetastatic and anti-cancer stem cell metallohelices *via* post-assembly modification†
†Electronic supplementary information (ESI) available: Data created during this study are openly available from the University of Warwick Research Archive Portal (WRAP) (http://wrap.warwick.ac.uk). See DOI: 10.1039/c9sc02651g


**DOI:** 10.1039/c9sc02651g

**Published:** 2019-07-18

**Authors:** Hualong Song, Nicola J. Rogers, Simon J. Allison, Viktor Brabec, Hannah Bridgewater, Hana Kostrhunova, Lenka Markova, Roger M. Phillips, Emma C. Pinder, Samantha L. Shepherd, Lawrence S. Young, Juraj Zajac, Peter Scott

**Affiliations:** a Department of Chemistry , University of Warwick , Coventry CV4 7AL , UK . Email: peter.scott@warwick.ac.uk; b School of Applied Sciences , University of Huddersfield , Huddersfield , HD1 3DH , UK; c The Czech Academy of Sciences , Institute of Biophysics , Kralovopolska 135 , CZ-61265 Brno , Czech Republic; d Warwick Medical School , University of Warwick , Coventry CV4 7AL , UK

## Abstract

Helicates and related metallofoldamers, synthesised by dynamic self-assembly, represent an area of chemical space inaccessible by traditional organic synthesis, and yet with potential for discovery of new classes of drug. Here we report that water-soluble, optically pure Fe(ii)- and even Zn(ii)-based triplex metallohelices are an excellent platform for post-assembly click reactions. By these means, the *in vitro* anticancer activity and most importantly the selectivity of a triplex metallohelix Fe(ii) system are dramatically improved. For one compound, a remarkable array of mechanistic and pharmacological behaviours is discovered: inhibition of Na^+^/K^+^ ATPase with potency comparable to the drug ouabain, antimetastatic properties (including inhibition of cell migration, re-adhesion and invasion), cancer stem cell targeting, and finally colonosphere inhibition competitive with the drug salinomycin.

## Abstract 2

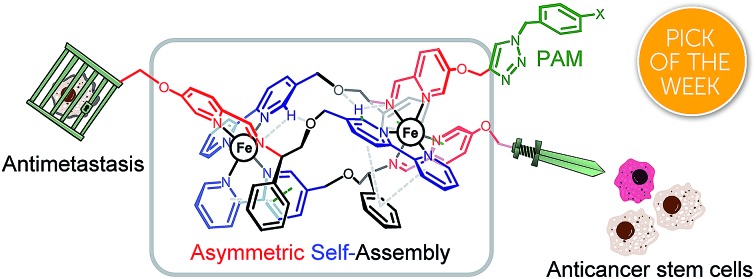
A remarkable array of mechanistic and pharmacological behaviours is discovered *via* click derivatisation of asymmetric, optically pure helicate-like compounds.

## Introduction

Lehn envisaged in the original report^1^ that helicates^2^–^4^ – self-assembling multimetallic coordination compounds – may find uses in biochemistry. Indeed, while their underlying chemistry is very different to that of the small cationic α-helical peptide units that are deployed in nature for *e.g.* signalling, structural and host-defence roles,^5^–^7^ some such metallofoldamers^8^ have similar dimensions and charge. With this in mind we have developed several classes of water-compatible, optically pure metallohelix compounds,^9^–^11^ each of which has unique properties, including a growing list of peptide-like behaviours: binding of DNA motifs,^12^ anticancer activity,^5^,^11^,^13^ and the inhibition of *e.g.* amyloid-β aggregation,^12^,^14^ enzyme activity^15^,^16^ and ice recrystallization.^17^ Thus, while we cannot expressly mimic the exquisite architectures of natural peptides, we are motivated to seek methods by which diverse metallohelices might be rapidly accessed and new biological properties discovered and optimised.^18^

The addition of new covalent bonds to supramolecular assemblies by post-assembly modification (PAM) may be used for various purposes including locking-down dynamic structures, triggering structural transformations, or simply late-stage derivatisation to introduce new functional groups.^19^ The latter is attractive to us since it may facilitate phenotypic discovery, property optimisation or the elucidation of structure/activity relationships, all without the need for extensive pre-assembly ligand synthesis. Further, we may include functional groups that are incompatible with the self-assembly. However, such reactions must be clean and efficient under mild conditions, and we note that the lability or reactivity of many metallosupramolecular structures means that application of the otherwise extremely versatile copper-catalysed azide/alkyne cycloaddition (CuAAC) may be hampered by deleterious reactions^20^ such as displacement of the original metal by copper from the catalyst.^21^ In addition the new triazole units formed may bind to metals.^22^

We report here that our self-assembled optically pure, water-soluble triplex metallohelices^8^ provide excellent molecular frameworks on which to perform such late-stage modification. Through this chemistry we have discovered compounds with excellent potency against a panel of cancer cell lines, with enantioselectivity reflected in cell cycle studies, plus enhanced selectivity with respect to a panel of non-cancer cell lines *in vitro*. One selected compound displays a remarkable array of properties: antimetastatic (inhibition of cell migration, re-adhesion and invasion), cancer stem cell targeting, and colonosphere inhibition competitive with the drug salinomycin. Mechanistically, the compound does not induce apoptosis but appears to inhibit Na^+^/K^+^ ATPase activity with potency comparable to the drug ouabain.

## Results

### Click synthesis of new metallohelices

**Fig. 1:**
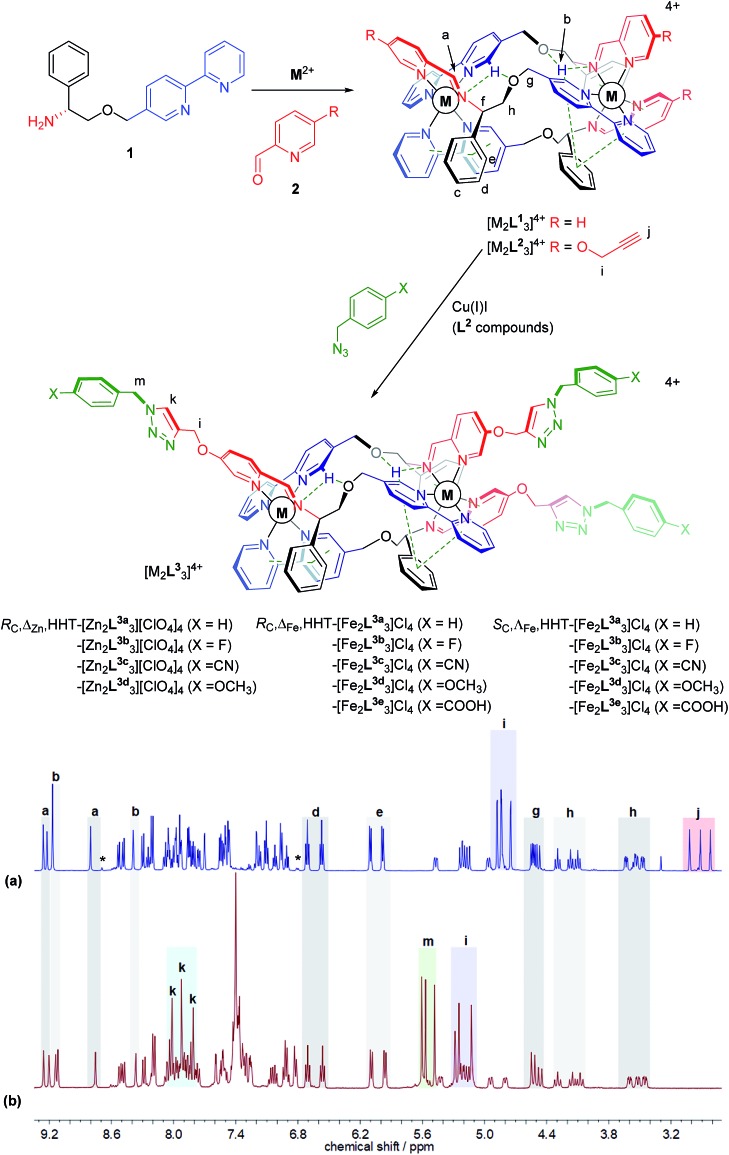
Metallohelix architectures *via* asymmetric self-assembly and CuAAC. Synthesis of the new alkyne-appended triplex enantiomers [M_2_**L^2^**_3_]^4+^ gives access to a range of optically pure structures of Zn(ii) and Fe(ii); the stereochemical descriptors for the complexes *e.g. R*_C_,ΔM,HHT refer to the absolute configurations at C, the metal, and the directionality of the ligand strands respectively. ^1^H NMR spectra (500 MHz, CD_3_CN, 298 K) show clean conversion of (a) *R*_C_-Δ_Zn_-HHT-[Zn_2_**L^2^**_3_][ClO_4_]_4_ to (b) *R*_C_-Δ_Zn_-HHT-[Zn_2_**L^3a^**_3_][ClO_4_]_4_. Alkyne resonances **j** are replaced by triazole singlets **k** but otherwise the triplex architecture is unperturbed; *indicates resonances assigned to the HHH isomer.

We recently synthesised ranges of optically pure water-soluble metallohelices in which the ligand strands run in opposing directions (the “head-to-head-to-tail” or HHT isomers).^9^ Of these we selected the system based on [M_2_**L^1^**_3_]^4+^ (Fig. 1, R = H) since it possesses an appealing facially amphipathic architecture. The new metallohelix enantiomers [M_2_**L^2^**_3_]^4+^ (M = Zn, Fe) with perchlorate and chloride counter-ions respectively, synthesised using sub-components **1** and 5-(propargyloxy)picolinaldehyde (**2**), are decorated with three chemically inequivalent alkyne substituents on one face of the structure.

The ^1^H NMR spectra have several unusual features that confirm the topologically asymmetric structure [Fig. 1a]. In [Zn_2_**L^2^**_3_][ClO_4_]_4_ at 293 K, three spectroscopically unique ligand environments give rise to imine singlets H^a^ at 9.26, 9.17 and 8.80 ppm. Two of the bpy protons H^b^ appear in the same region (9.22 and 9.17 ppm) as a result of intramolecular hydrogen bonds – see dotted lines in structures of Fig. 1. The third bpy proton H^b^ with no such interaction was found at 8.39 ppm. Similarly, the two sets of ring protons H^d^ and H^e^ arising from pendant phenyls taking part in bifurcated π-stacks with coordinated bpy units, appear at 6.80 and 5.90 ppm. The third phenyl group that is instead π-stacked to a coordinated pyridine has H^d^ and H^e^ resonances at more conventional chemical shifts of 7.11 and 6.96 ppm. At lower temperatures these signals begin to broaden, consistent with slowing of phenyl group de-coordination/rotation (ESI Fig. S3†). The rather rigid arrangement of the ligand strands leads to six distinct resonances for H^h^, clustered at 4.42–4.10 ppm (apparent triplets) and 3.63–3.47 ppm (approximately doublets of doublets). Three alkyne proton singlets H^j^ appear at 3.0–2.8 ppm.

In the same ^1^H NMR spectrum of Fig. 1 the small singlet at 8.7 ppm is assigned to the HHH isomer of this compound – the three-fold symmetric compound where all three strands run in the same direction – and on this assumption we estimate the selectivity HHT:HHH to be *ca.* 99%. Other small peaks consistent with the presence of this minor isomer can be seen in the baseline. At 6.8 ppm a doublet is tentatively assigned to protons of type e in the HHH isomer. It is interesting to note the absence of a triplet for type d protons in the region 6.4–6.8 ppm in this minor component; no such signal is expected since there is no phenyl-bpy π-stack in the HHH isomer. Similarly, no minor doublets for type e protons are expected around 6 ppm. As such, the appearance of these minor isomer peaks corroborates our assignments for the major isomer.

Reactions employing a range of CuAAC conditions^23^–^25^ were explored and it was found that heating [M_2_**L^2^**_3_]^4+^ (M = Zn, Fe) with benzyl azide in the presence of catalytic copper(i) iodide for 18 h cleanly gave [M_2_**L^3a^**_3_]^4+^. The disappearance of the alkyne resonances in the ^1^H NMR spectra of [Zn_2_**L^2^**_3_]^4+^ at *ca.* 3 ppm (H^j^) [Fig. 1b] and 78/77 ppm (C^j^/C^n^) (ESI Fig. S4†) demonstrates that the reaction progresses to completion. Upon formation of the triazole moiety, three new proton singlets (H^k^) are evident at *ca.* 8 ppm and new quaternary carbon resonances (C^l^) at *ca.* 142 ppm are observed. In addition, three new singlets at *ca.* 5.6 ppm are observed in the ^1^H NMR spectra of both [Zn_2_**L^2^**_3_]^4+^and [Fe_2_**L^2^**_3_]^4+^ (and ^13^C at *ca.* 55 ppm), due to the addition of the benzyl methylene group (H^m^), as well as new resonances due to the benzyl ring in the aromatic region. The NMR signals corresponding to the imine, bipyridyl and phenyl units remain unperturbed by the CuAAC reaction, demonstrating that the structure of the metallohelix architecture is preserved. The successful synthesis of all the “clicked” complexes was also confirmed by high resolution electrospray mass spectrometry (ESI Fig. S18–23†). For instance, a strong signal was observed by electrospray mass spectrometry at *m*/*z* 464.1580 Da for the tetracationic molecular ion of *R*_C_,Δ_Fe_,HHT-[Fe_2_**L^3a^**_3_]Cl_4_, within 0.001 Da of the calculated value for C_105_H_93_Fe_2_N_21_O_6_ (*m*/*z* 464.1582 Da). Inductively-Coupled Plasma Mass Spectrometry (ICP-MS) analysis of the iron triplex metallohelices revealed that only trace amounts of copper could be detected.

Subsequently fourteen new benzyl triazole-functionalised metallohelix enantiomers were isolated in a similar manner. Remarkably, the carboxylic acid derivatives [Fe_2_**L^3e^**_3_]^4+^ were accessible, despite the stability of Fe carboxylates. In all cases, the triplex architecture was retained, even in the case of conventionally highly labile Zn(ii). Characterizing data including NMR, MS, circular dichroism (CD), IR and microanalysis are detailed in ESI.† NMR and UV-vis experiments indicate that little decomposition of the product [Fe_2_**L^3a^**_3_]Cl_4_ occurred over months in aqueous solution (ESI Fig. S14, S15 and S17†).

### Antiproliferative activity and cell line selectivity studies

**Fig. 2:**
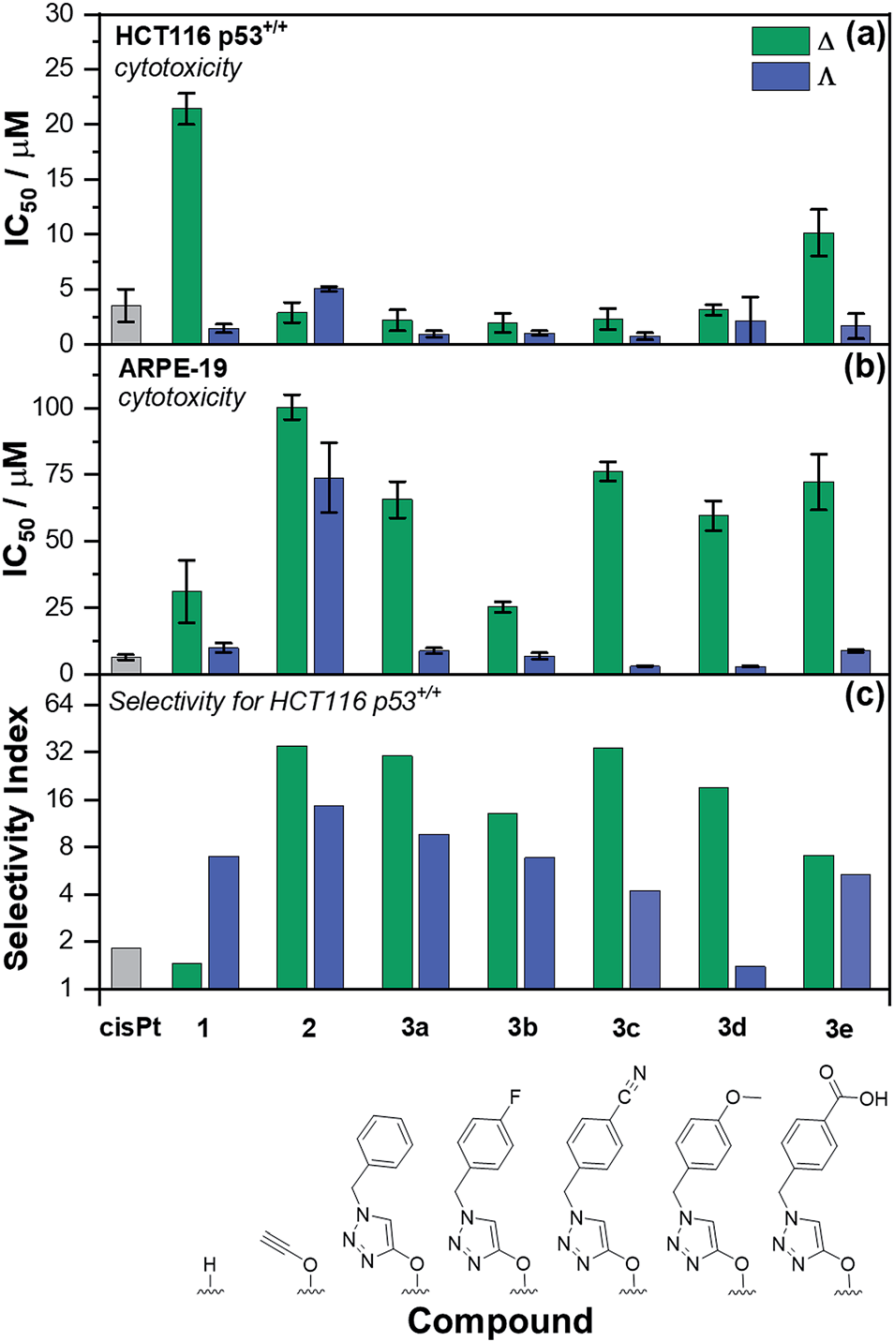
Antiproliferative activity of triplex metallohelices in cancer and non-cancer cells. The half maximum inhibitory concentration (IC_50_) values are from triplicate measurements using the MTT assay, dosing for 96 h against: (a) HCT116 p53^+/+^ colon cancer cells; (b) ARPE-19 (non-cancerous) cells. The selectivity index (c) *i.e.* [mean IC_50_(ARPE-19)]/[mean IC_50_(HCT116 p53^+/+^)] for the control drug cisplatin (cisPt), the “parent” triplex [Fe_2_**L^1^**_3_]Cl_4_, alkyne triplex [Fe_2_**L^2^**_3_]Cl_4_ and CuAAC-derived systems [Fe_2_**L^3a–e^**_3_]Cl_4._

The panel of Fe(ii) compounds of Fig. 1 were initially evaluated alongside cisplatin for potency against the human epithelial colorectal cancer cell line HCT116 p53^+/+^ (wild-type p53) and non-cancerous human epithelial retinal pigment cells (ARPE-19) (Fig. 2).^26^

The modest potency of parent compound Δ-[Fe_2_**L^1^**_3_]Cl_4_ against HCT116 p53^+/+^ leads to a poor selectivity index: the ratio of IC_50_ values of ARPE-19 cells to HCT116 p53^+/+^ cells (see Fig. 2 – note log scale). While this is higher for the Λ enantiomer, the performance of the alkyne derivatives [Fe_2_**L^2^**_3_]Cl_4_ is much improved, with potencies similar to that of cisplatin against HCT116 p53^+/+^ but giving rather better selectivity indices. The “clicked” metallohelices [Fe_2_**L^3a–e^**_3_]Cl_4_ perform better still. They are all more potent than cisplatin against the HCT116 p53^+/+^ colon cancer cells, with the exception of the more moderately active tricarboxylic acid Δ-[Fe_2_**L^3e^**_3_]Cl_4_. These complexes also exhibit enantioselectivity; in all cases the Λ_Fe_ compounds are more active than Δ_Fe_ [Fig. 2a]. Pleasingly, the new metallohelices are all significantly less toxic to non-cancerous ARPE-19 cells with the Δ_Fe_ enantiomers being substantially less toxic than Λ_Fe_ [Fig. 2b]. The resulting selectivity indices show that the Δ_Fe_-enantiomers are all more selective than their Λ_Fe_ analogues; both Δ-[Fe_2_**L^3a^**_3_]Cl_4_, and Δ-[Fe_2_**L^3c^**_3_]Cl_4,_ have a selectivity indices for these particular cell lines of >30. On the basis that the former is the simpler compound, it was selected from the click derivatives for further study.

**Fig. 3:**
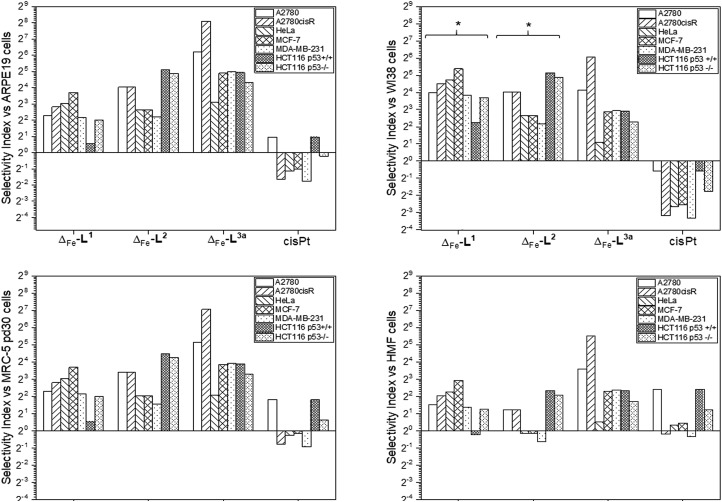
Antiproliferative selectivity indices of triplex metallohelices for cancer *vs.* non-cancer cells. Selectivity index = mean IC_50_ ratio: (non-cancer cell line/cancer cell line), IC_50_ values as listed in Table 1. *Minimum selectivity indices plotted as IC_50_ (WI-38) > 100 μM.

Accordingly, the compounds Δ-[Fe_2_**L^1^**_3_]Cl_4_, Δ-[Fe_2_**L^2^**_3_]Cl_4_ and Δ-[Fe_2_**L^3a^**_3_]Cl_4_ were screened against a larger panel of cell lines of different tissue origins (colon, ovarian, cervical and breast cancers, plus a range of non-cancer). The data are given in Table 1 and the 112 selectivity indices determined (four compounds, seven cancer cell lines, four non-cancer) are plotted in Fig. 3. The metallohelices were without exception more active against each of the seven cancer cell lines than the four different non-cancer cell lines. Furthermore, they display an overwhelmingly favourable selectivity compared with the clinically used chemotherapeutic agent cisplatin. In most instances the compound Δ-[Fe_2_**L^3a^**_3_]Cl_4_ was the most selective.

**Table 1:** Cell viability (IC_50_ mean values, μM) of the investigated compounds. Cell survival was evaluated using the MTT^*a*^ assay

	Cell line	Compound			
		Δ-[Fe_2_**L^1^**_3_]Cl_4_	Δ-[Fe_2_**L^2^**_3_]Cl_4_	Δ-[Fe_2_**L^3a^**_3_]Cl_4_	Cisplatin
Cancer cell lines	HCT116 p53^+/+^ (colon)	21.4 ± 1.4^*b*^	2.9 ± 0.9^*b*^	2.2 ± 1.0^*b*^	3.3 ± 0.4^*b*^
	HCT116 p53^–/–^ (colon)	7.7 ± 3.7^*b*^	3.4 ± 0.2^*b*^	3.3 ± 0.3^*b*^	7.5 ± 0.7^*b*^
	A2780 (ovarian)	6.38 ± 0.1	6.1 ± 0.8	0.9 ± 0.2	3.3 ± 0.2
	A2780cisR (ovarian)	4.43 ± 0.1	6.1 ± 0.3	0.24 ± 0.02	20 ± 3
	HeLa (cervical)	3.8 ± 0.9	16 ± 6	7.6 ± 0.5	14.0 ± 0.9
	MCF-7 (breast)	2.4 ± 0.4	16 ± 2	2.2 ± 0.2	12.9 ± 0.6
	MDA-MB-231 (breast)	7 ± 1	22 ± 1	2.1 ± 0.2	22 ± 2
Non-cancer cell lines	ARPE-19 (retinal)	31 ± 12^*b*^	100 ± 5^*b*^	66 ± 7^*b*^	6.4 ± 1.0^*b*^
	MRC-5 pd30 (lung)	31 ± 6	65 ± 5	32 ± 5	12 ± 1
	HMF (breast)	18 ± 5^*b*^	14 ± 2^*b*^	11 ± 1^*b*^	18 ± 2^*b*^
	WI-38 (lung)	>100^*b*^	>100^*b*^	16 ± 3^*b*^	2.2 ± 0.8^*b*^

^*a*^The experiments were performed in triplicate or quadruplicate. The cells were treated with the investigated compounds for 72 h, unless otherwise stated. The results are expressed as mean values ± SD from three or four independent experiments.

^*b*^Cells were treated for 96 h.

We noted that while conversion of alkyne Δ-[Fe_2_**L^2^**_3_]Cl_4_ to benzyltriazole Δ-[Fe_2_**L^3a^**_3_]Cl_4_ had little effect on potency in the HCT116 colon cancer cells, potency in the ovarian and breast cancer cell lines was increased ∼7 to 25 fold (Table 1). In contrast, activity against three of the four non-cancer cell lines was only modestly increased (by <1.3 fold in HMF, ∼1.5 fold in ARPE19 and ∼2 fold in MRC-5 pd30). The click modification resulted in a ∼4-fold increase in potency towards the cisplatin-resistant ovarian cancer cells (A2780cisR) compared to the cisplatin-sensitive parental cells indicating a lack of cross-resistance and, unsurprisingly, a distinct mechanism of action to that of the DNA “alkylator” cisplatin.

The p53 tumour suppressor gene is one of the most frequently mutated in cancer, commonly causing increased resistance to chemotherapeutic drugs. While accordingly here cisplatin was found to be >2 fold less active towards HCT116 p53^–/–^ cells than the p53^+/+^ isogenic clones (Table 1), such a loss of potency was not observed for any of the Δ-metallohelices.

### Mechanistic studies

**Fig. 4:**
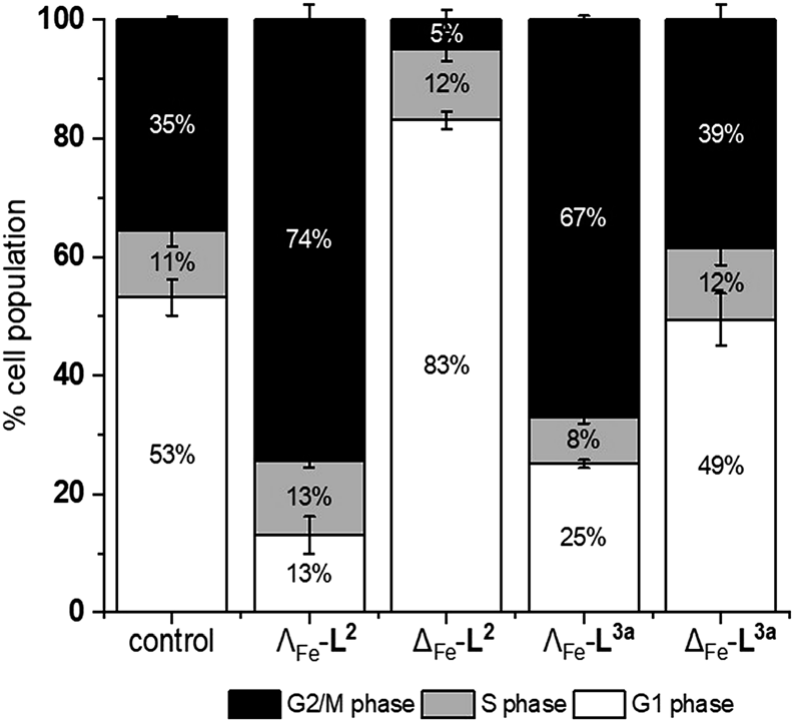
Effects of triplex metallohelices on the cell cycle. Analysis by flow cytometry of propidium iodide-stained cells and quantification of the percentage of the cell population in different stages of the cell cycle for untreated HCT116 p53^+/+^ cells, and following incubation with the metallohelices for 24 h (at a concentration of twice the 96 h IC_50_). Remarkably the Λ_Fe_ compounds cause accumulation in G2/M phase while for Δ_Fe_-**L^2^** it is G1. The Δ_Fe_ compound of **L^3a^** has little effect.

We noted that the enantiomer potencies for [Fe_2_**L^2^**_3_]Cl_4_ (*i.e.* Δ > Λ) against cancer cells were reversed for [Fe_2_**L^3a^**_3_]Cl_4_ and the other click derivatives. We thus compared the effects of the enantiomer pairs on the cell cycle profile of asynchronously growing cells. Striking enantiomeric and structure-dependent differences were observed (Fig. 4) implying different mechanisms of action. Further, since the high selectivity index compound Δ-[Fe_2_**L^3a^**_3_]Cl_4_ did not induce significant alteration in the cell cycle profile, the induction of cell death *via* apoptosis, a target of many anticancer drug treatments,^27^–^29^ was investigated. To our surprise, induction of apoptosis was not observed *via* a membrane phosphatidylserine (PS) assay^30^ for HCT116 p53^+/+^ cells except after prolonged exposure at 2 × IC_50_ (ESI Fig. S26†).

**Fig. 5:**
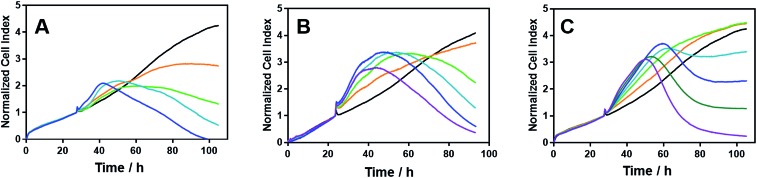
Time-dependent cellular response profiles of A2780 cancer cells treated with increasing concentrations of the metallohelices; cell index in arbitrary units is a measure of cell sensor impedance: (A) Δ-[Fe_2_**L^1^**_3_]Cl_4_ (lines: orange – 4 μM; green – 8 μM; turquoise – 20 μM; blue – 40 μM); (B) Δ-[Fe_2_**L^2^**_3_]Cl_4_ (lines: orange – 2 μM; green – 4 μM; turquoise – 8 μM, blue – 20 μM; magenta – 40 μM); (C) Δ-[Fe_2_**L^3a^**_3_]Cl_4_ (lines: orange – 0.125 μM; green – 0.25 μM; turquoise – 0.5 μM; blue – 0.8 μM; dark green – 1.6 μM; magenta – 3.2 μM). The medium containing the tested compounds was added after 27.5 h of incubation.

**Fig. 6:**
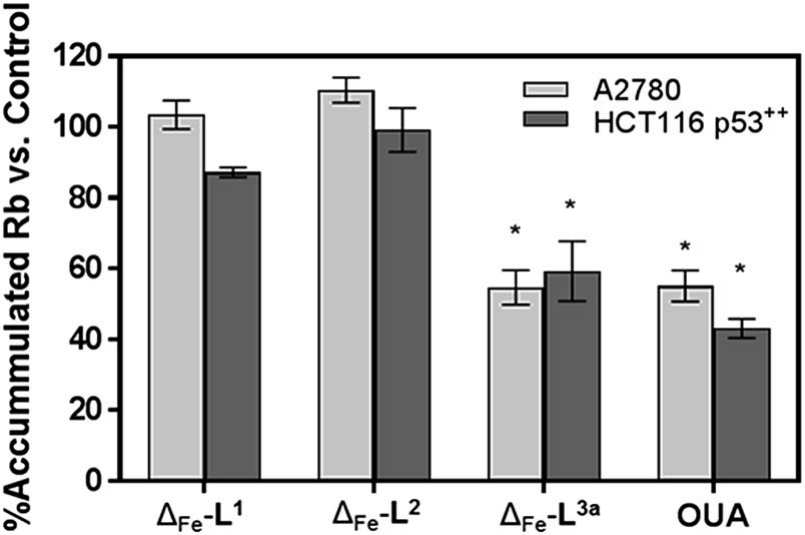
Accumulation of Rb in cells treated with metallohelices. A2780 and HCT116 p53^+/+^ cells were treated with metallohelices and ouabain (10 μM) for 6 h – the short incubation time was used to secure cell viability and to mainly detect the upstream effects of the applied drug – followed by RbCl (5.4 mM) for 3 h. Uptake of Rb^+^ was determined by ICP-MS. All results are expressed as the mean ± SD from three independent experiments. Asterisks indicate a significant difference from untreated control (100%) with *p < 0.001 calculated by using 2 way ANOVA.

Time-dependent cellular response profiles (TCRPs) produced by impedance-based monitoring provides predictive mechanistic information for the action of small molecules.^31^–^33^ Ovarian cancer cells (A2780) that have been treated with Δ-[Fe_2_**L^1^**_3_]Cl_4_, Δ-[Fe_2_**L^2^**_3_]Cl_4_ and Δ-[Fe_2_**L^3a^**_3_]Cl_4_ show clearly distinct TCRPs (Fig. 5). For the parent metallohelix Δ-[Fe_2_**L^1^**_3_]Cl_4_ the initial rise in Cell Index (CI) impedance signal is less apparent than for other compounds and the period of signal elevation is the shortest. For the alkyne Δ-[Fe_2_**L^2^**_3_]Cl_4_ the CI signal increases to *ca.* 1.7× that of the control and the peak is relatively broad, the signal decreasing steadily over the measurement period. For the benzyl triazole derivative Δ-[Fe_2_**L^3a^**_3_]Cl_4_ the CI signals reach a much sharper dose-dependent maximum. A TCRP profile database search^31^ indicated a similarity with that for compounds that inhibit Na^+^/K^+^ stimulated ATPases; a highly conserved integral cell membrane pump expressed in virtually all cells of higher organisms that maintains ionic concentration gradients.^34^ An established rubidium-based assay^35^ subsequently showed that Δ-[Fe_2_**L^3a^**_3_]Cl_4_ did indeed inhibit uptake of the cation in A2780 and HCT116 p53^+/+^ cell lines under these conditions, by 35–47% (Fig. 6). This performance is comparable with that of the known potent Na^+^/K^+^-ATPase inhibitor ouabain^36^ (39–57% inhibition). In contrast Δ-[Fe_2_**L^1^**_3_]Cl_4_ and Δ-[Fe_2_**L^2^**_3_]Cl_4_ had little if any effect.

### Antimetastatic properties of metallohelices

Colorectal cancer is one of the four most common causes of cancer deaths,^37^ and in 90% of instances mortality is ascribed to metastasis, for which there are currently no effective treatments.^38^,^39^ Ouabain has been reported to inhibit cancer cell migration^40^–^43^ and to possess antimetastatic activity through its inhibition of Na^+^/K^+^ ATPase.^43^ On the basis of the mechanistic discoveries above we investigated the effects of Δ-[Fe_2_**L^3a^**_3_]Cl_4_ on important steps in the process of metastasis^44^–^46^ in the colon cancer cell line HCT116 p53^+/+^.

**Fig. 7:**
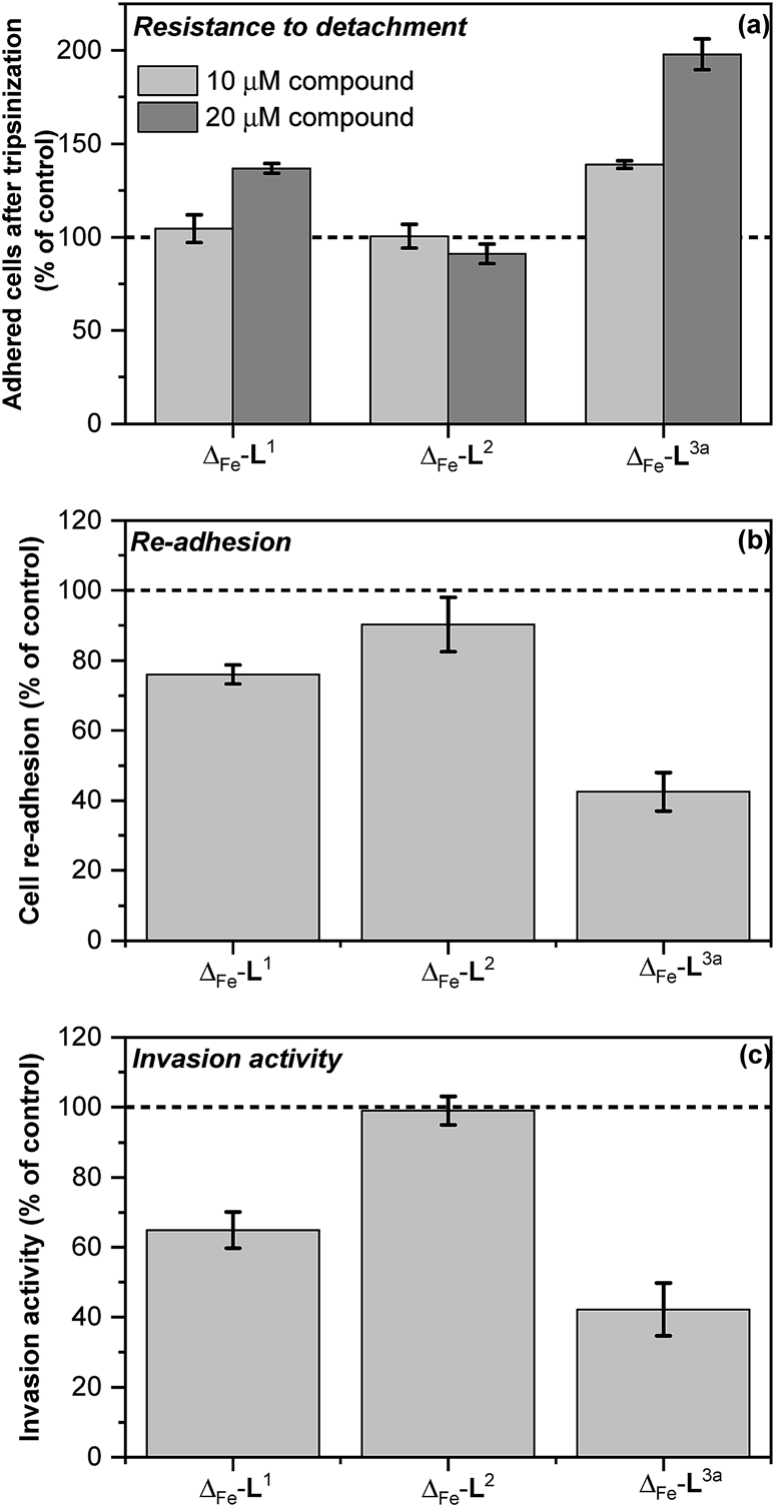
*In vitro* antimetastatic activity of metallohelices (a) effects of the metallohelices on cancer cell adhesion and resistance to detachment through use of diluted trypsin enzyme; cells were treated with the indicated metallohelices at 10 μM and 20 μM respectively for 3 h prior to addition of trypsin (b) cell re-adhesion assay; cells detached following trypsinization (and 3 h pre-treatment with the metallohelices at 10 μM) were assayed for their ability to re-adhere within 30 min, (c) cell invasion assay; cells were treated with the metallohelices at equitoxic (2 × IC_50_) concentration for 2 h, followed by assessment of invasion through Matrigel (see Methods in ESI†).The results are expressed as the mean ± SD from three independent experiments. Asterisks indicate a significant difference from untreated control (100%) with *p < 0.05 or **p < 0.001 calculated by using 2 way ANOVA.

We modelled the detachment of cancer cells from a primary tumour by an *in vitro* assay of cell resistance to trypsinization.^47^–^49^ HCT116 p53^+/+^ cells grown in monolayer were treated with the investigated compounds for 3 h and then subjected to a diluted trypsin solution. The number of cells that resisted the treatment (*i.e.* remained attached to the surface) was evaluated by the sulforhodamine B (SRB) assay. Treatment with Δ-[Fe_2_**L^1^**_3_]Cl_4_ reduced detachment only at higher concentrations [top panel in Fig. 7] and Δ-[Fe_2_**L^2^**_3_]Cl_4_ had no significant effect. In contrast, treatment of cells with Δ-[Fe_2_**L^3a^**_3_]Cl_4_ significantly impeded their detachment.

Re-attachment of cancer cells to tissue at a ‘new’ site in the body (the site of secondary tumour formation)^50^ was modelled in a re-adhesion assay.^51^,^52^ Cells were treated with 10 μM compound for 3 h (a non-toxic dose), detached with trypsin and re-seeded at a density of 2 × 10^4^ cells per well. The number of cells attached after 30 min incubation was determined by the SRB assay [middle panel in Fig. 7]. Compounds Δ-[Fe_2_**L^1^**_3_]Cl_4_ and Δ-[Fe_2_**L^3a^**_3_]Cl_4_ reduced cell re-adhesion by 24% and 58% respectively.

Cancer cell invasion defines the ability of cells to infiltrate tissue. Using a Matrigel™ transwell assay^53^,^54^,^70^ (see ESI†) it was shown that while Δ-[Fe_2_**L^2^**_3_]Cl_4_ had no effect, Δ-[Fe_2_**L^1^**_3_]Cl_4_ and Δ-[Fe_2_**L^3a^**_3_]Cl_4_ reduced HCT116 p53^+/+^ invasive ability by 35% and 58% [bottom panel in Fig. 7].

**Fig. 8:**
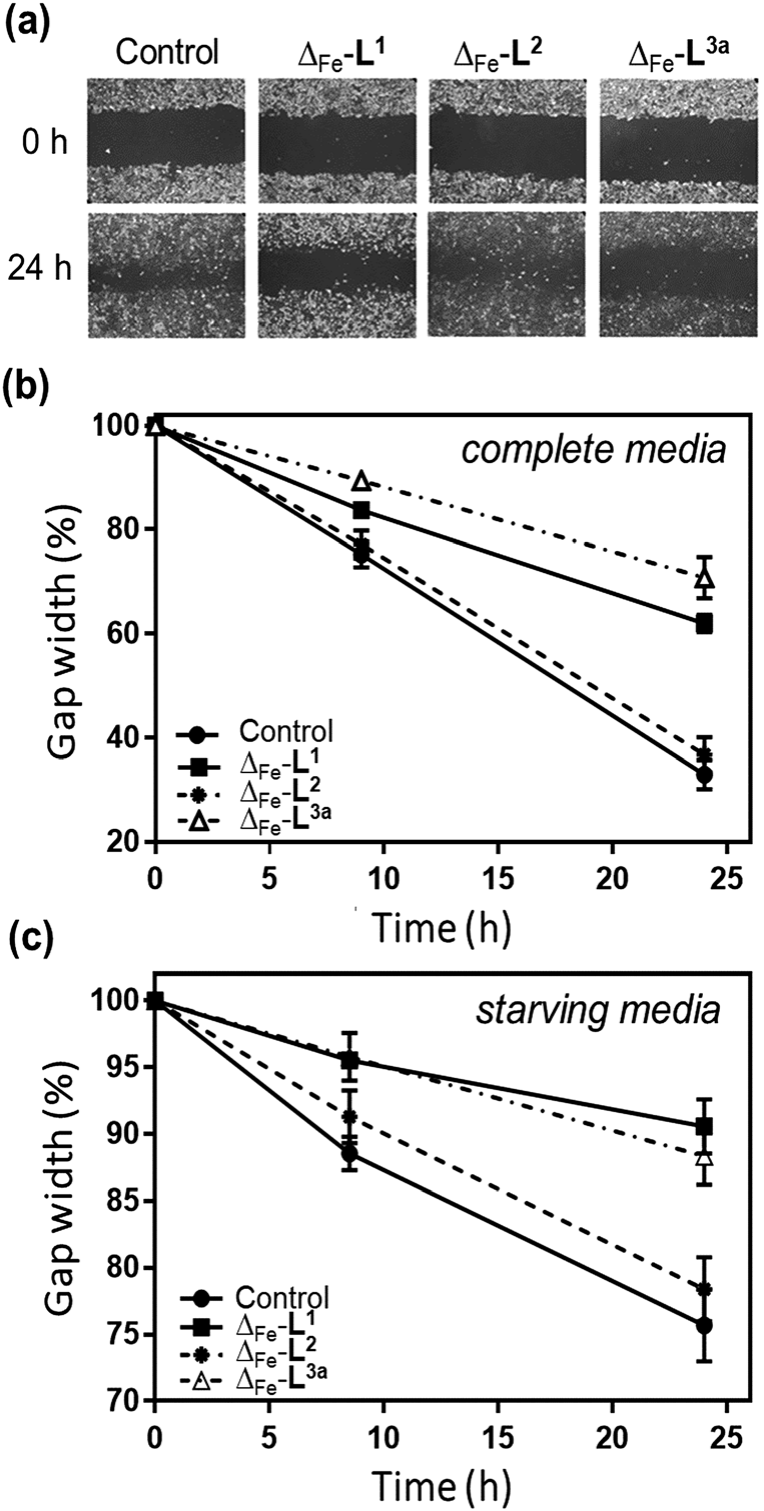
Wound healing assay of metallohelices. (a) Representative scratch assay images of HCT116 p53^+/+^ cancer cells and the effect of treating with metallohelices (at IC_50_ concentrations) on scratch closure; images at 0 and 24 h following insertion of a scratch. (b) Quantification of scratch closure at 0, 8.5 and 24 h with cells cultured in complete media (c) a similar result was obtained in starving medium conditions [serum-deprived medium (1% BSA)] indicating that the suppression of wound-healing results at least in part from anti-migration/invasion rather than being due to cell proliferation resulting in closure of the scratch.

The overall ability of the compounds to influence cancer cell migration and invasion was assessed by wound healing assay (scratch gap closure)^55^,^72^ [Fig. 8a]. While in a control sample a scratch in a monolayer of HCT116 p53^+/+^ cells had healed in 24 h to 33%, the presence of non-toxic doses of Δ-[Fe_2_**L^1^**_3_]Cl_4_ or Δ-[Fe_2_**L^3a^**_3_]Cl_4_ suppressed healing, leaving 62% and 71% respectively of the wound open. Cells treated with Δ-[Fe_2_**L^2^**_3_]Cl_4_ resembled the control.

### Cancer stem cell (CSC) targeting

CSCs^56^ represent a reservoir of resistant and highly aggressive cancer cells that can remain when the bulk of a tumour has been eradicated, leading to formation of secondary or tertiary tumours.^57^–^60^ The failure to eliminate or inhibit CSCs is thereby a major cause of failure of existing anti-cancer treatments^61^–^63^ and is a key challenge to improving patient outcomes.^64^–^67^

**Fig. 9:**
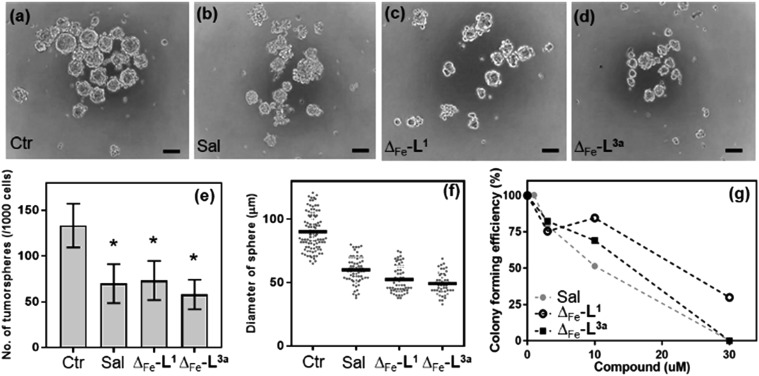
Growth inhibitory effects in HCT116.CD133^+^ cancer stem cells. Representative microscopy images of the HCT116.CD133^+^ colonospheres in the absence (a) and presence of salinomycin (b), Δ-[Fe_2_**L^1^**_3_]Cl_4_ (c), and Δ-[Fe_2_**L^3a^**_3_]Cl_4_ (d), treated at their respective IC_30_ values for 6 days (scale bar: 100 μm). Quantification of colonosphere formation (e and f) under the same conditions. Clonogenic assay on the HCT116.CD133^+^ (g) showing the colony forming efficiency (*i.e.* the number of colonies that formed post-drug treatment, with respect to the number of cells seeded) after treatment with different concentrations of salinomycin, (grey circles), Δ-[Fe_2_**L^1^**_3_]Cl_4_ (black open circle), and Δ-[Fe_2_**L^3a^**_3_]Cl_4_ (black squares) for 48 h, following growth for 8 days. Data represent the mean value and SD from three independent experiments. *p* < 0.01, *versus* control.

The HCT116 p53^+/+^ cells^68^,^69^ possess a cancer stem cell fraction capable of forming colonospheres from single cells, and have been utilized in several studies as a CSC model.^70^–^72^ The HCT116 p53^+/+^ cells were treated with Δ-[Fe_2_**L^1^**_3_]Cl_4_ and Δ-[Fe_2_**L^3a^**_3_]Cl_4_ at their respective IC_30_ concentrations for 72 h, and subsequently cultured as single cell suspensions in serum-free media. While both Δ-[Fe_2_**L^1^**_3_]Cl_4_ and Δ-[Fe_2_**L^3a^**_3_]Cl_4_ were found to inhibit colonosphere formation in HCT116 p53^+/+^ under these conditions, Δ-[Fe_2_**L^3a^**_3_]Cl_4_ in particular was significantly more effective than the CSC-selective^73^–^76^ drug salinomycin (ESI Table S3 and Fig. S27, S28†). Subsequently, significant colonosphere inhibition by both Δ-[Fe_2_**L^1^**_3_]Cl_4_ and Δ-[Fe_2_**L^3a^**_3_]Cl_4_ was also observed in CSC-enriched cells (HCT116.CD133^+^). The compounds were equally or slightly more effective than salinomycin in reduction in the number and average size of colonospheres [Fig. 9a–f].

Next, we compared the effects of Δ-[Fe_2_**L^1^**_3_]Cl_4_, Δ-[Fe_2_**L^3a^**_3_]Cl_4_ and salinomycin on HCT116.CD133^+^ and HCT116.CD133^–^ cells (Fig. 9 and ESI Fig. S30†) these data indicate that Δ-[Fe_2_**L^1^**_3_]Cl_4_ and salinomycin effectively inhibit colonosphere formation from HCT116.CD133^+^ cells, but do not significantly inhibit colonosphere formation from HCT116.CD133^–^ cells. This result can be interpreted to mean that Δ-[Fe_2_**L^1^**_3_]Cl_4_ and salinomycin are CSC-selective agents. On the other hand, Δ-[Fe_2_**L^3a^**_3_]Cl_4_ also effectively inhibits colonosphere formation from both HCT116.CD133^+^ and HCT116.CD133^–^ cells, although being less effective in CD133^–^ negative cells. The reasonable explanation of the latter result is that Δ-[Fe_2_**L^3a^**_3_]Cl_4_ exhibits a lower selectivity for CSCs than Δ-[Fe_2_**L^1^**_3_]Cl_4_ and salinomycin being able to effectively kill both differentiated cancer cells and CSCs. As effective cancer treatments must attack both rapidly-dividing differentiated (non-stem) cancer cells and CSCs, Δ-[Fe_2_**L^3a^**_3_]Cl_4_ appears to be a promising candidate compound able to overcome limitations connected with the use of a number of conventional chemotherapeutics.

The data in Fig. 9g arise from a clonogenic assay that examines the capability of a single cell to grow into a large colony through clonal expansion.^77^ CSC-enriched HCT116.CD133^+^ cells incubated for 48 h with 30 μM Δ-[Fe_2_**L^3a^**_3_]Cl_4_, exhibited no surviving cells after being allowed to grow for 8 d; an effect comparable to that of conventional salinomycin. Δ-[Fe_2_**L^1^**_3_]Cl_4_ was less effective.

The selective anti-cancer stem cell effects of Δ-[Fe_2_**L^3a^**_3_]Cl_4_ were further demonstrated (ESI Table S4†). The IC_50_ of 1.21 ± 0.25 μM in CSC-enriched HCT116.CD133^+^ is around half in HCT116 p53^+/+^ under the same conditions; a better differential than that observed with salinomycin.

The studies aimed at the mechanism of action of the investigated metallohelices on CSCs is in progress in our laboratories and will be published in a separate article. Nevertheless, we demonstrate in our manuscript that Δ-[Fe_2_**L^1^**_3_]Cl_4_ and Δ-[Fe_2_**L^3a^**_3_]Cl_4_ were equally or slightly more effective in killing CSCs than CSC-selective salinomycin (ESI Fig. S30†). It was shown recently^76^ that nucleolin is likely a salinomycin-binding target and a critical regulator involved in human neuroblastoma CSC activity. It is, therefore, possible, due to the similar effectivity of the investigated metallohelices (Δ-[Fe_2_**L^1^**_3_]Cl_4_ and Δ-[Fe_2_**L^3a^**_3_]Cl_4_) and salinomycin to kill HCT116.CD133^+^ CSCs, that their binding to nucleolin may also be responsible for the anticancer and anti-CSC like cell activities of Δ-[Fe_2_**L^1^**_3_]Cl_4_ and Δ-[Fe_2_**L^3a^**_3_]Cl_4_.

## Conclusions

One of the key advantages of our metallohelix assemblies is their great stability with respect to dissociation or hydrolysis. This sort of stability will be necessary in order for any such compound to find its way into clinical use, and has here allowed the use of an extremely efficient CuAAC post-assembly modification of the triplex alkynyl enantiomers [M_2_**L^2^**_3_]. This reaction gave rapid access to a new range of functionalised compounds and led to the discovery of Δ-[Fe_2_**L^3a^**_3_]Cl_4_; a compound with an unusual combination of pharmacological properties.

In a study involving an unusually wide range of cancer and non-cancer cell types, the new “clicked” compounds demonstrated enhanced potency. More importantly however the selectivity for cancerous over non-cancerous cells was greatly improved; this bodes well for the development of compound with a wider therapeutic window than conventional chemotherapeutic agents such as cisplatin. Further, in contrast to observations for conventional drugs,^78^ a p53 mutated cell line did not show resistance to the compound.

While some part of the observed selectivity of Δ-[Fe_2_**L^3a^**_3_]^4+^ probably arises from electrostatic targeting of the anionic outer leaflet of cancer cells (as is proposed for cationic anticancer peptides^79^–^82^) the enantiomer effects observed here point to greater subtlety. The Δ compound is much more selective than Λ for cancer cells, and this is reflected in a remarkable difference in cell cycle arrest observations. It was also discovered that Δ-[Fe_2_**L^3a^**_3_]Cl_4_ inhibits Na^+^/K^+^ ATPase activity with potency comparable to that of the conventional inhibitor ouabain.

These mechanistic observations led to the discovery of a remarkable array of properties of Δ-[Fe_2_**L^3a^**_3_]Cl_4_ alongside its high potency and selectivity. The compound suppresses key metastatic capacities of cancer cells, reducing their ability to detach from other cells, migrate, invade and re-adhere elsewhere. Compound Δ-[Fe_2_**L^3a^**_3_]Cl_4_ exhibits selective toxicity for colon cancer stem cell-enriched cell populations, challenging some of the most selective compounds of any kind identified to date. Additionally, Δ-[Fe_2_**L^3a^**_3_]Cl_4_ inhibits the formation of colonospheres by specifically targeting CD133-positive, CSC-like cells. Thus Δ-[Fe_2_**L^3a^**_3_]Cl_4_ is identified as a potential lead compound for investigation as a selective anti-cancer, anti-metastatic and CSC-targeting drug.

More generally, while the precise molecular basis for the difference between the benzyltriazolyl-clicked compounds and the parent enantiomers is not known, it is clear that these discoveries would be much harder to unearth without access to post-assembly modification. Further, we now know that metallohelices, like conventional medicinal compounds, respond to chemical modification in such a manner as to facilitate optimization of biological and physicochemical properties.

## Experimental

Full details of synthesis, characterization and anti-cancer experiments are provided in the electronic ESI.† Outlines of key procedures are detailed in the following.

### Synthesis of water-soluble alkyne-functionalised triplex metallohelices

Anhydrous iron(ii) chloride (2 equiv.) was added to a stirred solution of the desired chiral amine, 2-([2,2′-bipyridin]-5-ylmethoxy)-1-phenylethan-1-amine (3 equiv.) and 5-(prop-2-yn-1-yloxy)picolinaldehyde (3 equiv.) in methanol (20 ml) at ambient temperature to give a purple solution that was then heated to reflux (85 °C) for 48 h. The mixture was allowed to cool to ambient temperature, filtered through a Celite plug, and the solvents were removed *in vacuo* to give HHT-[Fe_2_**L^2^**_3_]Cl_4_ as a dark purple solid (>95% yield).

### Post-assembly modification of triplex metallohelices

HHT-[Fe_2_**L^2^**_3_]Cl_4_ (1 equiv.) and the chosen benzyl azide (4.5 equiv.) were dissolved in methanol (10 ml) in the presence of copper(i) iodide (0.1 equiv.). The reaction mixture was heated at 65 °C for 18 h under inert argon atmosphere. After cooling to ambient temperature, the suspension was filtered to remove copper salts and the purple product HHT-[Fe_2_**L^3^**_3_]Cl_4_ was isolated by the addition of ethyl acetate. NMR, infrared and high-resolution mass spectrometric data were consistent with the proposed formulations. CD spectra of enantiomers in methanol were equal and opposite.

### Antiproliferative activity (MTT assay)

The human ovarian carcinoma cisplatin-sensitive A2780 cells, cisplatin-resistant A2780cisR (a cisplatin-resistant variant of A2780 cells), human cervical carcinoma HeLa cells, human breast cancer MCF-7 cells and human colorectal carcinoma cells HCT-116 (for experiments performed at the Czech Academy of Sciences) were kindly supplied by Professor B. Keppler, University of Vienna (Austria). Human colon carcinoma cells expressing p53 (HCT116 p53^+/+^) were a kind gift of Dr M. Brazdova, Institute of Biophysics, Brno (Czech Republic). Highly invasive breast carcinoma MDA-MB-231 cells and human MRC-5 pd30 cells derived from normal lung tissue were purchased from the European collection of authenticated cell cultures (ECACC) (Salisbury, UK). Isogenic clones of p53^+/+^ and p53^–/–^ HCT116 colon carcinoma cells for experiments performed at the University of Huddersfield were a kind gift from Bert Vogelstein (Johns Hopkins University, Maryland, USA). ARPE19 and WI38 cells were both purchased from ATCC (American Type Culture Collection) and HMF cells were purchased from ScienCell Research Laboratories, Inc.

Cells were incubated in 96-well plates in complete cell medium, containing DMEM supplemented with 10% fetal calf serum and l-glutamine (2 mM). Plates were incubated for 24 h at 37 °C in an atmosphere of 5% CO_2_, before drug exposure, then incubated for 96 h with drug. A volume of 3-(4,5-dimethylthiazol-2-yl)-2,5-diphenyltetrazolium bromide (MTT) solution (0.5 mg ml^–1^) was added to each well and incubated for a further 4 h. These solutions were removed, dimethyl sulfoxide was added to each well, and the absorbance at 540 nm was read using a Thermo Scientific Multiskan EX microplate photometer. The IC_50_ values were determined from a plot of percentage cell survival against drug concentration (in μM). All assays were conducted in triplicate and the mean IC_50_ ± standard deviation was determined.

### Cell cycle assay

PBS (300 μL) containing propidium iodide (50 μg ml^–1^) and RNAse A (80 μg ml^–1^) was added to drug-treated cells before incubation for 30 min and FACS analysis. The assay was repeated four times with each compound and the mean percentage of cells in each phase ± standard deviation was determined. Red fluorescence was observed at 488 nm excitation by flow cytometry and data were analysed using Flowjo V10.

### Time-dependent cellular response profiling

The impedance monitoring of cell growth was performed using an xCELLigence RTCA SP Real-time cell analyser. Cells were added and grown for 22–28 h, before tested compounds were added to the medium at varying concentrations. The impedance was measured for an additional 80 h.

### Rubidium-based assay

Cells were seeded in 6-well plates and incubated for 24 h at 37 °C in an atmosphere of 5% CO_2_. Cells were then incubated with drug for 6 h, and subsequently the medium was removed and cells were washed with PBS. Cells were then incubated with RbCl (5.4 mM) for 3 h, washed and counted. Rubidium content was determined by ICP-MS.

### Wound healing assay

Cells were seeded in 12-well plates and incubated in complete medium (10% FBS/DMEM/gentamycin) at 37 °C in an atmosphere of 5% CO_2_. The medium was removed and the bottom of the well was scratched with a 10 μL pipette tip (550–650 μm gaps). Wells were washed to remove detached cells before drug was added, dissolved in complete or starving medium, and plates were incubated for 24 h. Images were taken at several time intervals post scratching, and automated analysis was performed using TScratch software (MATLAB).

### Inhibition of colonosphere formation

Cells were treated with drug (IC_30_ concentration) for 72 h, washed, harvested with StemPro Accutase, and plated in ultra-low attachment 96-well culture plates (300 cells per well). Cells were cultured in for 6 days without disturbing the plates or replenishing the medium and the number/size of spheres were determined using an inverted microscope.

### Clonogenic assays

Cells were seeded in ultra-low attachment 6-well plates and cultured for 4 days, to allow pre-spheroid formation. Cells were treated with drug for 48 h, and dissociated into single cell suspensions using StemPro Accutase. Single cells were seeded in normal 6-well plates (3000 cells per well) and cultured for 8 days. Methylene blue solution (1% in water : methanol 1 : 1) was added for 30 min to stain the formed colonies, excess was washed out, and colonies containing >50 cells were counted.

## Conflicts of interest

There are no conflicts to declare.

## Supplementary Material

Supplementary information
