# Supplementary material for: Discovery of selective, antimetastatic and anti-cancer stem cell metallohelices via post-assembly modification
Source: Chem Sci. 2019 Jul 18;10(37):8547–57. doi: 10.1039/c9sc02651g (PMC6839601; doi:10.1039/c9sc02651g)
Supplement: Supplementary file 1 [file SC-010-C9SC02651G-s001.pdf]

Electronic Supplementary Material (ESI) for Chemical Science.
This journal is © The Royal Society of Chemistry 2019

Electronic Supplementary Information

Discovery of selective, antimetastatic and anti-cancer stem cell metallohelices via post-assembly modification

Authors

Hualong Song,[a] Nicola Rogers,[a] Simon J. Allison,[b] Viktor Brabec,*[c] Hannah Bridgewater,[d] Hana Kostrhunova,[c] Lenka Markova,[c] Roger M. Phillips,*[,b] Emma C. Pinder,[b] Samantha L. Shepherd,[b] Lawrence S. Young,[d] Juraj Zajac,[c] and Peter Scott*[,a]

[a] Department of Chemistry, University of Warwick, Coventry CV4 7AL, UK

[b] School of Applied Sciences, University of Huddersfield, Huddersfield, HD1 3DH

[c] The Czech Academy of Sciences, Institute of Biophysics, Kralovopolska 135, CZ-61265 Brno, Czech Republic

[d] Warwick Medical School, University of Warwick, Coventry CV4 7AL, UK

Contents

1. Synthesis S3
2. NMR Spectra S23
3. ICP-MS [Cu] test for S_c,Λ_{Fe},HHT-[$Fe_2L^{3b}_3$]Cl_4. .... S36
4. Stability tests using NMR: S37
5. Circular Dichroism Spectra S38
6. Stability study S39
7. High Resolution ESI mass spectra S40
8. Anticancer Experiments S43
8.1 Chemosensitivity (MTT assay) S43
8.2 Cell Cycle assay S44
8.3 Apoptosis assay (Annexin V assay) S47
8.4 Real-time cell growth monitoring S48
8.5 Rubidium based assay for detection of Na^+/K^+ ATPase activity S48
9. Antimetastatic study S48
9.1 Resistance to detachment assay S48
9.2 Re-adhesion assay S49
9.3 Cell invasion test S49
9.4 Wound healing assay S50
10. Anti-cancer stem cell study S52
10.1 Inhibition of HCT116 colonsphere formation S52
10.2 Separation of HCT116.CD133$^+$ cells by magnetically activated cell sorting (MACS) S53
10.3 Flow cytometric analysis of surface expression levels S53
10.4 Inhibition of HCT116.CD133$^+$ colonsphere formation S54
10.5 Cytotoxicity in HCT116.CD133$^+$ cells, SRB assay S55
10.6 Clonogenic assay / Colony formation assay S55
11. Supplementary References S56

1. Synthesis

All solvents and chemicals purchased from commercial sources (Sigma-Aldrich, Acros, Fisher Scientific or Alfa Aesar) were used without further purification unless otherwise stated. Sodium hydride dispersions in mineral oil were placed in a Schlenk vessel under an inert atmosphere and washed three times with diethyl ether to remove the oil, then dried and stored under argon in an MBraun dry box. Where appropriate, reactions were carried out under argon using a dual manifold argon/vacuum line and standard Schlenk techniques or in an MBraun dry box. Necessary solvents were dried by heating to reflux for 3 d under dinitrogen over the appropriate drying agents (potassium for tetrahydrofuran, sodium/potassium alloy for diethyl ether, and calcium hydride for acetonitrile and pyridine) and degassed before use. Tetrahydrofuran and diethyl ether were additionally pre-dried over sodium wire. Dried solvents were stored in glass ampoules under argon. All glassware and cannulae were stored in an oven at > 375 K.

Deuterated solvents were purchased from Sigma-Aldrich and Cambridge Isotope Laboratories. NMR spectra were recorded on Bruker Spectrospin 300/400/500 MHz spectrometers. Routine NMR assignments were confirmed by ^{1}H-^{1}H (COSY) and ^{13}C-^{1}H (HMQC) correlation experiments where necessary. The spectra were internally referenced using the residual protio solvent ($CDCl_3$, CD_3CN etc.) resonance relative to tetramethylsilane (δ = 0 ppm). ESI mass spectra were recorded on an Agilent Technologies 1260 Infinity spectrometer or a Bruker DaltonicsMicroTOF spectrometer. Infra-Red spectra were measured using a Bruker Alpha-P FTIR spectrometer. Elemental analyses were performed by Medac Ltd. Chobham, Surrey GU24, 8JB, UK.

The compounds 5-(chloromethyl)-2,2'-bipyridine,[1, 2] 5-hydroxypicolinaldehyde,[3] and phenylglycinol[4] were synthesised by reported methods.

5-(Prop-2-yn-1-yloxy)picolinaldehyde[5]

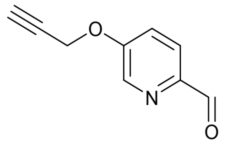

Minor improvements to the previously reported method[5] are included.

Potassium carbonate (1.45 g, 10.5 mmol) was added to a solution of 5-(hydroxy)picolinaldehyde (1.23 g, 10.0 mmol) in acetonitrile (40 ml), followed by the addition of propargyl bromide (80 wt% in toluene, 1.17 ml). The reaction mixture was then heated to reflux (*ca*. 85 °C) overnight. After cooling to ambient temperature, the reaction solution was passed through a short column of silica. The solvent was evaporated under reduced pressure and the crude product was recrystallized from dichloromethane/ n-hexane (v:v= 20:80), to yield the final product as a white solid (0.45 g, 80 % yield).^{1}H NMR (400 MHz, 298 K, C_2D_6OS) δ_H 9.90 (1H, s, HC=O), 8.54 (1H, d, $^4J_{HH}$ = 3.0 Hz), 7.98 (1H, d, $^3J_{HH}$ = 9.0 Hz), 7.64 (1H, dd, $^3J_{HH}$ = 9.0 Hz, $^4J_{HH}$ = 3.0 Hz, py), 5.05 (2H, d, $^4J_{HH}$ = 2.5 Hz, C$\underline{H_2}$–CCH), 3.71 (1H, t, $^4J_{HH}$ = 2.5Hz, C≡CH);^{13}C {^{1}H} NMR (100 MHz, 298 K, C_2D_6OS) δ_C 192.5 (CO), 157.2/ 146.7/ 139.4/ 123.9/ 122.3 (py), 80.0 (C$\underline{C}$H), 78.5 ($\underline{C}$CH), 56.8 (CH_2); IR υ(cm^{-1}) 3210 w, 1690 s, 1570 s, 1485 w, 1380 w, 1305 m, 1276 w, 1260 s, 1200 s, 1005 s, 970 m, 914 w, 830 s, 802 s, 730 m, 693 s, 661 s; HRMS Calculated for $[M+Na]^+$ *m/z* 184.0369, found *m/z* 184.0368. Elemental Analysis found (Calculated for $C_9H_7NO_2$) % C 66.75 (67.08), H 4.32 (4.38), N 8.61 (8.69).

(*S*)-2-(2,2'-bipyridin-5-ylmethoxy)-1-phenylethanamine.[6]

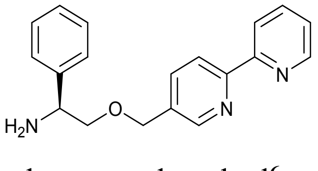

Minor improvements to the previously reported method[6] are included.

S-phenylglycinol (1.00 g, 7.3 mmol) was dissolved in dry THF (20 ml) and was added dropwise to a stirred suspension of sodium hydride (0.36 g, 15.0 mmol) in dry THF (10 ml) under inert argon atmosphere. The solution was stirred for 1 h at room temperature. A solution of 5-(chloromethyl)-2,2'-bipyridine (1.82 g, 7.3 mmol) in dry THF (20 ml) was then added dropwise and the solution was stirred for 1 h at room temperature before being heated to reflux (65 ˚C)

for a further 2 h. The reaction mixture was then cooled to ambient temperature and brine (40 ml) was added. The product was extracted with diethyl ether (4 × 60 ml), dried over sodium sulphate and the solvent was removed under reduced pressure to leave a dark yellow oil which was further purified by silica gel flash chromatography (petroleum ether/EtOAc/triethylamine, 8:8:1) to give the pure product as a white solid (1.77 g, 98% yield). R_f (petroleumether/EtOAc/trimethylamine 8:4:1) = 0.45; ^{1}H NMR (300 MHz, 298 K, $CDCl_3$) δ_H 8.72-8.65 (1H, m, Ar-H), 8.63 (1H, d, $^4J_{HH}$ = 1.0 Hz, Ar-H), 8.38 (2H, dd, $^3J_{HH}$ = 8.0 Hz, $^4J_{HH}$ = 4.0 Hz, Ar-H), 7.86-7.74 (2H, m, Ar-H), 7.43-7.23 (6H, m, Ar-H), 4.62 (2H, s, OCH_2), 4.26 (1H, dd, $^3J_{HH}$ = 8.5 Hz, $^4J_{HH}$ = 4.0 Hz, CH), 3.65 (1H, dd, $^3J_{HH}$ = 9.0 Hz, $^4J_{HH}$ = 4.0 Hz, CH_2), 3.52 (1H, t, $^3J_{HH}$ = 9.0 Hz, CH_2);^{13}C {^{1}H} NMR (75 MHz, $CDCl_3$) δ_C 156.0/ 155.7/ 149.2/ 148.6/ 142.3/ 137.0/ 136.4/ 133.7/ 128.5/ 127.5/ 126.8/ 123.7/ 121.1/ 120.8 (Ar), 77.4 (CH_2), 70.7 (CH_2), 55.6 (CH); HRMS Calculated for $[M+H]^+$ *m/z* 306.1601, found *m/z* 306.1598; IR υ (cm^{-1}): 3295 w, 3050 w, 3023 w, 2900 w, 2845 w, 1568 w, 1570 w, 1563 w, 1495 w, 1445 m, 1430 w, 1412 w, 1385 w, 1253 m, 1096 m, 1035 w, 1018 m, 988 w, 933 w. Elemental Analysis found (Calculated for $C_{19}H_{19}N_3O$) % C 74.53 (74.73), H 6.24 (6.27), N 13.57 (13.76).

(*R*)-2-(2,2'-bipyridin-5-ylmethoxy)-1-phenylethanamine.

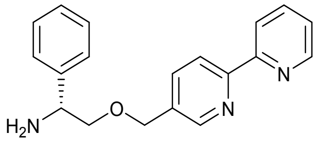

Synthesis as for (*S*)-2-(2,2'-bipyridin-5-ylmethoxy)-1-phenylethanamine above, using *R*-phenylglycinol as starting alcohol.

Spectroscopic data were as for the enantiomer above. Elemental Analysis found (Calculated for $C_{19}H_{19}N_3O$) % C 74.56 (74.73), H 6.27 (6.27), N 13.63 (13.76).

(Azidomethyl)benzene[7]

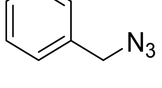

Sodium azide (1.64 g, 25.2 mmol) was added to a solution of benzyl bromide (2.0 ml, 16.8 mmol) in DMSO (25 mL). The mixture was stirred overnight at ambient temperature. Brine (75 mL) was added into the solution before extracting the product into diethyl ether (3 × 150 ml). The combined diethyl ether layers were dried over anhydrous sodium sulphate and the

solvent was removed under reduced pressure to give the product as a clear colourless oil. (1.65 g, 74%, yield).^{1}H NMR (400 MHz, 298 K, $CDCl_3$): δ_H 7.36 (5H, m, Ph), 4.34 (2H, s, CH_2); ^{13}C {^{1}H} NMR (100 MHz, 298 K, $CDCl_3$) δ_C 135.4/ 128.9/ 128.3/ 128.2 (Ph), 54.8 (CH_2); LRMS (ESI+) *m/z* 289.2 $[2M+Na]^+$, (EI+) *m/z* 133.2 $[M]^+$.

1-(Azidomethyl)-4-fluorobenzene[8]

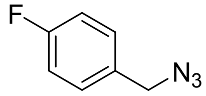

1-(Bromomethyl)-4-fluorobenzene (2.0 ml, 16.84 mmol) was dissolved in DMSO (40 ml), followed by the addition of sodium azide (1.64 g, 25.26 mmol).The reaction mixture was stirred overnight at ambient temperature. Brine (75 ml) was then added slowly (exothermic) into the solution before extracting the product into diethyl ether (3 × 150 ml). The combined diethyl ether layers were dried over sodium sulphate and the solvent was removed under reduced pressure to leave a clear colourless oil. (1.63 g, 73% yield).^{1}H NMR (300 MHz, 298 K, $CDCl_3$): δ_H 7.32 (2H, t, $^3J_{HH}$ = 6.0 Hz, Ph), 7.10(2H, t, $^3J_{HH}$ = 8.0 Hz, Ph), 4.34 (2H, s, CH_2); ^{13}C {^{1}H} NMR (75 MHz, 298 K, $CDCl_3$) δ_C 164.3/ 161.0/ 130.1/ 130.0/ 115.9/ 115.7 (Ph), 54.1 (CH_2). LRMS (ESI+) *m/z* 303.2 $[2M+H]^+$, (EI+) *m/z* 151.1 $[M]^+$.

1-(Azidomethyl)-4-methoxybenzene[9]

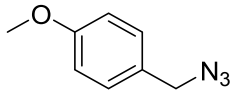

A stirred solution of *para*-substituted benzyl chloride (1.155 g, 7.38 mmol) and sodium azide (0.72 mg, 11.1 mmol) in DMF (12 mL) was gradually heated to 60 °C for 6 h. The cooled reaction mixture was partitioned between diethyl ether and water. The organic layer was then dried with magnesium sulphate and the solvent was removed under reduced pressure to yield a colourless oil. (1.12 g, 93% yield). ^{1}H NMR (300 MHz, 298 K, $CDCl_3$): δ_H 7.27 (2H, d, $^3J_{HH}$ = 7.5 Hz, Ph), 6.94 (2H, d, $^3J_{HH}$ = 7.5 Hz, Ph), 4.30 (2H, s, CH_2), 3.84 (3H, s, CH_3); ^{13}C {^{1}H} NMR (75 MHz, 298 K, $CDCl_3$) δ_C 159.7/ 129.8/ 127.4/ 114.2 (Ph), 55.3 (CH_3), 54.4 (CH_2). HRMS Calculated for $[M+Na]^+$ *m/z* 186.0638, found *m/z* 186.0636.

4-(Azidomethyl)benzonitrile[10]

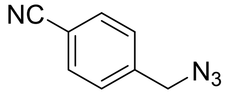

The 4-(bromomethy)benzonitrile (1.0 g, 5.1 mmol) and sodium azide (0.5 g, 7.7 mmol) were dissolved in DMF/H_2O (10mL, 2:1) and the reaction mixture was heated at 85 °C overnight. After cooling to room temperature, brine (8 mL) was then added into the solution and the product was extracted with diethyl ether (3×10 mL). The combined organic layers were dried over anhydrous sodium sulphate, filtered and concentrated under reduced pressure to give the 4-(bromomethy)benzonitrile as colourless oil (0.71 g, 87.5% yield).^{1}H NMR (400 MHz, 298 K, $CDCl_3$): δ_H 7.68 (2H, d, $^3J_{HH}$ = 7.5 Hz, Ph), 7.44 (2H, d, $^3J_{HH}$ = 7.5 Hz, Ph), 4.45 (2H, s, CH_2);^{13}C {^{1}H} NMR (100 MHz, 298 K, $CDCl_3$) δ_C 140.8/ 132.6/ 128.5 (Ph), 118.4 (CN), 112.2 (Ph), 54.1 (CH_2); HRMS Calculated for $[M+Na]^+$ *m/z* 181.0485, found *m/z* 181.0482.

4-Azidomethyl Benzoic acid[11]

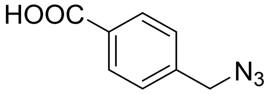

A solution of 4-chloromethyl benzoic acid (1 g, 5.86 mmol) and sodium azide (1.14 g, 17.54 mmol) in DMSO (100 mL) was heated at 80 °C for 2 days. After cooling to room temperature, brine (100 mL) was added into the solution, and the mixture was diluted with diethyl ether (300 mL). The organic phase was dried over anhydrous sodium sulphate. The solvent was removed under reduced pressure to give the pure product as a white solid (0.87 g, 84.3% yield). ^{1}H NMR (300 MHz, 298 K, $CDCl_3$): δ_H 8.16 (2H, d, $^3J_{HH}$= 7.5 Hz, Ph), 7.47 (2H, d, $^3J_{HH}$= 7.5 Hz, Ph), 4.48 (2H, s, CH_2); ^{13}C {^{1}H} NMR (75 MHz, 298 K, $CDCl_3$) δ_C 171.1 (CO), 141.5/ 130.8/ 129.1/ 128.0 (Ph), 54.3 (CH_2). HRMS Calculated for $[M-H]^-$ *m/z* 176.0466, found *m/z* 176.0464.

Synthesis of R_{c},Δ_{Zn},HHT-[$Zn_2L^2_3$][ClO_4]$_4$.

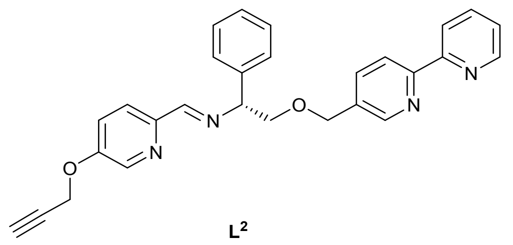

L²

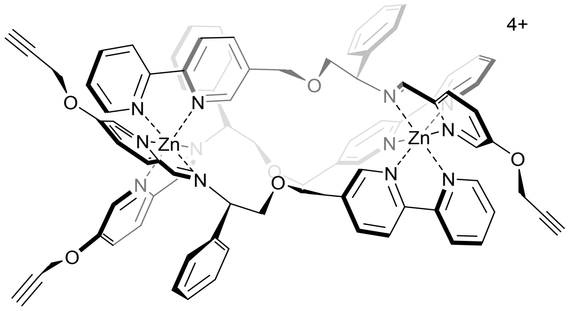

$Zn(ClO_4)_2 \cdot 6H_2O$ (0.11 g, 0.30 mmol) was added to a stirred solution of the 5-(propargyloxy)picolinaldehyde (71 mg, 0.44 mmol) and (*R*)-2-(2,2'-bipyridin-5-ylmethoxy)-1-phenylethanamine (0.135 g, 0.44 mmol) in acetonitrile (20 ml) at ambient temperature for 4 h. Ethyl acetate was then added to the resulting yellow solution to give the desired product as a yellow crystalline solid (0.23 g, 72% yield). ^{1}H NMR (500 MHz, 298 K, CD_3CN) δ_H 9.26 (1H, s, HC=N), 9.23 (1H, s, bpy), 9.17(1H, s,HC=N), 9.17 (1H, s, bpy), 8.81 (1H, s, HC=N), 8.54 (1H, d, $^3J_{HH}$ = 8.0 Hz, bpy), 8.49 (1H, d, $^3J_{HH}$ = 8.0 Hz, bpy), 8.39 (1H, s, bpy), 8.30 (1H, d, $^3J_{HH}$ = 9.0 Hz, py), 8.28-8.19 (3H, m, py/bpy), 8.13-7.90 (8H, m, bpy), 7.89-7.74 (5H, m, py/bpy), 7.71 (1H, d, $^4J_{HH}$ = 2.5 Hz, py), 7.59-7.45 (6H, m, py/bpy), 7.23-7.14 (2H, m, Ph/py), 7.11 (2H, t, $^3J_{HH}$ = 7.5 Hz, Ph), 7.03 (1H, t, $^3J_{HH}$ = 7.5 Hz, Ph), 6.97 (2H, d, $^3J_{HH}$ = 7.5 Hz, Ph), 6.91 (1H, t, $^3J_{HH}$ = 7.5 Hz, Ph), 6.72 (2H, t, $^3J_{HH}$ = 7.5 Hz, Ph), 6.58 (2H, t, $^3J_{HH}$ = 7.5 Hz, Ph), 6.11 (2H, d, $^3J_{HH}$ = 7.5 Hz, Ph), 5.99 (2H, d, $^3J_{HH}$ = 7.5 Hz, Ph), 5.48 (1H, dd, $^2J_{HH}$ = 11.5 Hz, $^3J_{HH}$ = 3.0 Hz, C$\underline{H}$Ph), 5.22 (1H, d, $^2J_{HH}$=13 Hz, OC$\underline{H}_2$-bpy) 5.21 (1H, d, $^2J_{HH}$=13 Hz, OC$\underline{H}_2$-bpy), 5.16 (1H, d, $^2J_{HH}$=13 Hz, OC$\underline{H}_2$-bpy), 4.97 (1H, dd, $^3J_{HH}$ = 11.5 Hz, $^3J_{HH}$ =3.5 Hz, C$\underline{H}$Ph), 4.88 (2H, d, $^4J_{HH}$ = 2.0 Hz, C$\underline{H}_2$-CCH), 4.84-4.78 (3H, m, C$\underline{H}_2$-CCH/C$\underline{H}$Ph), 4.75 (2H, d, $^4J_{HH}$ = 2.0 Hz, C$\underline{H}_2$-CCH), 4.54 (1H, d, $^2J_{HH}$=13 Hz, OC$\underline{H}_2$-bpy), 4.52 (1H, d, $^2J_{HH}$=13 Hz, OC$\underline{H}_2$-bpy), 4.48 (1H, d, $^2J_{HH}$=13 Hz, OC$\underline{H}_2$-bpy), 4.30 (1H, t, J_{HH} = 11.0 Hz, C$\underline{H}_2$-CHPh), 4.18 (1H, t, J_{HH} = 11.0 Hz, C$\underline{H}_2$-CHPh), 4.10 (1H, t, J_{HH} = 11.0 Hz, C$\underline{H}_2$-CHPh), 3.64 (1H, dd, $^2J_{HH}$ = 10.5 Hz, $^3J_{HH}$ = 3.5 Hz, C$\underline{H}_2$-CHPh), 3.54 (1H, dd, $^2J_{HH}$ = 10.5 Hz, $^3J_{HH}$ = 3.5 Hz, C$\underline{H}_2$-CHPh), 3.48 (1H, dd, $^2J_{HH}$ = 10.5 Hz, $^3J_{HH}$ = 3.5 Hz, C$\underline{H}_2$-CHPh), 3.03 (1H, t, $^4J_{HH}$ = 2.0 Hz, C≡CH), 2.92 (1H, t, $^4J_{HH}$ = 2.0 Hz, C≡CH), 2.83 (1H, t, $^4J_{HH}$ = 2.2 Hz, C≡CH);

^{13}C {^{1}H} NMR (125MHz, 298 K, CD_3CN) δ_C 163.2/ 162.8/ 162.3 (HC=N), 158.6/ 158.2/ 157.8 (q, py), 150.7/ 150.5/ 149.7 (bpy), 149.5/ 149.4/ 149.3 (q, bpy), 148.9(q)/ 148.6/ 148.5(q)/ 148.5/ 148.3/ 148.0 (q, bpy), 143.4/ 143.1/ 143.0 (bpy), 142.3/ 141.9/ 141.8 (bpy), 140.7/ 140.7/ 140.6 (q, py), 138.4/ 138.3/ 138.3 (py), 137.8/ 137.4/ 137.2 (q, bpy), 135.1/ 134.2/ 133.8 (q, Ph), 132.3/132.0/ 131.2 (py), 129.2/ 129.1/ 129.1/ 129.0/ 129.0/ 128.9 (Ph), 127.8/ 127.6/ 127.4

(bpy), 127.2/ 126.5/ 126.4 (Ph), 125.5/ 125.5/ 125.3 (py), 124.0/ 123.8/ 123.6/ 123.5/ 123.0/ 122.9 (bpy), 78.4/ 78.4/ 78.3 (C≡C̲H), 77.1/ 76.9/ 76.9 (q, C̲≡CH), 70.1/ 70.0 (C̲H_2-bpy), 69.5 (C̲HPh), 69.4 (C̲H_2-bpy), 69.3 (C̲HPh), 69.3/ 69.0/ 68.9 (C̲H_2-CHPh), 67.5 (C̲HPh), 57.3/ 57.2/ 57.1 (C̲H_2-C≡CH); LRMS (ESI+) *m/z* 449.3 $[L+H]^+$, 471.3 $[L+Na]^+$, 478.4 $[L+K]^+$, HRMS Calculated for $[L+H]^+$ *m/z* 449.1972, found *m/z* 449.1973.

Elemental Analysis found (Calculated for $C_{84}H_{72}Cl_4N_{12}O_{22}Zn_2{\cdot}4H_2O$) % C 52.23 (51.84), H 3.72 (4.14), N 8.53 (8.64).

IR *v* cm^{-1} 3568 (br, m), 1572 (m), 1475 (w), 1440 (w), 1220 (m), 1077 (s), 1008 (s), 932 (m), 860 (w), 752 (w), 698 (w), 620 (s).

General procedure for the synthesis of aromatic clicked complexes R_c,Δ_{Zn},HHT-$[Zn_2L^n{}_3][ClO_4]_4$(where n =3a-3d)

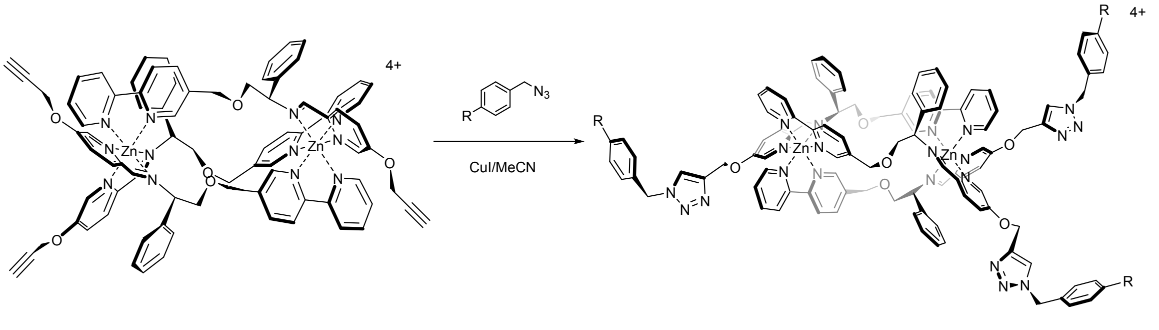

R_c,Δ_{Zn},HHT-$[Zn_2\mathbf{L^{3a}}_3]$ (R = H)
-$[Zn_2\mathbf{L^{3b}}_3]$ (R = F)
-$[Zn_2\mathbf{L^{3c}}_3]$ (R =CN)
-$[Zn_2\mathbf{L^{3d}}_3]$ (R =OCH_3)

The benzyl azide derivate (4.5 equiv.) and R_c,Δ_{Zn},HHT-$[Zn_2L^2{}_3][ClO_4]_4$ (1.0 equiv.) were dissolved in acetonitrile (10 ml), followed by the addition of copper(I) iodide (0.1 equiv.). The reaction mixture was heated at 65˚C for 18 h under inert argon atmosphere. After cooling to ambient temperature, the suspension was filtered to remove copper salts and the resulting pale yellow solution yielded the desired product as a yellow crystalline solid upon the addition of ethyl acetate.

R_c,Δ_{Zn},HHT-[$Zn_2L^{3a}_3$][ClO_4]$_4$

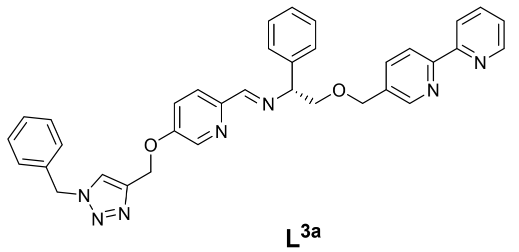

L^{3a}

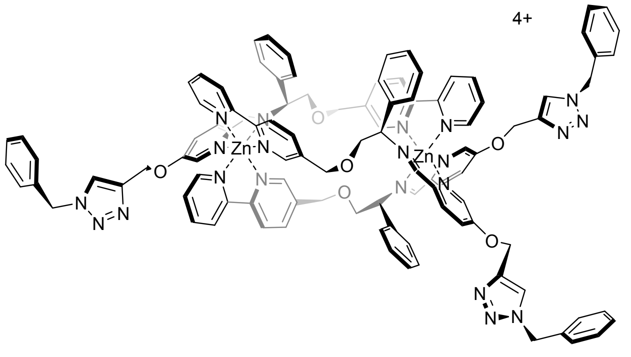

Yield 0.18 g, 82%.

^{1}H NMR (500 MHz, 298 K, CD_3CN) δ_H 9.25 (1H, s, HC=N), 9.20 (1H, s, bpy), 9.14 (1H, s, bpy), 9.11 (1H, s, HC=N), 8.75 (1H, s, HC=N), 8.52 (1H, d,$^3J_{HH}$ = 8.0, bpy), 8.48 (1H, d,$^3J_{HH}$ = 8.0, bpy), 8.36 (1H, s, bpy), 8.28 (1H, d,$^3J_{HH}$ = 9.0 Hz, py), 8.24-7.71 (23H, m, Ph/bpy/py/TRZ), 7.59 (1H, d,$^4J_{HH}$ = 2.5 Hz, py), 7.57-7.23 (27H, m, Ph/bpy/py), 7.10-6.87 (5H, m, Ph/py), 6.82 (1H, d,$^3J_{HH}$ = 8.0 Hz, Ph) 6.70 (2H, t, $^3J_{HH}$ = 8.0 Hz, Ph), 6.56 (2H, t, $^3J_{HH}$ = 8.0 Hz, Ph), 6.09 (2H, d, $^3J_{HH}$ = 8.0 Hz, Ph), 5.96 (2H, d, $^3J_{HH}$ = 8.0 Hz, Ph), 5.60 (2H, s, $PhCH_2TRZ$), 5.57 (2H, s, $PhCH_2TRZ$), 5.48 (2H, s, $PhCH_2TRZ$), 5.42 (1H, dd, $^2J_{HH}$= 11.0Hz, $^3J_{HH}$= 3.0, $C\underline{H}Ph$), 5.32-5.09 (10H, m, TRZ-CH_2O/$OC\underline{H}_2$-bpy), 4.94 (1H, dd, $^2J_{HH}$ = 11.0Hz, $^3J_{HH}$ = 3.0, $C\underline{H}Ph$), 4.80 (1H, dd, $^2J_{HH}$ = 11.0Hz, $^3J_{HH}$ = 3.0, $C\underline{H}Ph$), 4.53 (2H, d, $^2J_{HH}$= 13.0 Hz, $OC\underline{H}_2$-bpy), 4.46 (1H, d, $^3J_{HH}$ = 13.0 Hz, $OC\underline{H}_2$-bpy), 4.29 (1H, t, J_{HH} = 11.0 Hz, $C\underline{H}_2$-CHPh), 4.15 (1H, t, J_{HH} = 11.0 Hz, $C\underline{H}_2$-CHPh), 4.07 (1H, t, J_{HH} = 11.0 Hz, $C\underline{H}_2$-CHPh), 3.60 (1H, dd, $^2J_{HH}$= 10.0Hz,$^3J_{HH}$= 3.5, $C\underline{H}_2$-CHPh), 3.52 (1H, dd, $^2J_{HH}$= 11.0 Hz,$^3J_{HH}$= 3.0, $C\underline{H}_2$-CHPh), 3.45 (1H, dd, $^2J_{HH}$= 11.0Hz, $^3J_{HH}$= 3.5, $C\underline{H}_2$-CHPh).

^{13}C {^{1}H} NMR (125 MHz, 298 K, CD_3CN) δ_C 163.3/ 163.0/ 162.3(HC=N), 159.6/ 159.3/ 158.7 (q, py), 150.8/ 150.6/ 149.8 (bpy), 149.7/ 149.6/ 149.4/ 149.2 (q, bpy), 148.7/ 148.3 (bpy), 148.1 (q, bpy), 143.5/ 143.1/ 143.1 (q, bpy), 142.5/ 142.3 (q, TRZ), 142.0/, 141.7 (bpy), 140.4/ 140.3 (q, py), 138.9/ 138.7/138.4 (py) 137.8/ 137.5/137.2 (q, bpy), 136.2/ 136.1 (q, $\underline{Ph}CH_2$), 135.3/ 134.4/ 134.0 (q, Ph), 132.5/ 132.3/131.3 (py), 129.6/ 129.5/129.5/ 129.3/ 129.2/ 129.1/ 129.1/ 129.0/ 129.0/128.8/ 128.7, 128.6 (Ph/bpy),127.2/ 126.6/126.5 (bpy)/ 126.1 (py), 125.3/ 125.4/ 125.1 (TRZ), 124.3/ 124.0/ 123.8/ 123.7/ 123.1/ 123.1 (bpy), 70.2/ 70.2/ 69.6 ($\underline{C}H_2$-bpy), 69.6 ($\underline{C}HPh$), 69.4 ($\underline{C}H_2$-bpy), 69.4 ($\underline{C}HPh$), 69.1/ 69.0 ($\underline{C}H_2$-CHPh), 67.5 ($\underline{C}HPh$), 62.8/ 62.7 ($TRZ\underline{CH_2}O$),54.2/ 54.1/ 54.0 (Ph-$\underline{C}H_2$-TRZ).

Elemental Analysis found (Calculated for $C_{105}H_{93}Cl_4N_{21}O_{22}Zn_2 \cdot 16H_2O$) % C 49.03 (49.23), H 3.63 (4.92), N 11.37 (11.48).

LRMS (ESI+) *m/z* 582.4 $[L+H]^+$, 604.3 $[L+Na]^+$, HRMS Calculated for $[L+Na]^+$ *m/z* 604.2431, found *m/z* 604.2424.

IR ν cm^{-1} 3519 (br, m), 3039 (br, m), 1570 (m), 1216 (m), 1076 (s), 933 (w), 794 (w), 752 (w), 697 (w), 621 (m).

R_c,Δ_{Zn},HHT-$[Zn_2L^{3b}_3][ClO_4]_4$.

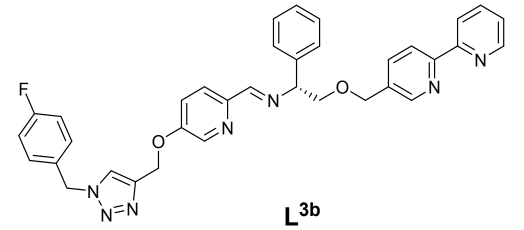

L^{3b}

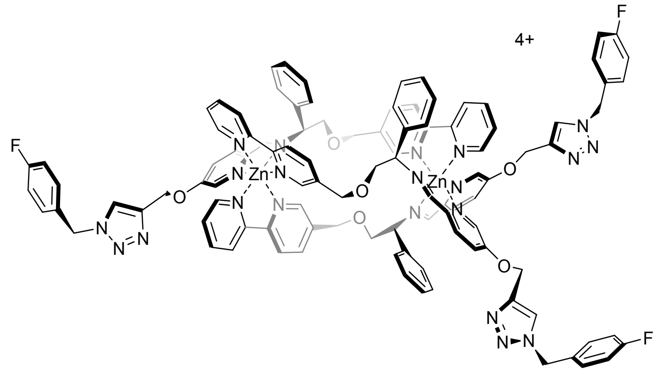

Yield 0.23 g, 66 %.

^1H NMR (500 MHz, 298 K, CD_3CN) δ_H 9.24 (1H, s, HC=N), 9.20 (1H, s, bpy), 9.14 (1H, s, bpy), 9.10 (1H, s, HC=N), 8.74 (1H, s, HC=N), 8.52 (1H, d,$^3J_{HH}$ = 8.0 hZ, bpy), 8.49 (1H, d, $^3J_{HH}$ = 8.0 hZ, bpy), 8.36 (1H, s, bpy), 8.28 (1H, d, $^3J_{HH}$ = 9.0 Hz, py), 8.23-8.17 (4H, m, bpy/py), 8.08-7.74 (20H, m, Ph/py/TRZ), 7.62-7.26 (18H, m, Ph/py/bpy), 7.24-6.99 (11H, m, Ph/py/bpy),6.94-6.85 (3H, m, Ph/bpy), 6.78 (2H, d, $^3J_{HH}$ = 8.0 Hz, Ph), 6.71 (2H, t, $^3J_{HH}$ = 8.0 Hz, Ph), 6.56 (2H, t, $^3J_{HH}$ = 8.0 Hz, Ph), 6.09 (2H, d, $^3J_{HH}$ = 8.0 Hz, Ph), 5.96 (2H, d, $^3J_{HH}$ = 8.0 Hz, Ph), 5.59 (2H, s, $PhCH_2TRZ$), 5.55 (2H, s, $PhCH_2TRZ$), 5.46 (2H, s, $PhCH_2TRZ$), 5.42 (1H, dd, $^2J_{HH}$ = 11.0 Hz, $^3J_{HH}$ = 3.0, C<u>H</u>Ph), 5.31-5.08 (10H, m, TRZ-CH_2O/OC<u>H_2</u>-bpy), 4.94 (1H, dd, $^2J_{HH}$ = 11.0 Hz, $^3J_{HH}$ = 3.0, C<u>H</u>Ph), 4.78(1H, dd, $^2J_{HH}$ = 11.0 Hz, $^3J_{HH}$ = 3.0, C<u>H</u>Ph), 4.53 (4H, d, $^2J_{HH}$ = 13.0 Hz,OC<u>H_2</u>-bpy), 4.45 (2H, d, $^2J_{HH}$ = 13.0 Hz, OC<u>H_2</u>-bpy), 4.29 (1H, t, J_{HH} = 11.0 Hz, C<u>H_2</u>-CHPh), 4.14 (1H, t, J_{HH} = 11.0 Hz, C<u>H_2</u>-CHPh), 4.06 (1H, t, J_{HH} = 11.0 Hz, C<u>H_2</u>-CHPh), 3.59 (1H, dd, $^2J_{HH}$ = 10.0 Hz, $^3J_{HH}$ = 3.5, C<u>H_2</u>-CHPh), 3.52 (1H, dd, $^2J_{HH}$ = 11.0 Hz, $^3J_{HH}$ = 3.0, C<u>H_2</u>-CHPh), 3.45 (1H, dd, $^2J_{HH}$ = 11.0 Hz, $^3J_{HH}$ = 3.5, C<u>H_2</u>-CHPh).

^{13}C {^1H} NMR (125MHz, 298 K, CD_3CN) δ_C 163.6/ 163.5 (q, F-<u>Ph</u>), 162.7/ 162.4/ 161.7 (HC=N), 161.1 (q, F-<u>Ph</u>), 159.0/ 158.7/ 158.1 (q, py), 150.2/ 150.0/ 149.2 (bpy), 149.1/ 148.9/ 148.6 (q, bpy), 148.1/ 147.7 (bpy), 147.6 (q, bpy), 142.9/ 142.6/ 142.5 (bpy), 141.9/ 141.72 (q,

TRZ), 141.70/, 141.4/ 141.1 (bpy), 139.8/ 139.7 (q, py), 138.4/ 138.1/ 137.8 (py), 137.3/ 136.9/ 136.6 (q, PhCH_2), 134.6/ 133.8/ 133.4 (q, Ph), 131.9/, 131.8/ (py), 130.7/ 130.6/ 130.4/ 130.3/ 130.2/ 128.8/ 128.6/ 128.5/ 128.4/ 128.3 (Ph/bpy), 127.3/ 127.2/ 126.9 (bpy), 126.6/ 126.1/ 126.0 (py), 125.6/ 124.9/ 124.8 (TRZ), 124.7/ 124.5/ 124.4 (bpy), 123.7/ 123.4/ 123.2/ 122.6/ 122.5 (bpy), 155.8/ 155.7/ 155.6/ 155.5 (F-Ph), 69.6/ 69.5 (CH_2-bpy), 69.0/ 68.8 (CHPh), 68.5/ 68.4 (CH_2-CHPh), 66.9 (CHPh), 62.2/ 62.0 (TRZCH_2O), 52.8/ 52.7/ 52.6 (F-Ph-CH_2-TRZ).

Elemental Analysis found (Calculated for $C_{105}H_{90}Cl_4F_3N_{21}O_{22}Zn_2\cdot 14H_2O$) % C 49.02 (48.89), H 3.53 (4.61), N 11.39 (11.40).

LRMS (ESI+) *m/z* 600.4 $[L+H]^+$, 622.3 $[L+Na]^+$, HRMS Calculated for $[L+H]^+$ *m/z* 600.2518, found *m/z* 600.2515.

IR ν cm^{-1} 1602 (w), 1570 (w), 1315 (w), 1218 (m), 1076 (s), 841 (m), 788 (m), 751 (m), 698 (m), 620 (s).

R_c,Δ_{Zn},HHT-$[Zn_2L^{3c}{}_3][ClO_4]_4$.

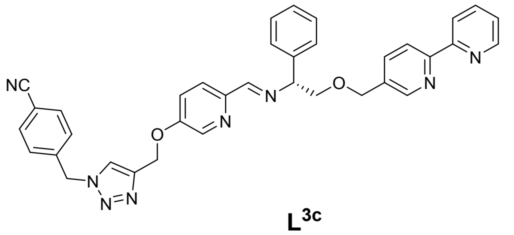

L^{3c}

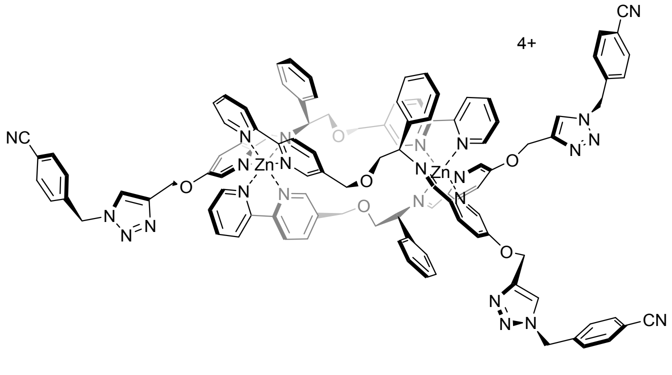

Yield 0.19 g, 74%.

^{1}H NMR (500 MHz, 298 K, CD_3CN) δ_H 9.25 (1H, s, HC=N), 9.21 (1H, s, bpy), 9.14 (1H, s, bpy), 9.10 (1H, s, HC=N), 8.73 (1H, s, HC=N), 8.52 (1H, d, $^3J_{HH}$ = 8.0 Hz, bpy), 8.48 (1H, d, $^3J_{HH}$ = 8.0 Hz, bpy), 8.36 (1H, s, bpy), 8.28 (1H, d,$^3J_{HH}$ = 9.0 Hz, py), 8.24-7.23 (54H, m, Ph/py/bpy/TRZ), 7.02 (2H, t, $^3J_{HH}$ = 7.0 Hz, Ph), 6.91 (2H, t, $^3J_{HH}$ = 7.5 Hz, Ph), 6.85 (2H, d, $^3J_{HH}$ = 8.0 Hz, Ph), 6.76 (2H, d, $^3J_{HH}$ = 8.0 Hz, Ph), 6.71 (2H, t, $^3J_{HH}$ = 8.0 Hz, Ph), 6.57 (2H, t, $^3J_{HH}$ = 8.0 Hz, Ph), 6.06 (2H, d, $^3J_{HH}$ = 8.0 Hz, Ph), 5.96 (2H, d, $^3J_{HH}$ = 8.0 Hz, Ph),5.68 (2H, s, PhCH_2TRZ), 5.65 (2H, s, PhCH_2TRZ), 5.57 (2H, s, PhCH_2TRZ), 5.41(1H, dd, $^2J_{HH}$ = 11.0 Hz,$^3J_{HH}$ = 3.0, CHPh), 5.36-5.08 (10H, m, TRZ-CH_2O/OCH_2-bpy), 4.94 (1H, dd, $^2J_{HH}$ = 11.0 Hz,$^3J_{HH}$ = 3.0, CHPh), 4.80 (1H, dd, $^2J_{HH}$ = 11.0 Hz, $^3J_{HH}$ = 3.0, CHPh), 4.53 (1H, d, $^2J_{HH}$ =

13.0 Hz, OC$\underline{H}_2$-bpy), 4.52 (1H, d, $^2J_{HH}$ = 13.0 Hz, OC$\underline{H}_2$-bpy), 4.45 (1H, d, $^2J_{HH}$ = 13.0 Hz, OC$\underline{H}_2$-bpy), 4.28 (1H, t, J_{HH} = 11.0 Hz, C$\underline{H}_2$-CHPh), 4.13 (1H, t, J_{HH} = 11.0 Hz, C$\underline{H}_2$-CHPh), 4.06 (1H, t, J_{HH} = 11.0 Hz, C$\underline{H}_2$-CHPh), 3.60 (1H, dd, $^2J_{HH}$ = 10.0 Hz, $^3J_{HH}$ = 3.5, C$\underline{H}_2$-CHPh), 3.52 (1H, dd, $^2J_{HH}$ = 11.0 Hz, $^3J_{HH}$ = 3.0, C$\underline{H}_2$-CHPh), 3.45 (1H, dd, $^2J_{HH}$ = 11.0 Hz, $^3J_{HH}$ = 3.5, C$\underline{H}_2$-CHPh).

^{13}C {^{1}H} NMR (125 MHz, CD_3CN) δ_C 162.7/ 162.4/ 161.7 (HC=N), 159.0/ 158.8/ 158.1 (q, py), 150.3/ 150.0/ 149.3 (bpy), 149.1/ 148.9/ 148.6 (q, bpy), 148.1/ 147.7 (bpy), 147.6 (q, bpy), 142.9/ 142.6/ 142.5 (bpy), 142.1/ 141.9 (q, TRZ), 141.7/ 141.4/ 141.2 (bpy), 140.9/ 140.8/ 140.7 (q, $\underline{Ph}$CN), 139.8/ 139.7 (q, py) 138.4/ 138.2/ 137.7 (py), 137.3/ 137.0/ 136.7 (q, $\underline{Ph}CH_2$), 134.6/ 133.9/ 133.5 (q, Ph), 132.9/ 132.8 ($\underline{Ph}$CN), 131.9/ 131.8/ 130.7 (py), 129.1/ 128.8/ 128.7/ 128.6/ 128.5/ 128.4/ 128.3 (Ph/bpy), 127.3/ 127.2/ 126.9 (bpy), 126.6/ 126.1/ 126.0 (py), 125.7/ 125.2/ 125.1/ 125.0/ 124.9/ 124.5/ 123.7/ 123.5/ 123.2/ 122.6/ 122.5 (bpy), 118.3 (CN), 112.0 (q, $\underline{Ph}$CN), 69.7/ 69.6 ($\underline{C}H_2$-bpy), 69.03 ($\underline{C}$HPh), 69.00 ($\underline{C}H_2$-bpy), 68.9 ($\underline{C}H_2$-CHPh), 68.8 ($\underline{C}$HPh), 68.6/ 68.5 ($\underline{C}H_2$-CHPh), 67.0 ($\underline{C}$HPh), 62.2/ 62.0/ 61.9 (TRZ$\underline{CH_2}$O), 53.0/ 52.9/ 52.8 (CNPh-$\underline{C}H_2$-TRZ).

Elemental Analysis found (Calculated for $C_{108}H_{90}Cl_4N_{24}O_{22}Zn_2\cdot 16H_2O$) % C 49.57 (49.19), H 3.36 (4.66), N 12.84 (12.75).

LRMS (ESI+) *m/z* 607.3 $[L+H]^+$, HRMS Calculated for $[L+H]^+$ *m/z* 607.2564, found *m/z* 607.2559.

IR *v* cm^{-1} 2229 (w), 1571 (m), 1475 (w), 1440 (w), 1317 (w), 1263 (w), 1218 (w), 1080 (s), 827 (m), 792 (m), 753 (m), 698 (m), 622 (s).

R_c,Δ_{Zn},HHT-$[Zn_2L^{3d}_3][ClO_4]_4$.

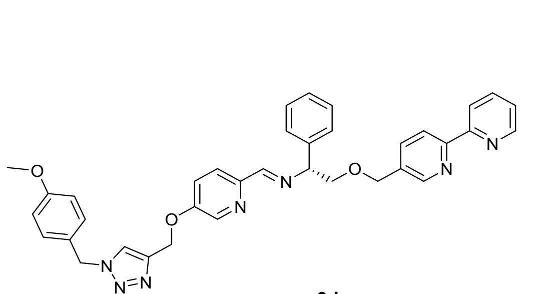

L^{3d}

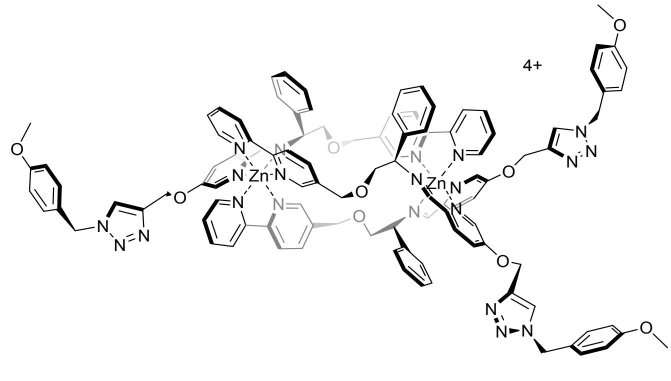

Yield 0.26 g, 90%.

^{1}H NMR (500 MHz, 298 K, CD_3CN) δ_H 9.22 (1H, s, HC=N), 9.17 (1H, s, bpy), 9.11 (1H, s, bpy), 9.06 (1H, s, HC=N), 8.71 (1H, s, HC=N), 8.51 (1H, d,$^3J_{HH}$ = 8.0 Hz, bpy), 8.48 (1H, d,$^3J_{HH}$ = 8.0 Hz, bpy), 8.34 (1H, s, bpy), 8.25 (1H, d, $^3J_{HH}$ = 9.0Hz, py), 8.23-8.15 (4H, m, py), 8.08-7.71 (20H, m, Ph/py/TRZ), 7.56 (1H, d, $^4J_{HH}$ = 2.5, py), 7.52-7.45 (3H, m, Ph/py), 7.42-7.25 (10H, m, Ph/bpy/py), 7.20 (2H, d, $^3J_{HH}$ = 9.0 Hz, py), 7.06-6.83 (14H, m, Ph/py), 6.74 (2H, d, $^3J_{HH}$ = 8.0 Hz, Ph), 6.68 (2H, t, $^3J_{HH}$ = 8.0 Hz, Ph), 6.53 (2H, t, $^3J_{HH}$ = 8.0 Hz, Ph), 6.06 (2H, d, $^3J_{HH}$ = 8.0 Hz, Ph), 5.93 (2H, d, $^3J_{HH}$ = 8.0 Hz, Ph), 5.49 (2H, s, $PhCH_2TRZ$), 5.46 (2H, s, $PhCH_2TRZ$), 5.43-5.37 (3H, m, $PhCH_2TRZ$/ CHPh), 5.26-5.06 (10H, m, TRZ-CH_2O/$OC\underline{H}_2$-bpy), 4.91 (1H, dd, $^2J_{HH}$ = 11.0Hz, $^3J_{HH}$ = 3.0, C$\underline{H}$Ph), 4.77 (1H, dd, $^2J_{HH}$ = 11.0Hz, $^3J_{HH}$ = 3.0, C$\underline{H}$Ph), 4.52 (2H, d, $^2J_{HH}$= 13.0 Hz, $OC\underline{H}_2$-bpy), 4.45 (2H, d, $^2J_{HH}$= 13.0 Hz, $OC\underline{H}_2$-bpy)), 4.26 (1H, t, J_{HH} = 11.0 Hz, $C\underline{H}_2$-CHPh), 4.11 (1H, t, J_{HH} = 11.0 Hz, $C\underline{H}_2$-CHPh), 4.03 (1H, t, J_{HH} = 11.0 Hz, $C\underline{H}_2$-CHPh), 3.78 (3H, s, OCH_3), 3.76 (3H, s, OCH_3),3.74 (3H, s, OCH_3), 3.56 (1H, dd, $^2J_{HH}$= 10.0 Hz, $^3J_{HH}$= 3.5, $C\underline{H}_2$-CHPh), 3.49 (1H, dd, $^2J_{HH}$= 11.0 Hz, $^3J_{HH}$= 3.0, $C\underline{H}_2$-CHPh), 3.42 (1H, dd, $^2J_{HH}$= 11.0 Hz, $^3J_{HH}$= 3.5, $C\underline{H}_2$-CHPh).

^{13}C {^{1}H} NMR (125 MHz, 298 K, CD_3CN) δ_C 162.8/ 162.4/ 161.8 (HC=N), 159.9 (q, $\underline{Ph}OCH_3$), 159.1/ 158.8/ 158.2 (q, py), 150.3/ 150.0/ 149.3 (bpy), 149.1/ 148.9/ 148.7 (q, bpy), 148.1/ 147.8 (bpy), 147.6 (q, bpy), 143.0/ 142.6/ 142.5 (bpy), 141.9 (q, TRZ), 141.8 (bpy), 141.7 (q, TRZ), 141.5/ 141.2 (bpy), 139.8/ 139.7 (q, py), 138.4/ 138.2/ 137.7 (py), 137.3/ 137.0/ 136.7 (q, bpy), 134.7/ 133.9/ 133.5 (q, Ph), 131.9/ 131.8/ 130.8 (py), 130.0/ 129.8/ 129.7 ($\underline{Ph}OCH_3$) 128.8/ 128.7/ 128.6/ 128.5/ 128.4 (Ph), 127.6/ 127.5 ($\underline{Ph}OCH_3$), 127.3/ 127.2/ 126.9 (bpy), 126.7/ 126.1/ 126.0 (Ph), 125.8/ 125.0/ 124.6 (py), 124.5/ 124.3 (TRZ), 123.8/ 123.5/ 123.3/ 123.2/ 122.6/ 122.5 (bpy), 114.4/ 114.3/ 114.2 ($\underline{Ph}OCH_3$), 69.7/ 69.6/ 69.1 ($\underline{C}H_2$-bpy), 69.0 ($\underline{C}$HPh), 68.9 ($\underline{C}H_2$-CHPh), 68.8 ($\underline{C}$HPh), 68.6/ 68.5 ($\underline{C}H_2$-CHPh), 67.0 ($\underline{C}$HPh), 62.3/ 62.1 (TRZ$\underline{CH_2}$O), 55.1 (OCH_3), 53.3/ 53.2/ 53.1 (CH_3OPh-$\underline{C}H_2$-TRZ).

Elemental Analysis found (Calculated for $C_{108}H_{99}Cl_4N_{21}O_{25}Zn_2\cdot 14H_2O$) % C 49.44 (49.59), H 3.74 (4.89), N 11.10 (11.24).

LRMS (ESI+) *m/z* 612.4 $[L+H]^+$, HRMS Calculated for $[L+H]^+$ *m/z* 612.2718, found *m/z* 612.2714.

IR ν cm^{-1} 1571 (m), 1514 (w), 1316 (w), 1248 (m), 1079 (s), 839 (w), 791 (m), 752 (m), 698 (w), 621 (s).

Synthesis of Δ_{Fe},HHT-[$Fe_2L^2{}_3$]Cl_4

Anhydrous $FeCl_2$ (0.10 g, 0.82 mmol) was added to a stirred solution of the 5-(prop-2-yn-1-yloxy)picolinaldehyde (0.20 g, 1.24 mmol) and (*R*)-2-(2,2'-bipyridin-5-ylmethoxy)-1-phenylethanamine (0.38 g, 1.24 mmol) in methanol (50 ml) at ambient temperature to give a purple solution that was then heated to reflux for 48 h. The reaction mixture was cooled to room temperature, filtered through a celite plug prior to the solvents being removed *in vaccuo* to yield the desired product as a purple solid.

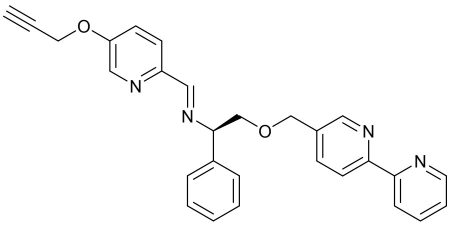

L^2

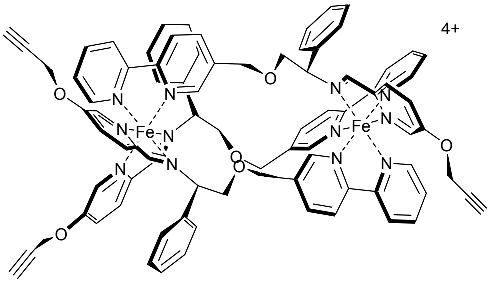

Yield 0.61 g, 90%.

^{1} H NMR (500 MHz, 298 K, D_2O) δ_H 9.57 (1H, s, HC=N), 9.48 (1H, s, HC=N), 9.16 (1H, s, bpy),9.15 (1H, s, bpy), 9.06 (1H, s, HC=N), 8.44 (2H, d, $^3J_{HH}$ = 10.0 Hz, bpy), 8.37 (1H, d, $^3J_{HH}$ = 10.0 Hz, py), 8.25 (1H, d, $^3J_{HH}$ = 10.0 Hz, py), 8.04-7.98 (2H, m, bpy), 7.89 (1H, d, $^3J_{HH}$ = 5.0 Hz, bpy), 7.86-7.62 (9H, m, Ph/bpy/py), 7.54 (1H, s, bpy), 7.50 (1H, d, $^3J_{HH}$ = 10.0 Hz, py), 7.35-6.84 (15H, m, Ph/py/bpy), 6.65 (2H, t, $^3J_{HH}$=7.5, Ph), 6.54 (2H, t,$^3J_{HH}$ =7.5 Hz, Ph), 6.41(1H, d, $^4J_{HH}$=0.4 Hz, py), 5.77 (2H, s, Ph), 5.32 (1H, dd, $^2J_{HH}$ = 11.0Hz,$^3J_{HH}$ = 3.5, C$\underline{H}$Ph), 5.21 (1H, d, $^2J_{HH}$= 15.0 Hz, OC$\underline{H}_2$-bpy), 5.19 (1H, d, $^2J_{HH}$= 15.0 Hz, OC$\underline{H}_2$-bpy), 4.96 (2H, d, $^2J_{HH}$= 15.0 Hz, OC$\underline{H}_2$-bpy), 4.73-4.60 (4H, m, C$\underline{H}_2$-CCH overlapping with D_2O), 4.55-4.51 (4H, m, C$\underline{H}_2$-CCH/CHPh), 4.45-4.38 (3H, m, OC$\underline{H}_2$-bpy/C$\underline{H}_2$-CHPh), 4.45-4.38 (2H, m, OC$\underline{H}_2$-bpy/ C$\underline{H}_2$-CHPh), 4.21 (1H, t, $^3J_{HH}$ =10.0 Hz, C$\underline{H}_2$-CHPh), 3.54 (1H, dd, $^2J_{HH}$ = 10.5 Hz, $^3J_{HH}$ = 3.5 Hz, C$\underline{H}_2$-CHPh), 3.35 (1H, dd, $^2J_{HH}$ = 10.5 Hz, $^3J_{HH}$ = 3.5 Hz, C$\underline{H}_2$-CHPh), 3.25 (1H, dd, $^2J_{HH}$ = 10.5 Hz, $^3J_{HH}$ = 3.5 Hz, C$\underline{H}_2$-CHPh), 3.05 (1H, s, CH), 2.84 (1H, s, CH), 2.79 (1H, s, CH).

^{13}C {^{1}H} NMR (125 MHz, 298 K, D_2O) δ_C 170.4/ 170.0/ 169.5 (HC=N), 159.7/ 158.9/ 158.6/ 158.3/ 157.9/ 157.8 (q, bpy)/ 157.5/ 157.4 (bpy), 156.0/ 155.9/ 155.6 (q, py), 154.5/ 153.8/ 153.4/ 153.3 (bpy), 152.3/ 152.0/ 151.8 (q, py), 142.6/ 141.8/ 141.7 (py), 140.0/ 139.7/ 139.6/ 138.9/ 138.6/ 138.6 (bpy), 136.9/ 136.7/ 136.3 (q, bpy), 134.1/ 132.5/ 132.2 (q, Ph), 131.1/ 130.7/ 130.2 (py), 129.1/ 129.0/ 128.9/ 128.8/ 128.7/ 128.6 (Ph), 127.3/ 127.2/ 127.1 (bpy), 124.3 (py), 123. 9/ 123.8/ 123.7/ 123.6/ 123.5 (py/bpy), 122.7/ 122.5/ 121.9 (bpy), 78.4/ 78.3/

78.2 (C≡C̲H), 77.0/ 76.8/ 76.7 (q, C̲≡CH), 72.6/ 72.5/ 70.4 (C̲HPh), 69.2/ 69.1/ 68.7 (C̲H_2-bpy), 68.5/ 68.4/ 67.9 (C̲H_2-CHPh), 56.5/ 56.4 (C̲H_2-C≡CH).

HRMS Calculated for $[Fe_2L_3]^{4+}$ m/z 364.1095, found m/z 364.1103

Elemental Analysis found (Calculated for $C_{84}H_{72}Cl_4Fe_2N_{12}O_6 \cdot 11H_2O$) % C 56.41 (56.14), H 4.68 (5.27), N 9.31 (9.35).

IR *v* cm^{-1} 3366 (br, m), 1606 (m), 1590 (m), 1557 (s), 1467 (m), 1403 (w), 1363 (m), 1277 (m), 1227 (s), 1109 (m), 1074 (s), 1002 (s), 933 (m), 842 (m), 791 (m), 754 (m), 697 (s).

Λ_{Fe},HHT-$[Fe_2L^2_3]Cl_4$

Data as for *R*-enantiomer

Yield 0.58 g, 85%.

Elemental Analysis found (Calculated for $C_{84}H_{72}Cl_4Fe_2N_{12}O_6 \cdot 11H_2O$) % C 56.31 (56.14), H 4.67 (5.27), N 9.28 (9.35).

General synthesis of clicked HHT-[Fe_2L^n]Cl_4(where n =3a-3e).

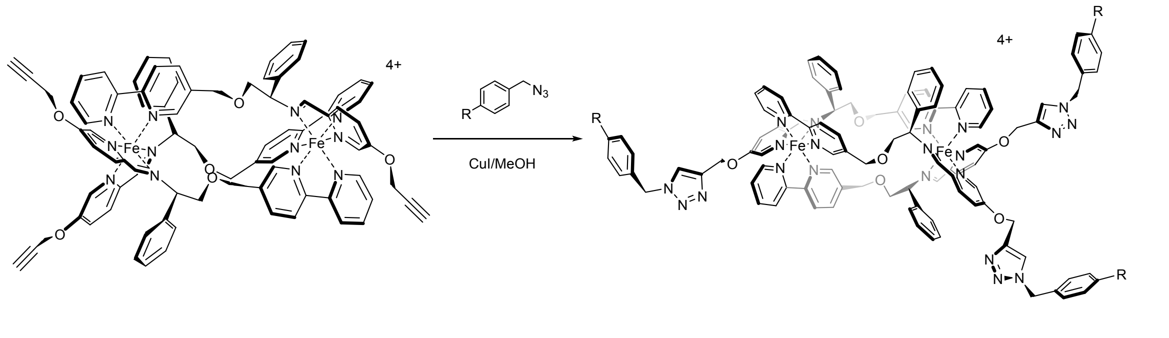

R_c,Δ_{Fe},HHT-[$Fe_2\mathbf{L^{3a}}_3$] (R = H)
-[$Fe_2\mathbf{L^{3b}}_3$] (R = F)
-[$Fe_2\mathbf{L^{3c}}_3$] (R =CN)
-[$Fe_2\mathbf{L^{3d}}_3$] (R =OCH_3)
-[$Fe_2\mathbf{L^{3e}}_3$] (R =COOH)

S_c,Λ_{Fe},HHT-[$Fe_2\mathbf{L^{3a}}_3$] (R = H)
-[$Fe_2\mathbf{L^{3b}}_3$] (R = F)
-[$Fe_2\mathbf{L^{3c}}_3$] (R =CN)
-[$Fe_2\mathbf{L^{3d}}_3$] (R =OCH_3)
-[$Fe_2\mathbf{L^{3e}}_3$] (R =COOH)

The benzyl azide derivate (4.5 equiv.) and [$Fe_2\mathbf{L^2}_3$]Cl_4 (1 equiv.) were dissolved in methanol (10 ml), followed by the addition of copper(I) iodide (0.1 equiv.). The reaction mixture was heated at 65 ˚C for 18 h under inert argon atmosphere. The suspension was filtered to remove copper salts. The resulting purple solution yielded the desired product as a purple solid on the addition of ethyl acetate.

***S*c,ΛFe,HHT-[$Fe_2L^{3a}_3$]Cl_4.**

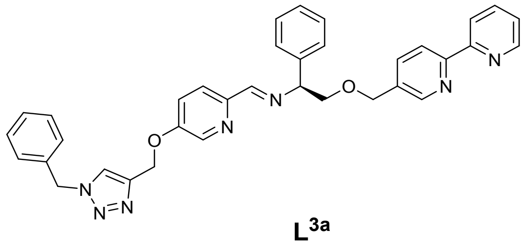

$\mathbf{L^{3a}}$

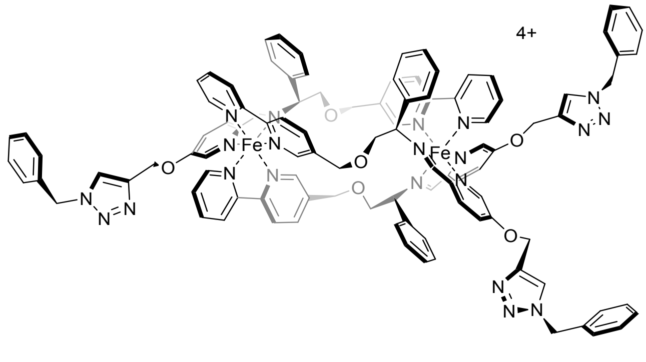

Yield 0.19 g, 82%.

^{13}C {^1H} NMR (125 MHz, 298K, CD_3OD) δ_C 171.2/ 171.1/ 170.3 (HC=N), 159.8/ 159.2/ 158.9/ 158.8/ 158.3/ 158.2 (q, bpy), 157.54 (bpy), 157.51 (q, py), 157.4 (bpy), 157.3/ 156.8 (q, py), 155.0 (bpy), 154.1/ 153.5/ 152.9 (py), 151.9/ 151.7/ 151.6 (q, py), 143.2/ 142.6/ 142.2 (py), 134.0/ 139.9/ 139.7/ 138.8/ 138.5/ 138.2 (bpy), 137.3/ 137.0/ 136.9 (q, bpy), 135.3/ 135.2/ 135.1 (q, PhCH2), 134.4/ 132.9/ 132.6 (q, Ph), 131.7/ 131.4/ 130.3 (py), 129.8/ 128.9/ 128.8/ 128.7/ 128.6/ 128.5/ 128.4/ 128.3/ 128.2/ 128.1/ 128.0/ 127.8/ 127.7 (Ph), 127.5/ 127.4/ 127.3/

127.1 (bpy), 125.5/ 125.0/ 124.7 (TRZ), 124.3/ 124.1/ 123.6 (py), 123.4/ 123.1 122.8/ 122. 6/ 122.1 (bpy), 72.4/ 72.3/ 70.3 (CHPh), 69.3/ 69.2/ 69.0 (CH_2-bpy), 68.9/ 68.6/ 68.4 (CH_2-CHPh), 61.8/ 61.5/ 61.4 (TRZCH_2O), 53.7/ 53.6/ 53.5 (Ph-CH_2-TRZ).

HRMS Calculated for $[Fe_2L_3]^{4+}$ m/z 464.1582, found m/z 464.1580

IR ν cm^{-1}3381 (br, s), 3031 (br, s), 1603 (m), 1556 (s), 1495 (m), 1468 (s), 1403 (m), 1361 (m), 1301 (m), 1220 (s), 1076 (s), 984 (m), 937 (m), 840 (w), 791 (w), 755 (w), 723 (w), 696 (m), 536 (w), 451 (m).

Elemental Analysis found (Calculated for $C_{105}H_{93}Cl_4Fe_2N_{21}O_6 \cdot 19H_2O$) % C 53.90 (53.88), H 4.53 (5.64), N 12.54 (12.57).

R_c,Δ_{Fe},HHT-$[Fe_2L^{3a}{}_3]Cl_4$.

Data as for *S*-enantiomer

Yield 0.18 g, 79%.

Elemental Analysis found (Calculated for $C_{105}H_{93}Cl_4Fe_2N_{21}O_6 \cdot 22H_2O$) % C 52.65 (52.66), H 4.25 (5.77), N 12.18 (12.28).

S_c,Λ_{Fe},HHT-$[Fe_2L^{3b}{}_3]Cl_4$.

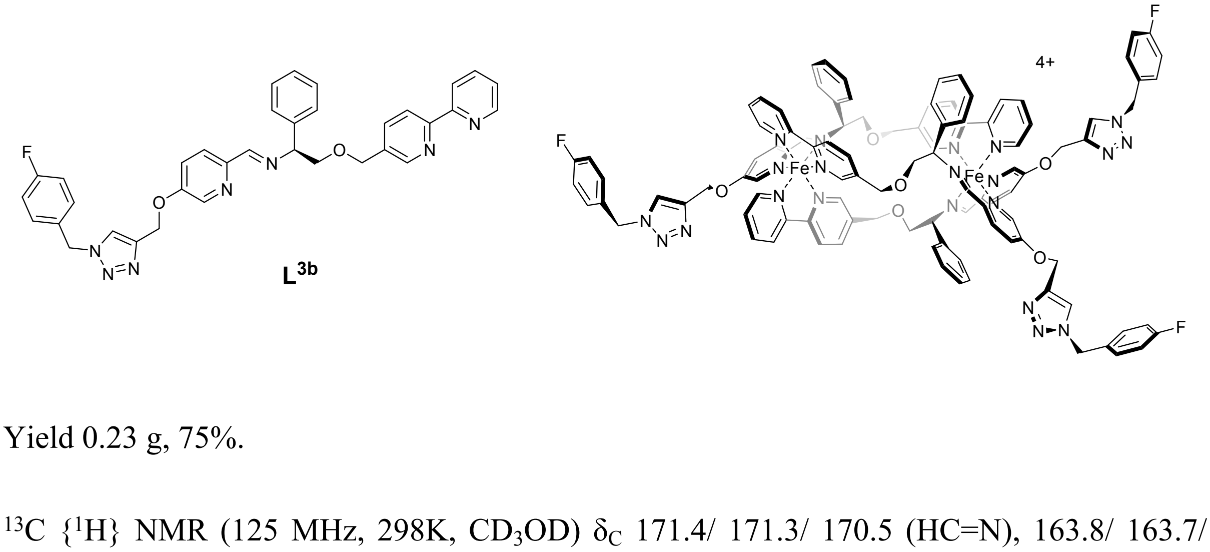

Yield 0.23 g, 75%.

^{13}C {^1H} NMR (125 MHz, 298K, CD_3OD) δ_C 171.4/ 171.3/ 170.5 (HC=N), 163.8/ 163.7/ 161.8/ 161.8 (q, F-Ph), 159.8/ 159.2/ 158.9/ 158.8/ 158.3/ 158.2 (q, bpy), 157.6 (bpy), 157.5 (q, py), 157.5 (bpy), 157.3/ 156.7 (q, py), 155.0 (bpy), 154.1/ 153.6/ 152.9 (py), 151.9/ 151.7/

151.6 (q, py), 143.3/ 142.7/ 142.3 (py), 140.0/ 139.9/ 139.8/ 138.9/ 138.5/ 138.3 (bpy), 1374/ 137.1/ 136.9 (q, bpy), 134.4/ 132.9/ 132.6 (q, Ph), 131.8/ 131.6 (py), 131.4/ 131.3/ 131.2 (q, F-Ph), 130.5/ 130.4/ 130.2/ 130.1/ 130.0 (F-Ph), 128.9/ 128.7/ 128.6/ 128.5 (Ph), 128.2/ 127.5/ 127.1 (bpy), 125.5/ 125.0/ 124.8 (TRZ), 124.3/ 124.0/ 123.6/ 123.5 (py/ bpy), 122.9/ 122.7/ 122.6/ 122.2 (bpy), 115.6, 115./ 115.4/ 115.3 (F-Ph), 72.4/ 72.3/ 70.3 (CHPh), 69.6/ 69.5/ 69.0 (CH_2-bpy), 68.9/ 68.7/ 68.5 (CH_2-CHPh), 61.8/ 61.6/ 61.5 (TRZCH_2O), 52.9/ 52.8/ 52.8 (F-Ph-CH_2-TRZ).

HRMS Calculated for $[Fe_2L_3]^{4+}$ m/z 477.6511, found m/z 477.6509

IR *v* cm^{-1} 3348 (br, m), 1603 (w), 1556 (s), 1509 (m), 1468 (m), 1218 (s), 1075 (s), 983 (m), 936 (m), 841 (m), 788 (m), 754 (m), 698 (s), 530 (s), 489 (s), 418 (s).

Elemental Analysis found (Calculated for $C_{105}H_{90}Cl_4F_3Fe_2N_{21}O_6 \cdot 19H_2O$) % C 52.75 (52.66), H 3.96 (5.39), N 12.17 (12.28).

R_c,Δ_{Fe},HHT-$[Fe_2L^{3b}{}_3]Cl_4$.

Data as for *S*-enantiomer

Yield 0.25 g, 82%.

Elemental Analysis found (Calculated for $C_{105}H_{90}Cl_4F_3Fe_2N_{21}O_6 \cdot 17H_2O$) % C 53.58 (53.47), H 4.20 (5.30), N 12.31 (12.47).

S_c,Λ_{Fe},HHT-$[Fe_2L^{3c}{}_3]Cl_4$.

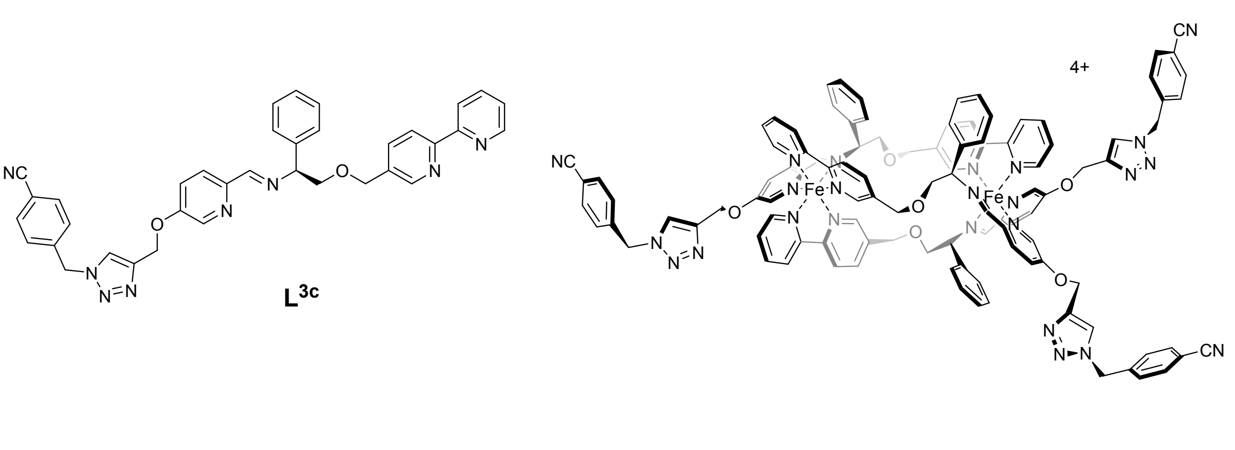

Yield 0.26 g, 84%.

$^{13}C\{^1H\}$ NMR (125 MHz, 298K, CD_3OD) δ_C 171.3/ 171.2/ 170.4 (HC=N), 159.8/ 159.2/ 158.9/ 158.8/ 158.3/ 158.2 (q, bpy), 157.6 (bpy), 157.5 (q, py), 157.4 (bpy), 157.3/ 156.7 (q, py), 155.0 (bpy), 154.1/ 153.6/ 152.9 (py), 152.0, 151.7/ 151.6 (q, py), 143.3/ 142.6/ 142.3 (py), 140.7/ 140.6 (q, CN-<u>Ph</u>), 140.0/ 139.9/ 139.8/ 138.9/ 138.5/ 138.3 (bpy), 137.4/ 137.1/ 136.9 (q, bpy), 134.4/ 132.9 (q, Ph), 132.64 (CN-<u>Ph</u>), 132.62 (q, Ph), 132.53/ 132.50 (CN-<u>Ph</u>), 131.8/ 131.6/ 130.4 (py), 129.1/ 129.0/ 128.9/ 128.7/ 128.7/ 128.6/ 128.5/ 128.4/ 128.3/ 128.2/ 128.1 (Ph), 127.5/ 127.1 (bpy), 125.5/ 125.2/ 125.1 (TRZ), 124.3/ 124.1/ 123.6/ 123.5 (py/ bpy), 122.9/ 122.6/ 122.2 (bpy), 117.9/ 117.86/ 117.84 (CN), 112.1/ 112.0 (q, CN-<u>Ph</u>), 72.4/ 72.36/ 70.32 (<u>C</u>HPh), 69.5/ 69.3/ 69.0 (<u>C</u>H_2-bpy), 68.9/ 68.7/ 68.4 (<u>C</u>H_2-CHPh), 61.7/ 61.6/ 61.4 (TRZ<u>CH_2</u>O), 53.0/ 52.8/ 52.7 (CN-Ph-<u>C</u>H_2-TRZ).

HRMS Calculated for $[Fe_2L_n]^{4+}$ m/z 482.9046, found m/z 482.9046

Elemental Analysis found (Calculated for $C_{108}H_{90}Cl_4Fe_2N_{24}O_6\cdot17H_2O$) % C 54.85 (54.51), H 4.15 (5.25), N 14.00 (14.13).

IR *ν* cm^{-1} 3356 (br, s), 2226 (m), 1606 (m), 1556 (s), 1468 (s), 1221 (s), 1111 (w), 1076 (s), 985 (m), 936 (w), 840 (w), 788 (m), 754 (m), 697 (m), 544 (s).

R_c,Δ_{Fe},HHT-$[Fe_2L^{3c}{}_3]Cl_4$.

Data as for *S*-enantiomer

Yield 0.25 g, 80%.

Elemental Analysis found (Calculated for $C_{108}H_{90}Cl_4Fe_2N_{24}O_6\cdot20H_2O$) % C 53.36 (53.30), H 4.07 (5.38), N 13.56 (13.81).

S_c,Λ_{Fe},HHT-$[Fe_2L^{3d}{}_3]Cl_4$.

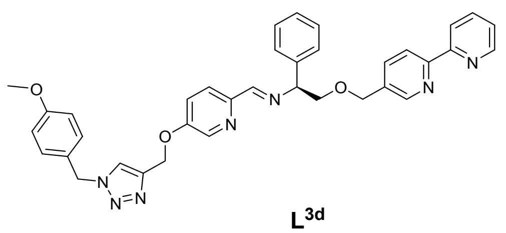

L^{3d}

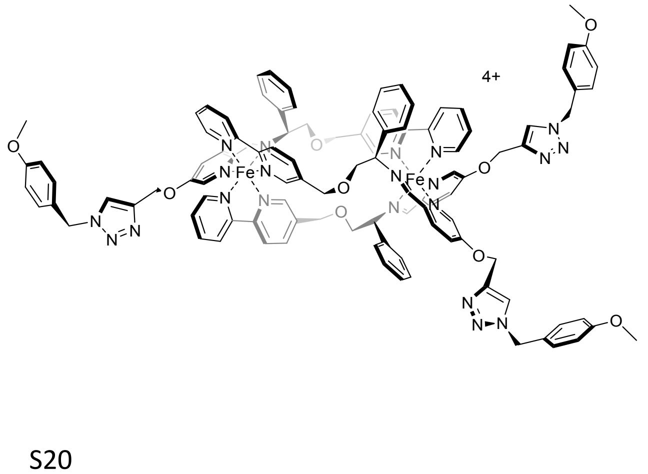

Yield 0.18 g, 88%.

^{13}C {^1H} NMR (125 MHz, 298K, CD_3OD) δ_C 171.4/ 171.3/ 170.5 (HC=N), 160.0 (q, <u>Ph</u>OCH_3), 159.9/ 159.2/ 158.9/ 158.8/ 158.7/ 158.3/ 158.2 (q, bpy), 157.6 (bpy), 157.5 (q, bpy), 157.4 (bpy), 157.3/ 157.2/ 156.7/ 156.6 (q, py), 155.0 (bpy), 154.1/ 153.6/ 152.9 (py), 152.0/ 151.7/ 151.6 (q, py) 143.2/ 142.5/ 142.3 (py) 139.9/ 139.8/ 139.3/ 138.9/ 138.5/ 138.3 (bpy), 137.4/ 137.1/ 136.9 (q, bpy), 134.4/ 132.9/ 132.6 (q, Ph), 131.78/ 131.6/ 130.4 (py), 128.9/ 129.8/ 129.7/ 129.6/ 129.5/ 129.4 (<u>Ph</u>OCH_3), 129.1/ 128.9/ 128.7/ 128.6/ 128.5/ 128.2/ 127.5 (Ph), 127.2/ 127.1/ 127.0 (q, <u>Ph</u>OCH_3), 125.5/ 124.8/ 124.4 (TRZ), 124.3/ 124.2/ 123.6 (py), 123.5/ 123.4/ 123.0/ 122.7/ 122.6/ 122.2 (bpy), 114.1/ 114.0/ 113. 9/ 113.8 (<u>Ph</u>OCH_3), 72.4/ 72.4/ 70.3 (<u>C</u>HPh), 69.5/ 69.4/ 69.0 (<u>C</u>H_2-bpy), 69.0/ 68.7/ 68.5 (<u>C</u>H_2-CHPh), 61.9/ 61.6/ 61.5 (TRZ<u>CH</u>$_2$O), 54.5 (OCH_3), 53.3/ 53.2/ 53.1 (CH_3OPh-<u>C</u>H_2-TRZ).

HRMS Calculated for $[Fe_2L_n]^{4+}$ m/z 486.6661, found m/z 486.6656

IR ν cm^{-1} 3361 (br, s), 1607 (m), 1556 (s), 1512 (s), 1467 (s), 1302 (m), 1241 (s), 1178 (m), 1076 (s), 1025 (m), 937 (w), 839 (w), 788 (m), 755 (m), 698 (m).

Elemental Analysis found (Calculated for $C_{108}H_{99}Cl_4Fe_2N_{21}O_9\cdot 22H_2O$) % C 52.18 (52.20), H 4.35 (5.80), N 11.53 (11.84).

R_c,Δ_{Fe},HHT-[$Fe_2L^{3d}{}_3$]Cl_4.

Data as for *S*-enantiomer

Yield 0.17 g, 85%.

Elemental Analysis found (Calculated for $C_{108}H_{99}Cl_4Fe_2N_{21}O_9\cdot 21H_2O$) % C 52.83 (52.58), H 4.46 (5.76), N 11.68 (11.92).

***S*c,ΛFe,HHT-[Fe2L3e3]Cl4.**

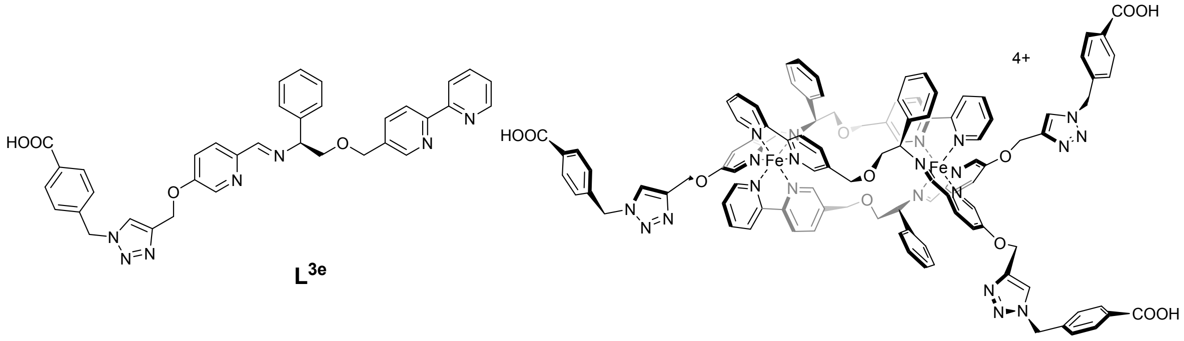

Yield 0.26 g, 77%.

$^{13}C\ \{^{1}H\}$ NMR (125 MHz, 298K, CD_3OD) δ_C 171.3/ 170.4 (HC=N), 159.8/ 159.2/ 158.8/ 158.3/ 158.2 (q, bpy), 157.5 (bpy), 157.46 (q, py), 157.4 (bpy), 157.2/ 156.7 (q, py), 155.0 (bpy), 154.1/ 153.5/ 152.9 (py), 152.0/ 151.7/ 151.5 (q, py), 143.3/ 142.3/ 142.1 (py), 140.1/ 140.0 (q, <u>Ph</u>COOH), 139.9/ 139.7/ 138.9/ 138.5/ 138.3 (bpy), 137.4/ 137.0/ 136.9 (q, bpy), 134.3/ 132.9/ 132.6 (q, Ph), 131.7/ 130.3 (py), 128.9/ 128.7/ 128.6 128.5/ 128.1/ 128.0/ 127.5/ 127.1 (Ph), 125.5/ 125.1 (TRZ), 124.6/ 124.3/ 123.6 (py), 123.5/ 123.1/ 122.8/ 122.6/ 122.1 (bpy), 72.4/ 72.3/ 70.3 (<u>C</u>HPh), 69.3/ 69.0 (<u>C</u>H_2-bpy), 68.9/ 68.6/ 68.4 (<u>C</u>H_2-CHPh), 61.8/ 61.6/ 61.4 (TRZ<u>CH2</u>O), 53.2/ 53.1/ 53.0 (COOHPh-<u>C</u>H_2-TRZ)

HRMS Calculated for $[Fe_2L_n]^{4+}$ m/z 497.1505, found m/z 497.1528

IR ν cm^{-1}3360 (br, w), 1699 (m), 1606 (m), 1556 (s), 1467 (m), 1373 (m), 1220 (s), 1178 (m), 1110 (m), 1076 (s), 1053 (m), 984 (m), 936 (m), 839 (m), 750 (s), 731 (s), 697 (s).

Elemental Analysis found (Calculated for $C_{108}H_{93}Cl_4Fe_2N_{21}O_{12}\cdot 16H_2O$) % C 53.67 (53.63), H 4.18 (5.21), N 11.95 (12.16).

***R*c,ΔFe,HHT-[Fe2L3e3]Cl4.**

Data as for *S*-enantiomer

Yield 0.25 g, 75%.

Elemental Analysis found (Calculated for $C_{108}H_{93}Cl_4Fe_2N_{21}O_{12}\cdot 17H_2O$) % C 53.25 (53.23), H 4.17 (5.25), N 11.98 (12.07).

2. NMR Spectra

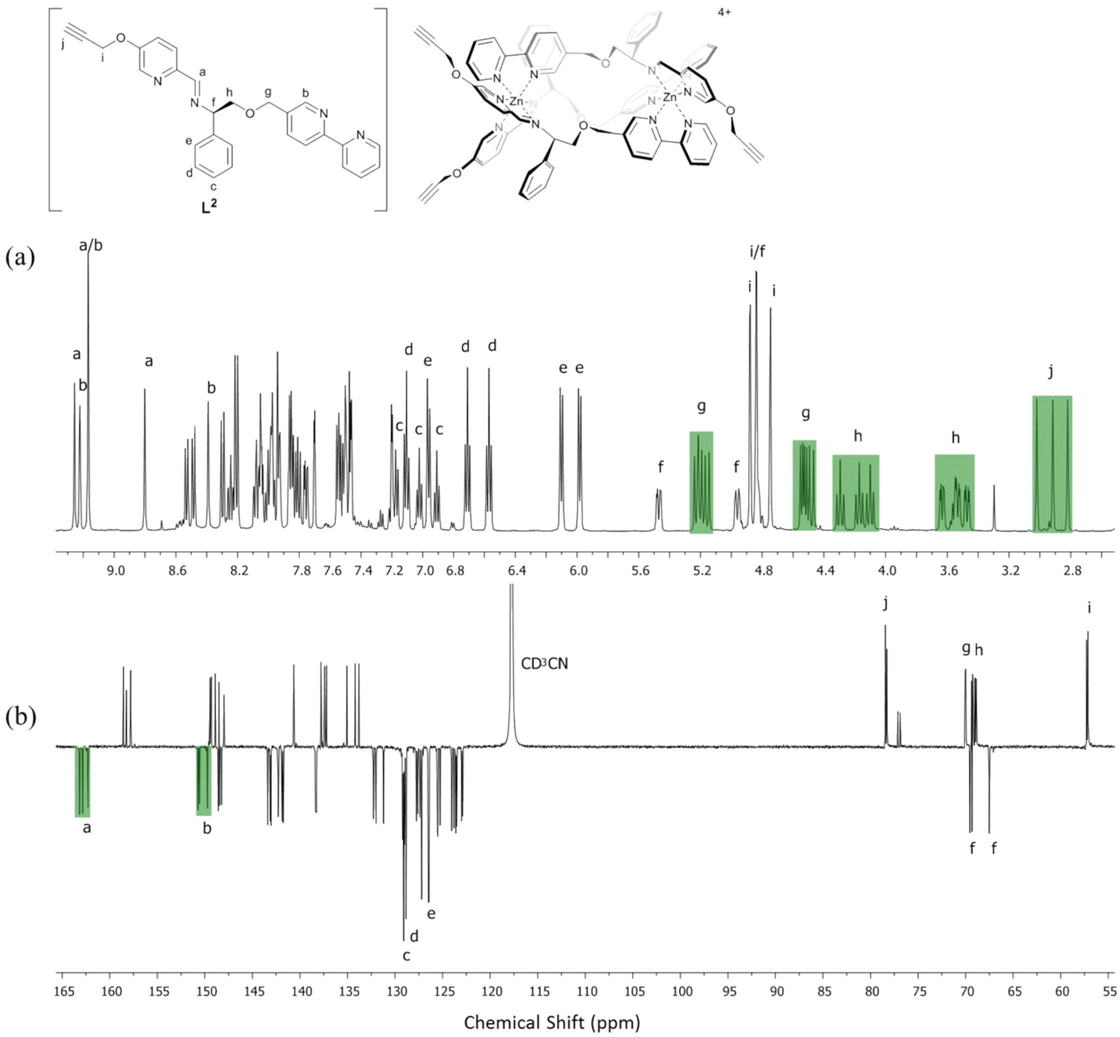

Supplementary Figure 1 ^{1}H (500 MHz, CD_3CN, 298K) and ^{13}C (125 MHz, CD_3CN, 298K) NMR spectra of R_c,Δ_{Zn},HHT-$[Zn_2\mathbf{L^2}_3][ClO_4]_4$

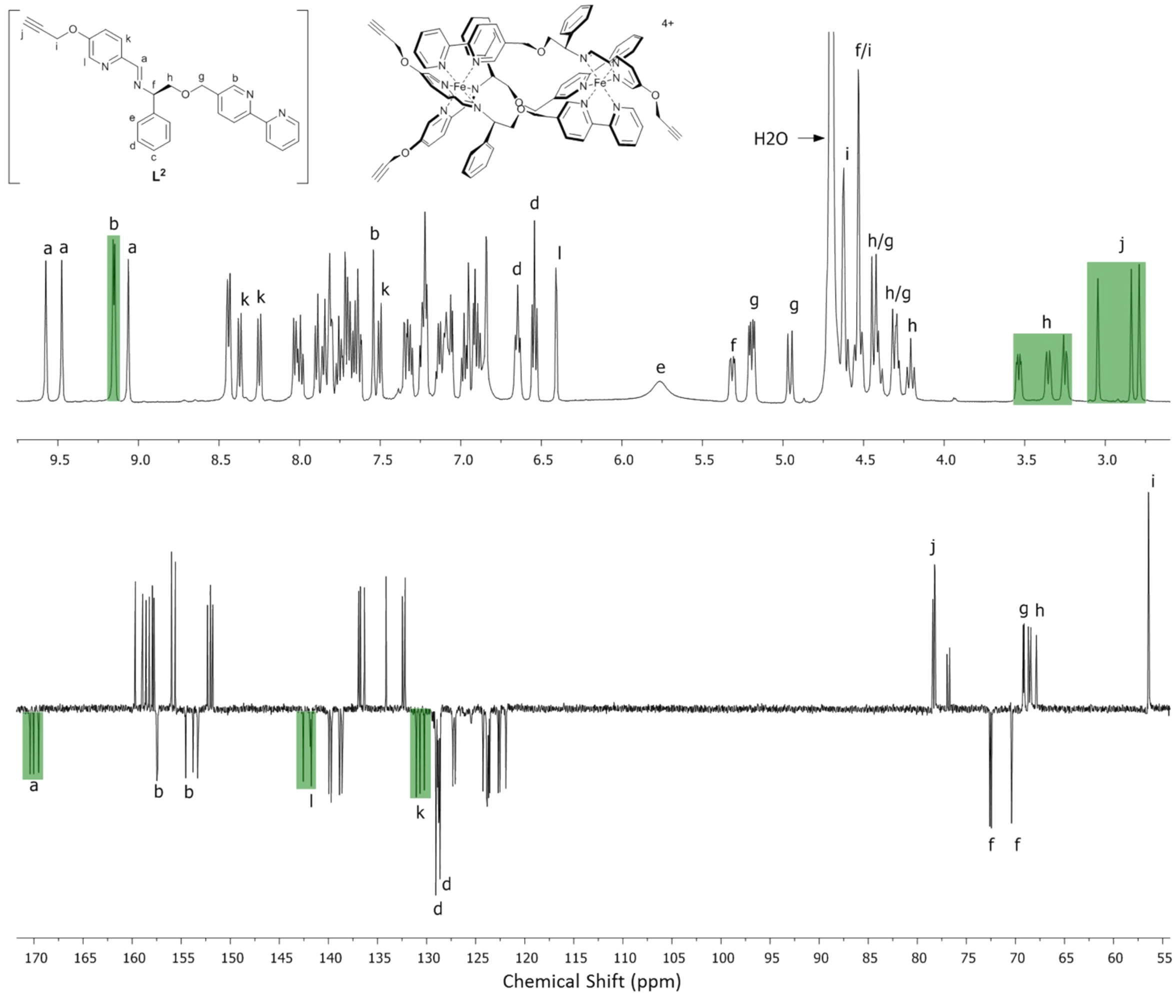

Supplementary Figure 2 ^{1}H (500 MHz, D_2O, 298K) and ^{13}C (125 MHz, D_2O, 298K) NMR spectra of R_c,Δ_{Fe},HHT-[Fe_2**L**$^{2}_{3}$]Cl_4

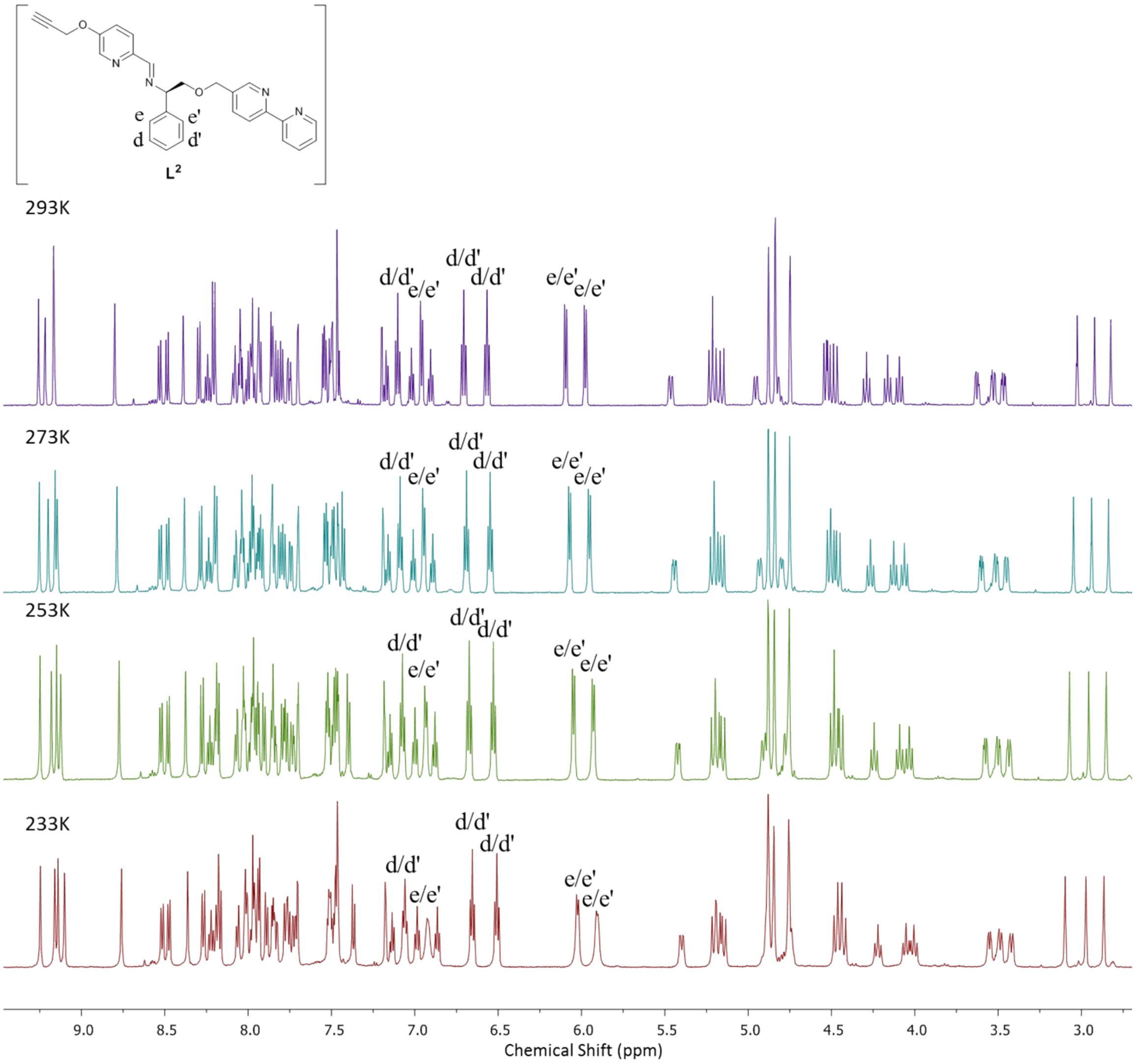

Supplementary Figure 3 Variable temperature ^{1}H NMR spectra of (R_c,Δ_{Zn})-HHT-[Zn_2**L**$^2{}_3$][ClO_4]$_4$ (600 MHz, CD_3CN)

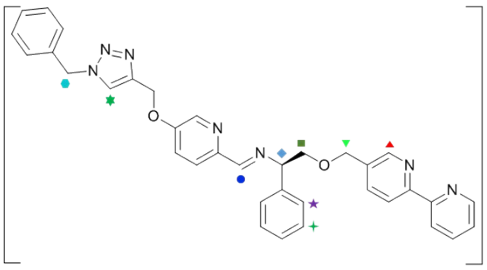

L[3a]

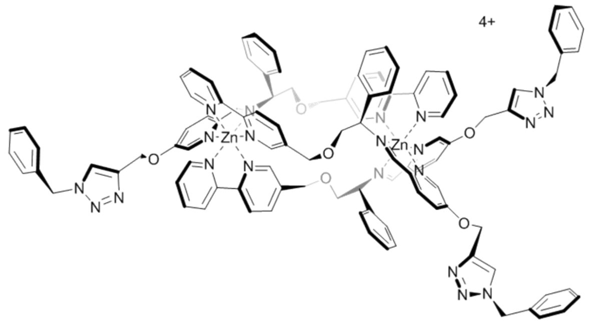

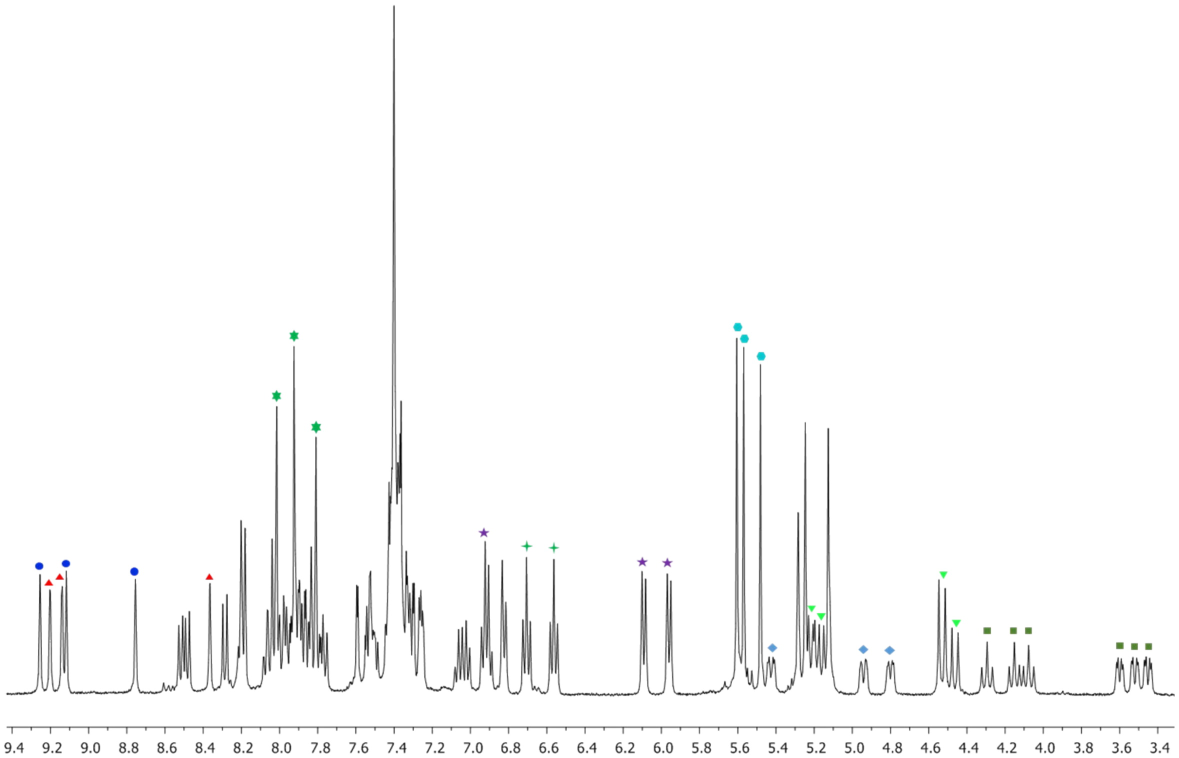

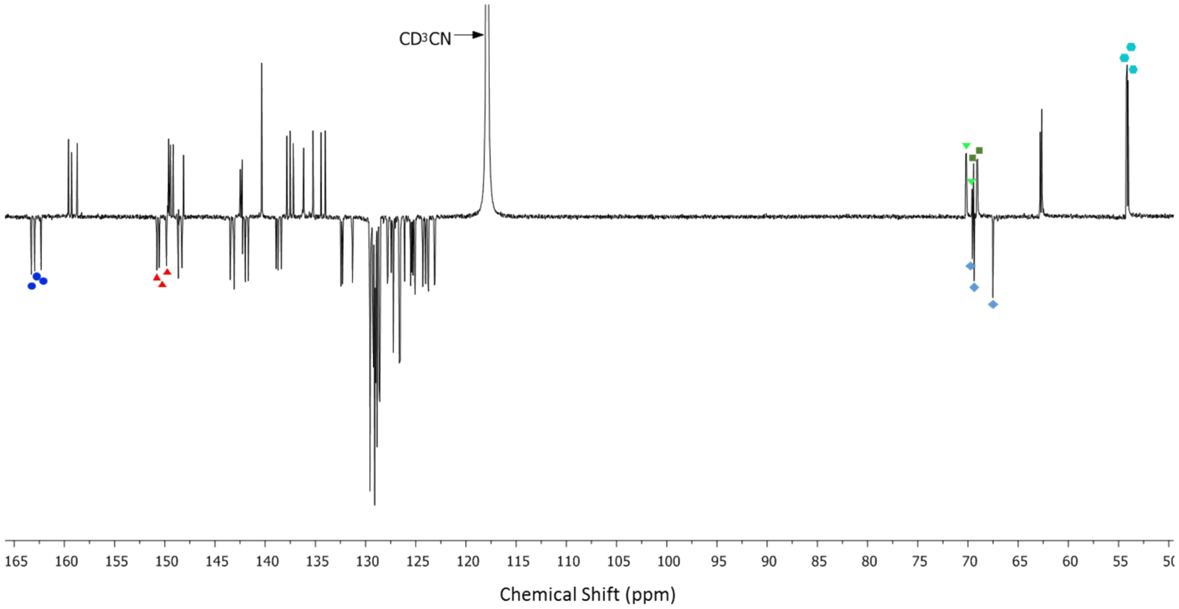

Supplementary Figure 4 ^{1}H (500 MHz, CD_3CN, 298K) and ^{13}C (125 MHz, CD_3CN, 298K) NMR spectra of R_c,Δ_{Zn},HHT-[$Zn_2$$\mathbf{L}^{3a}{}_3$][$ClO_4$]$_4$

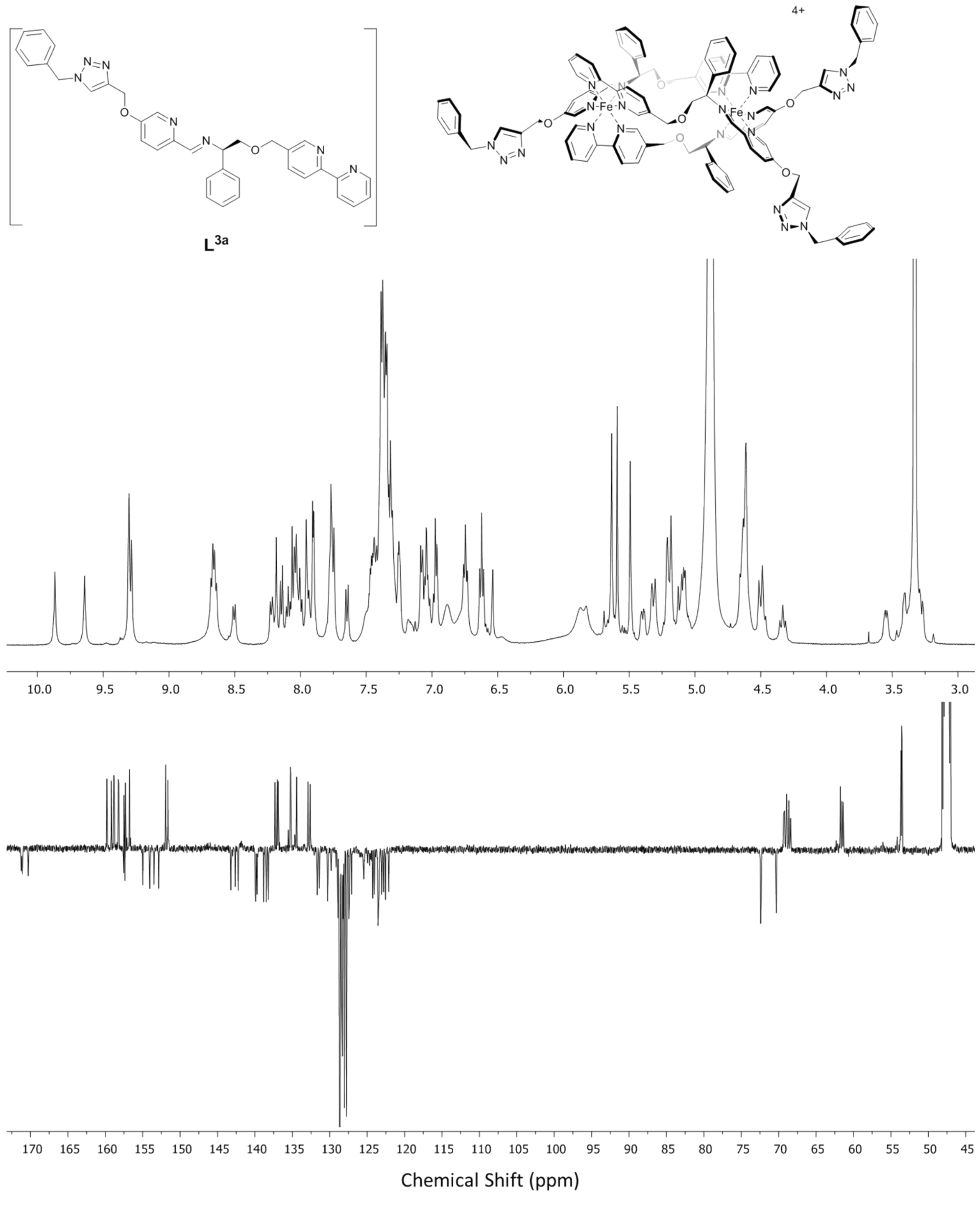

Supplementary Figure 5 ^{1}H (500 MHz, CD_3OD, 298K) and ^{13}C (125 MHz, CD_3OD, 298K) NMR spectra of R_c,Δ_{Fe},HHT-[Fe_2**L**$^{3a}{}_3$]Cl_4

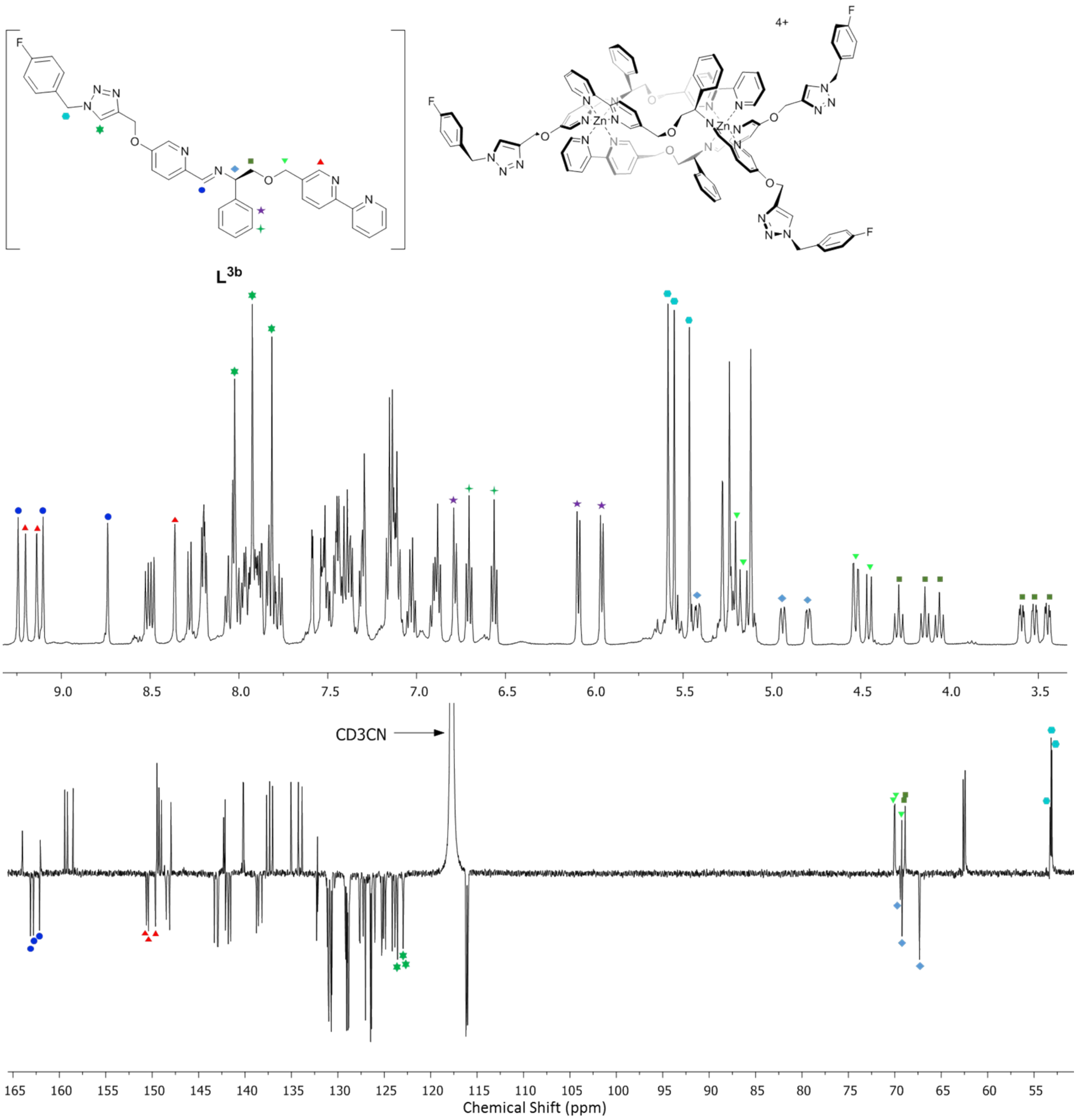

Supplementary Figure 6 ^{1}H (500 MHz, CD_3CN, 298K) and ^{13}C (125 MHz, CD_3CN, 298K) NMR spectra of R_c,Δ_{Zn},HHT-[Zn_2**L**$^{3b}{}_3$][ClO_4]$_4$

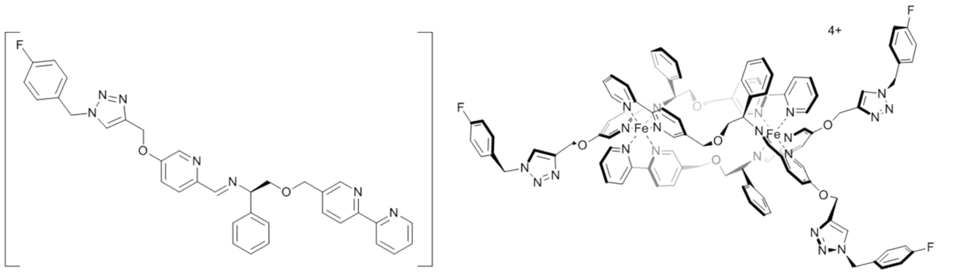

$\mathbf{L^{3b}}$

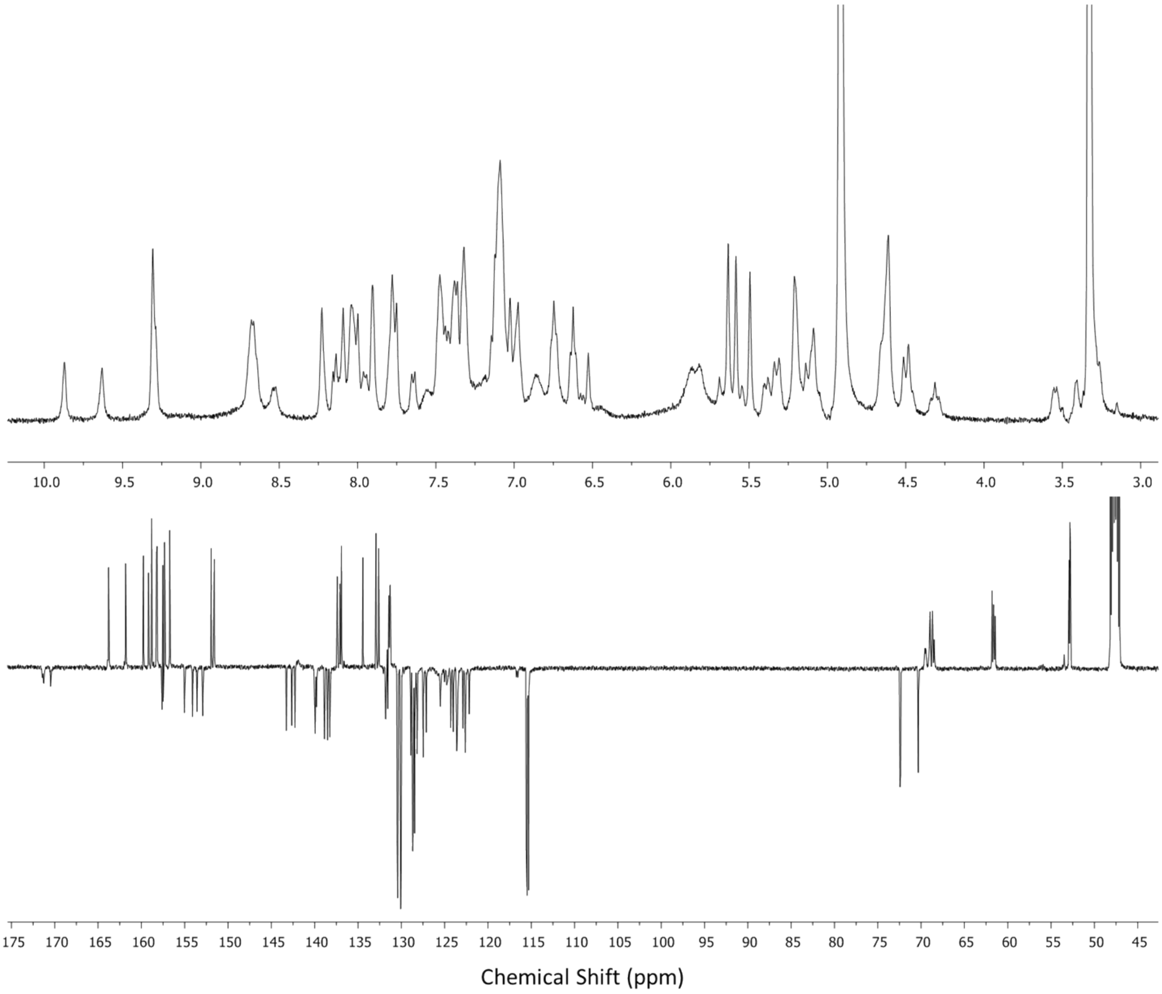

Supplementary Figure 7 ^{1}H (500 MHz, CD_3OD, 298K) and ^{13}C (125 MHz, CD_3OD, 298K) NMR spectra of R_c,Δ_{Fe},HHT-[$Fe_2$$\mathbf{L^{3b}}_3$]$Cl_4$

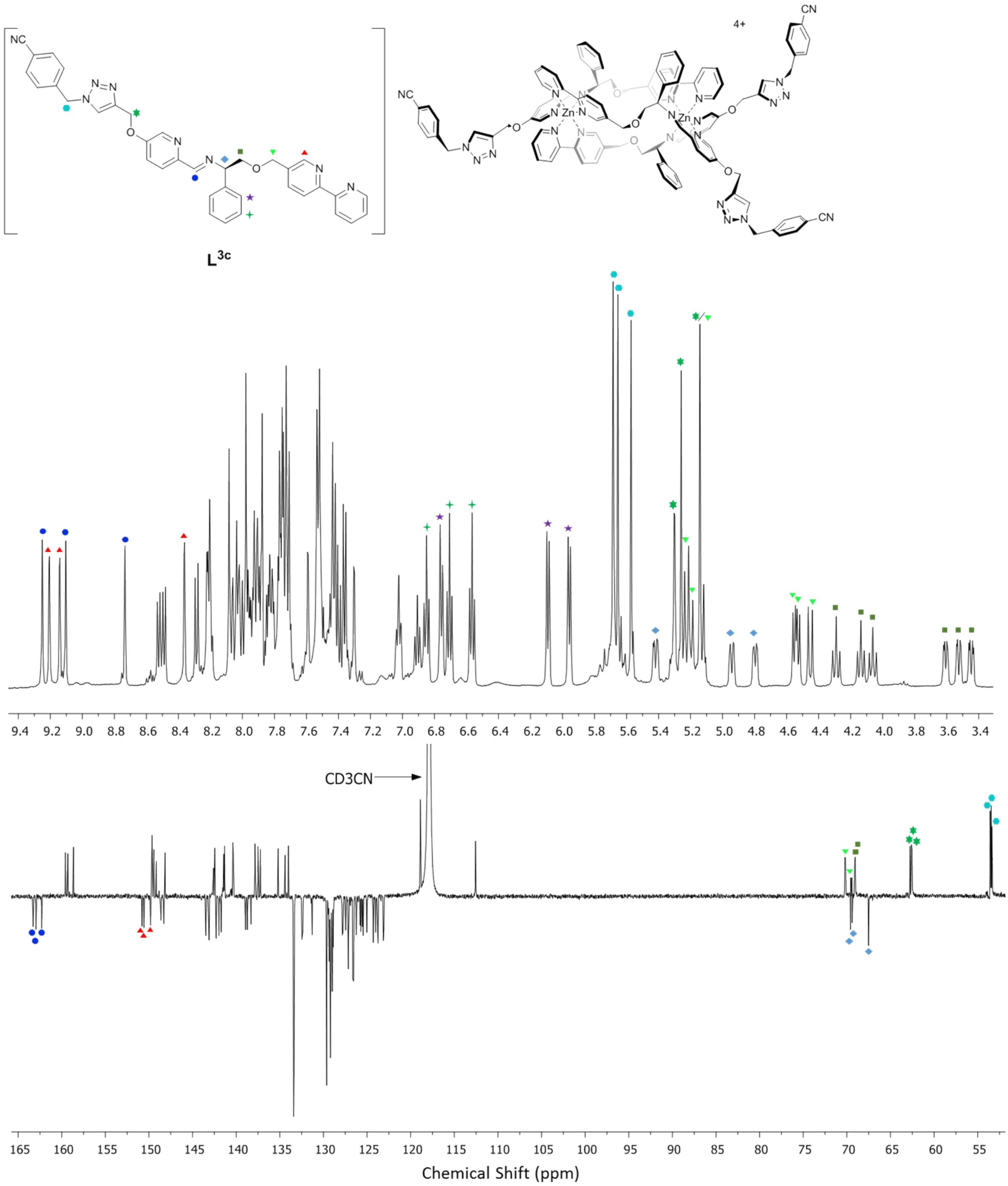

Supplementary Figure 8 ^{1}H (500 MHz, CD_3CN, 298K) and ^{13}C (125 MHz, CD_3CN, 298K) NMR spectra of R_c,Δ_{Zn},HHT-[Zn_2**L**$^{3c}{}_3$][ClO_4]$_4$

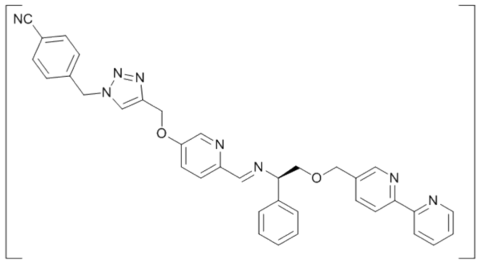

L[3c]

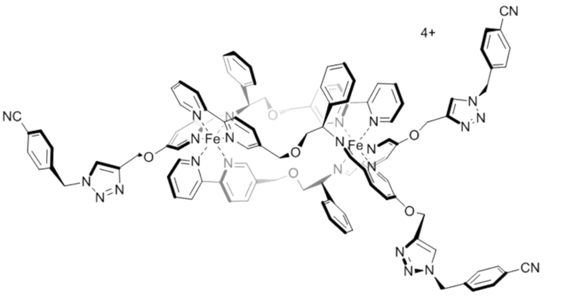

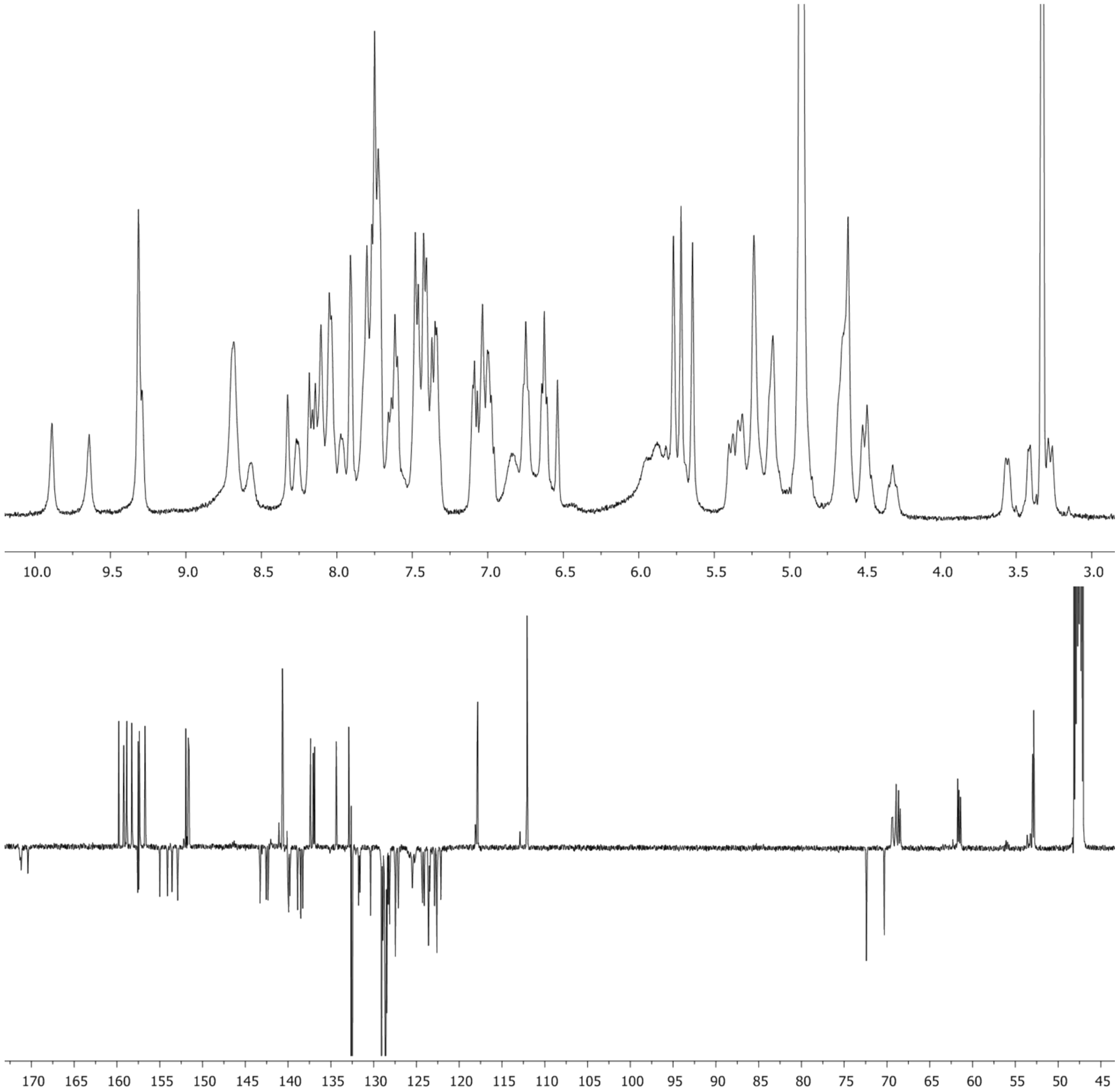

Supplementary Figure 9 ^{1}H (500 MHz, CD_3OD, 298K) and ^{13}C (125 MHz, CD_3OD, 298K) NMR spectra of R_c,Δ_{Fe},HHT-[Fe_2**L**$^{3c}{}_3$]Cl_4

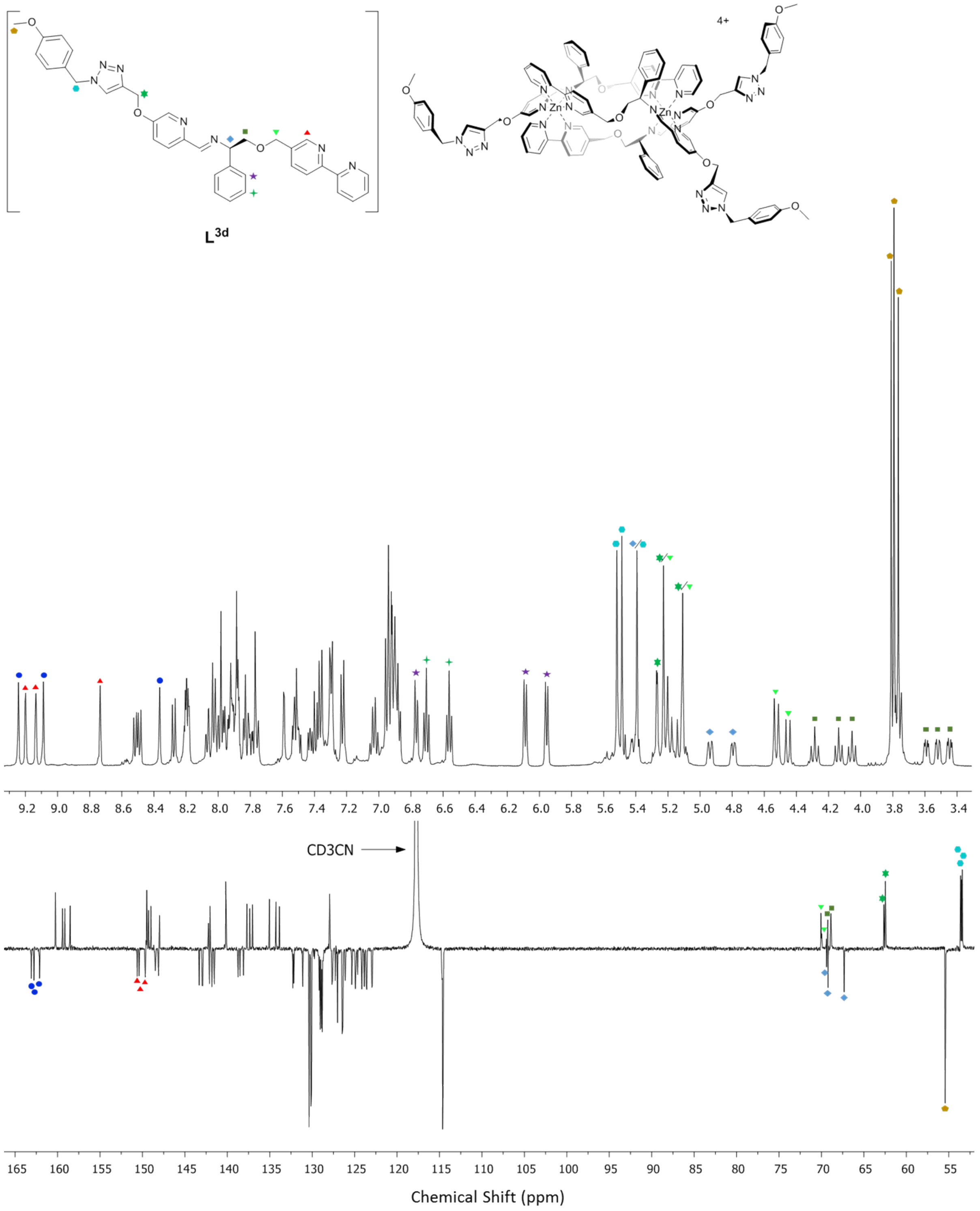

Supplementary Figure 10 ^{1}H (500 MHz, CD_3CN, 298K) and ^{13}C (125 MHz, CD_3CN, 298K) NMR spectra of R_c,Δ_{Zn},HHT-$[Zn_2\mathbf{L^{3d}}_3][ClO_4]_4$

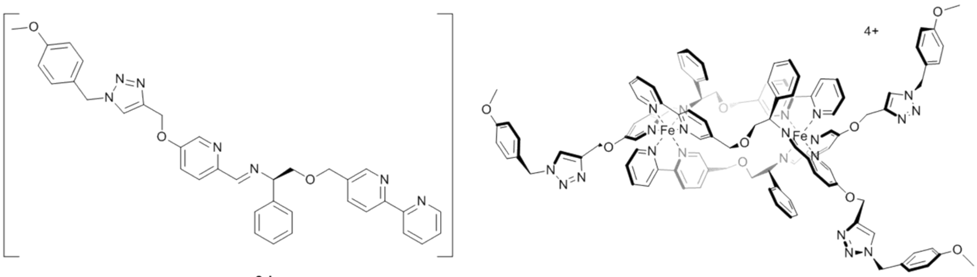

L^{3d}

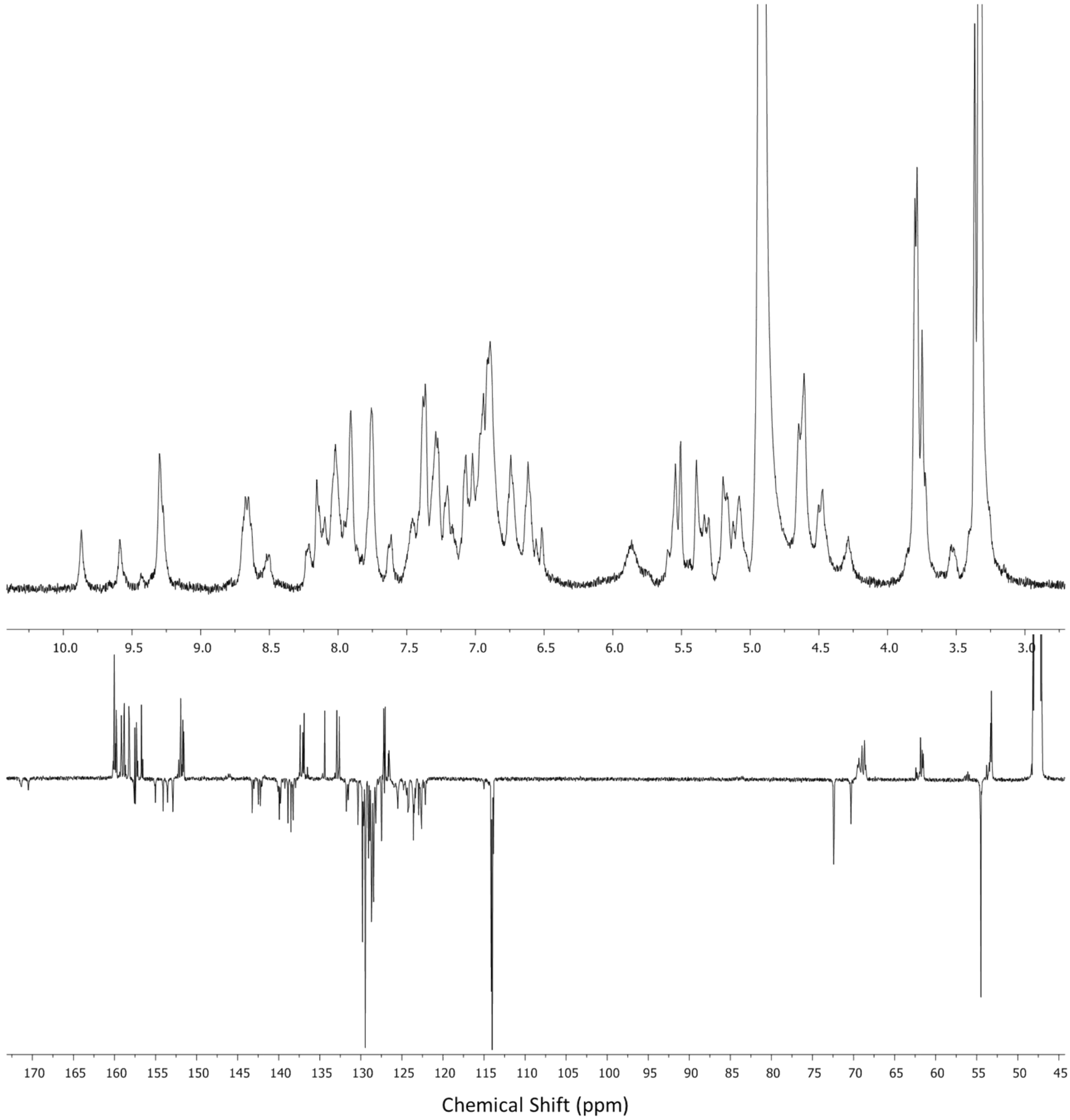

Supplementary Figure 11 ^{1}H (500 MHz, CD_3OD, 298K) and ^{13}C (125 MHz, CD_3OD, 298K) NMR spectra of R_c,Δ_{Fe},HHT-[Fe_2**L^{3d}**$_3$]Cl_4

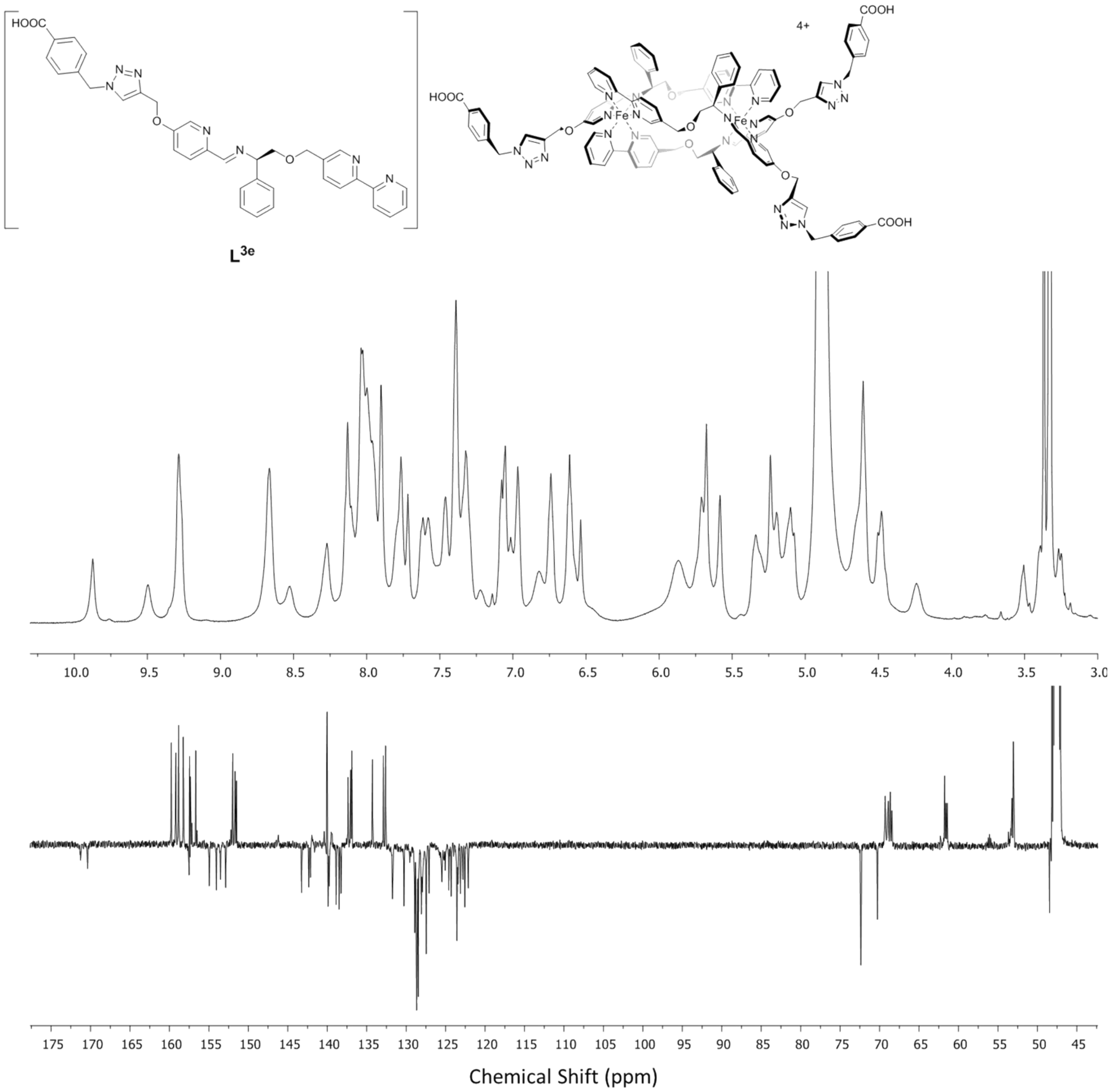

Supplementary Figure 12 ^{1}H (500 MHz, CD_3OD, 298K) and ^{13}C (125 MHz, CD_3OD, 298K) NMR spectra of R_c,Δ_{Fe},HHT-[Fe_2**L**$^{3e}_3$]Cl_4

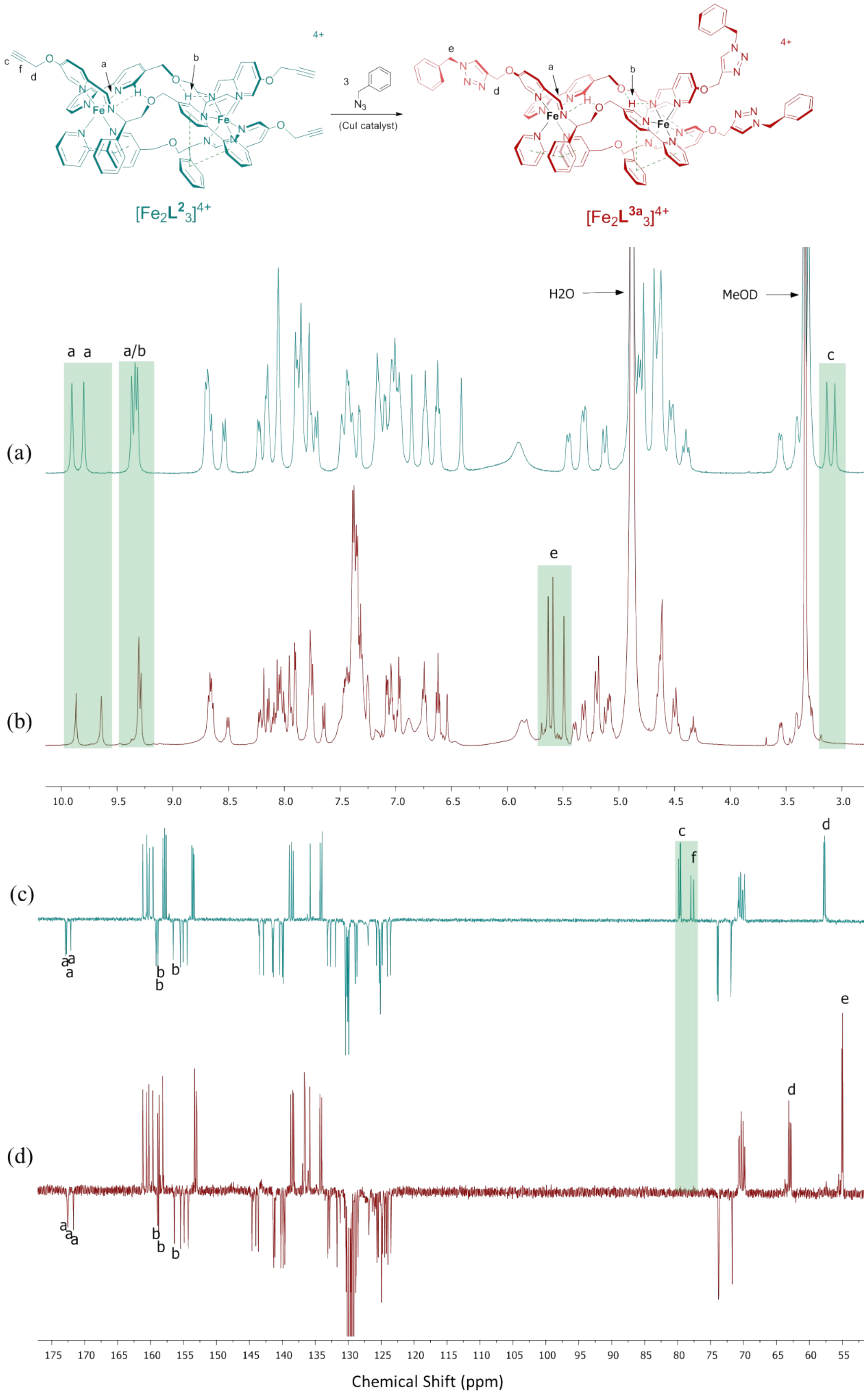

Supplementary Figure 13 ^{1}H NMR spectra (400 MHz, CD_3OD, 298K) of (a) R_c,Δ_{Fe},HHT-[Fe_2**L**$^2{}_3$]Cl_4, (b) R_c,Δ_{Fe},HHT-[Fe_2**L**$^{3a}{}_3$]Cl_4; ^{13}C NMR spectra (125 MHz, CD_3OD, 298K) of (c) R_c, Δ_{Fe},HHT[Fe_2**L**$^2{}_3$]Cl_4, (d) R_c, Δ_{Fe},HHT-[Fe_2**L**$^{3a}{}_3$]Cl_4;

3. ICP-MS [Cu] test for S_c,Λ_{Fe},HHT-[$Fe_2L^{3b}{}_3$]Cl_4.

ICPMS conditions: Instrument: Agilent 7500cx, equipped with Auto-sampler. Plasma condition: Plasma gas: 15 L/min, carrier gas: 0.9 L/min, make-up gas: 0.15 L/min, nebuliser gas: 0.8 L/min, Spray chamber temperature: 15°C, He mode gas flow 4.5 ml/min, Forward power 15500 W, flow rate: 0.08 rpm, Detector analogue HV 1720V, pulse HV 1120V,

Calibration range: 0, 10, 25, 50, 100, 250, 500 and 1000 ppb.

Sample (3 mg) was dissolved into 1 ml of 50% ACN with 0.1% Formic Acid, then diluted to 10 ml with 2% nitric acid in water and centrifuged for 5 minutes at 14000 rpm. The supernatant is then diluted 100 folds with water before analysis on the ICP-MS. Calibration range 0, 10, 25, 50, 100, 250, 500 and 1000 ppb.

Concentration found in the 100 times diluted solution to be 15.83 ppb with an RSD at 0.87%, which means the Cu content in the original sample is 0.527±0.005%.

4. Stability tests using NMR:

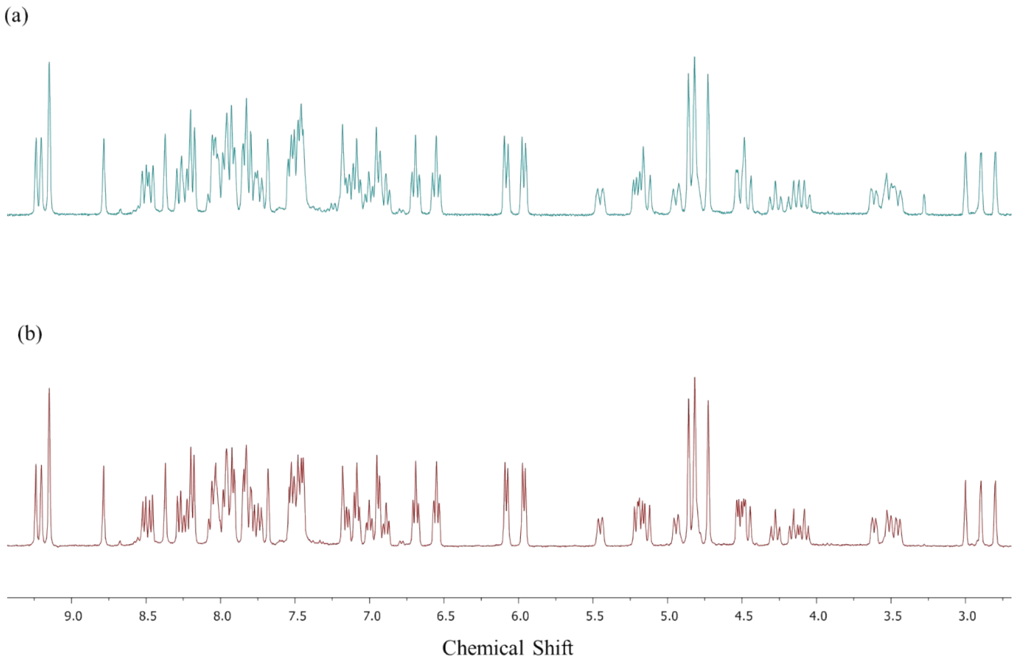

Supplementary Figure 14: ^{1}H NMR spectra (CD_3CN, 298K)of R_c,Δ_{Zn},HHT-[Zn_2**L**$^{2}{}_3$][ClO_4]$_4$: (a) test in 300 MHz; (b) re-test in 400 MHz after two months

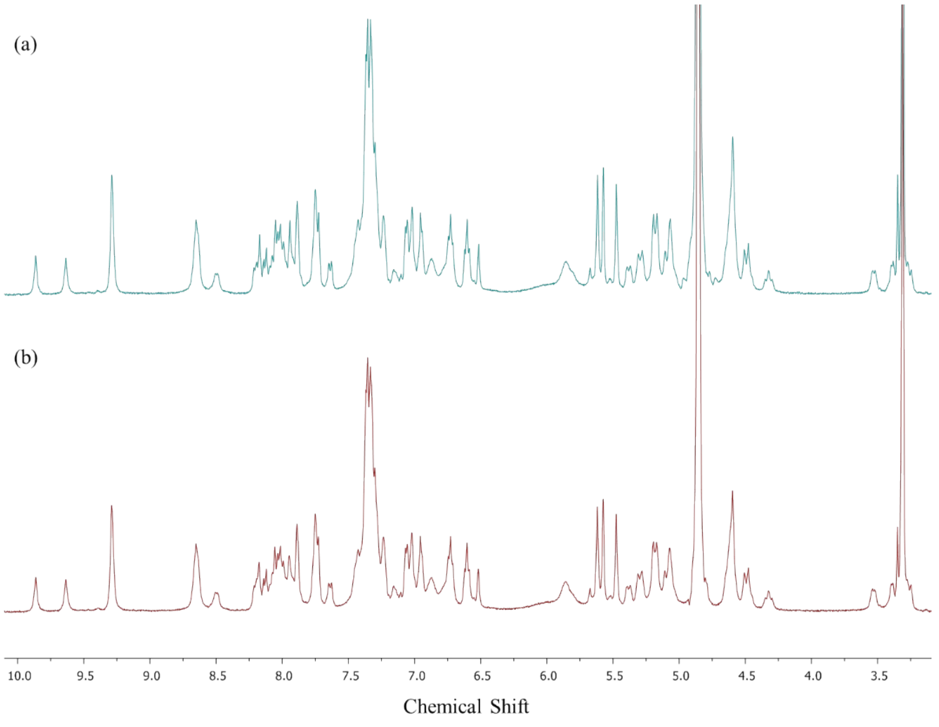

Supplementary Figure 15: ^{1}H NMR spectra (CD_3OD, 298K) of R_c,Δ_{Fe},HHT-[Fe_2**L**$^{3a}{}_3$]Cl_4: (a) test in 400 MHz; (b) re-test in 400 MHz after two months

5. Circular Dichroism Spectra

Circular Dichroism Spectra were measured on a JascoJ-815 spectrometer, calibrated conventionally using 0.060% ACS a holmium filter. Measurements were collected using a 1 cm path-length quartz cuvette. The parameters used were; bandwidth 1 nm, response time 1 sec, wavelength scan range 200 – 700 nm, data pitch 0.2 nm, scanning speed 100 nm/min and accumulation 8. For CD spectra of $[Fe_2\mathbf{L^1}_3]Cl_4$ see reference 6.

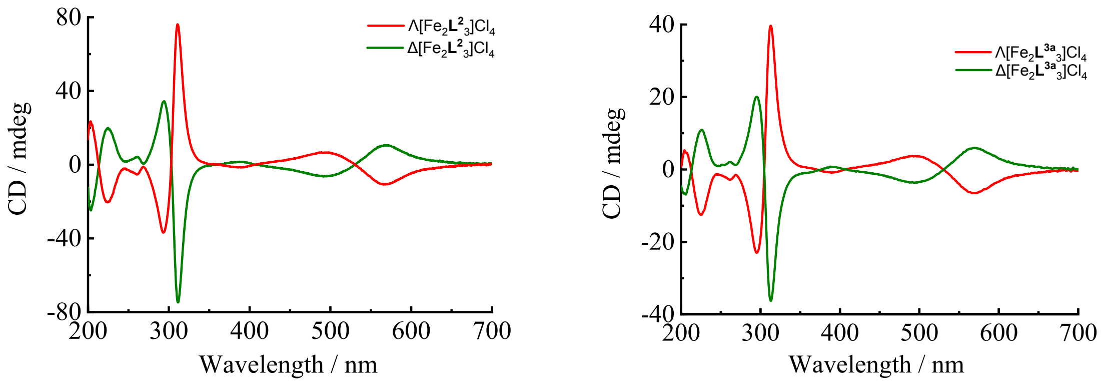

Supplementary Figure 16. CD spectra of the pairs of enantiomers of triplex metallohelices (0.1 mg/mL in methanol); each enantiomer shows an equal and opposite spectrum to its pair.

6. Stability study of S_c,Λ_{Fe},HHT-[$Fe_2L^2_3$]Cl_4.and S_c,Λ_{Fe},HHT-[$Fe_2L^{3a}_3$]Cl_4 in aqueous solutions by UV-Vis Absorbance Spectroscopy

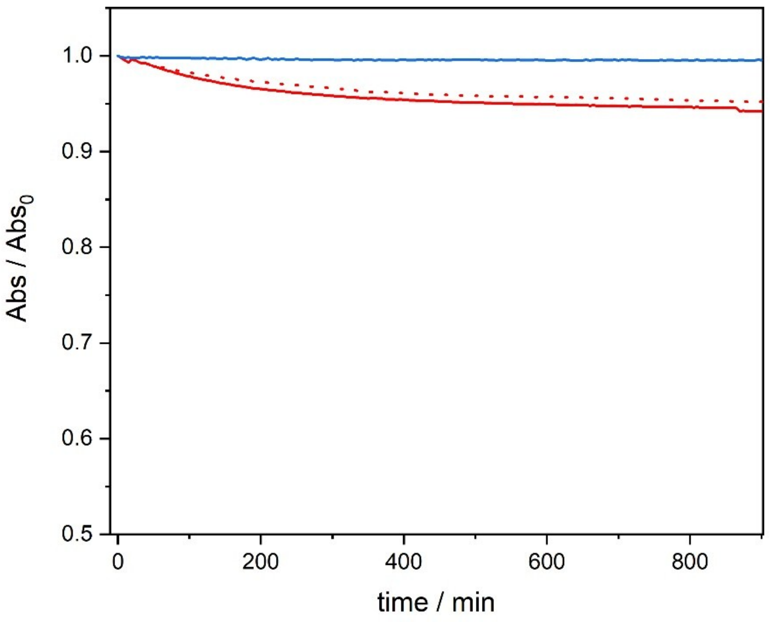

Supplementary Figure 17 Monitoring MLCT absorption of the metallohelices S_c,Λ_{Fe},HHT-[$Fe_2\mathbf{L}^2_3$]Cl_4 (red), and S_c,Λ_{Fe},HHT-[$Fe_2L^{3a}_3$]Cl_4 (blue) in HCl/KCl buffer at pH 1.5 (solid line) and phosphate buffer saline at pH 7.0 (dotted line) labs = 530 nm, solutions 0.02 mg mL^{-1}.

After 5 hr a drop in intensity of 4 % was observed for S_c,Λ_{Fe},HHT-[$Fe_2\mathbf{L}^2_3$]Cl_4 and 0.5 % for S_c,Λ_{Fe},HHT-[$Fe_2\mathbf{L}^{3a}_3$]Cl_4 at pH 1.5.

7. High Resolution ESI mass spectra

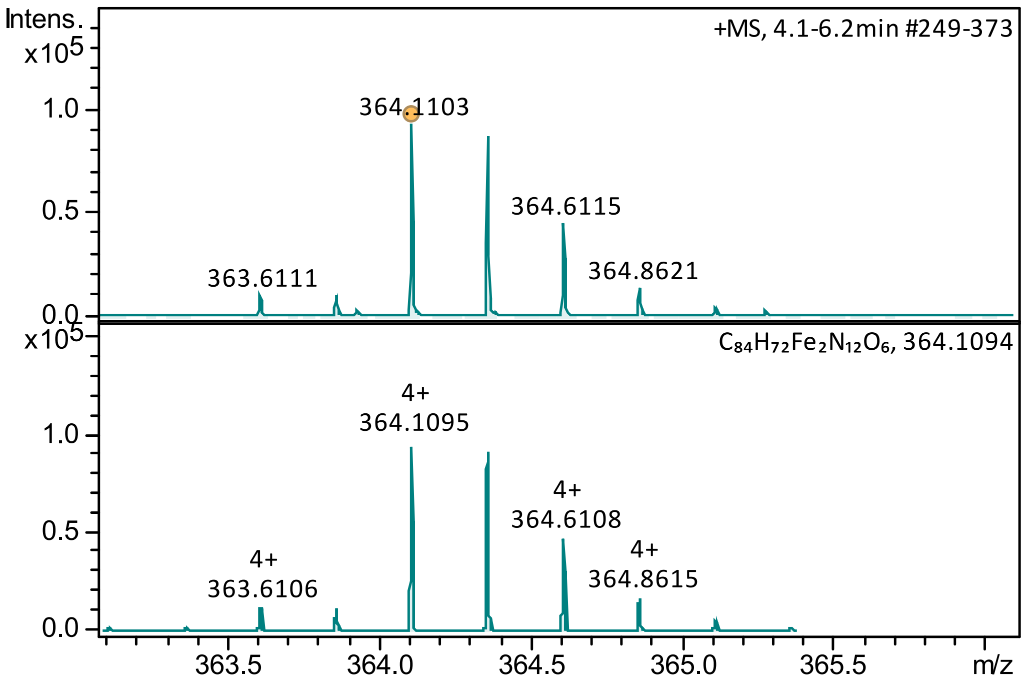

Supplementary Figure 18. High resolution ESI mass spectrum of Δ_{Fe},HHT-[Fe_2**L**$^2{}_3$]Cl_4 showing the observed z = +4 charge (top), compared to the theoretical isotope pattern (bottom).

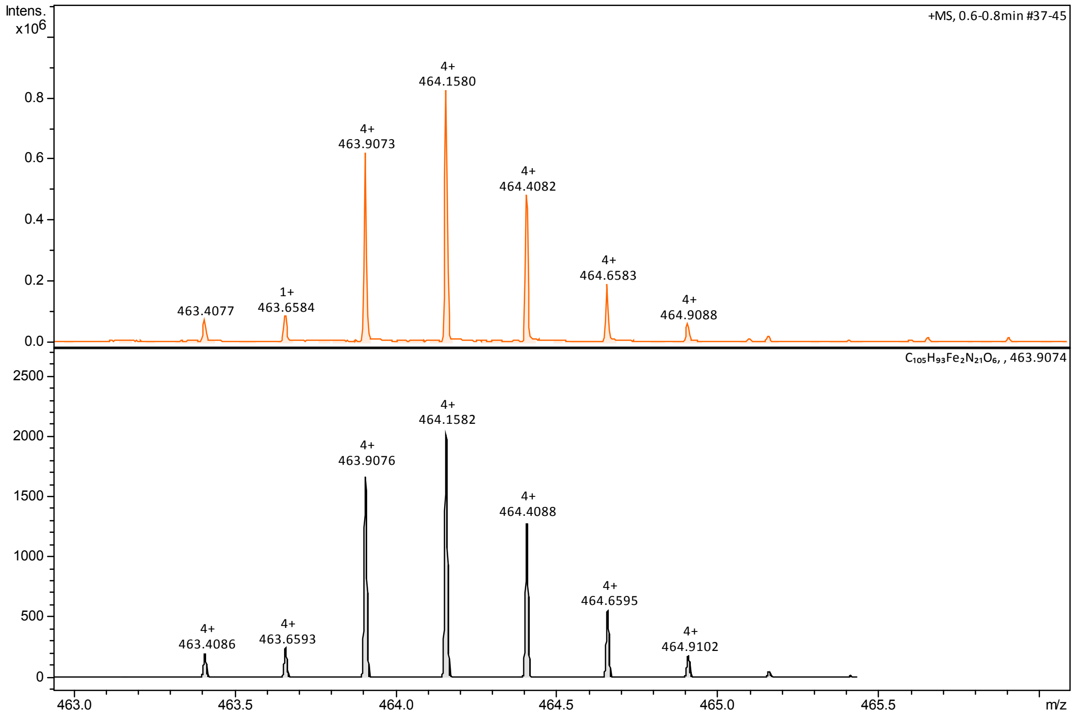

Supplementary Figure 19. High resolution ESI mass spectrum of Λ_{Fe},HHT-[Fe_2**L**$^{3a}{}_3$]Cl_4 showing the observed z = +4 charge (top), compared to the theoretical isotope pattern (bottom).

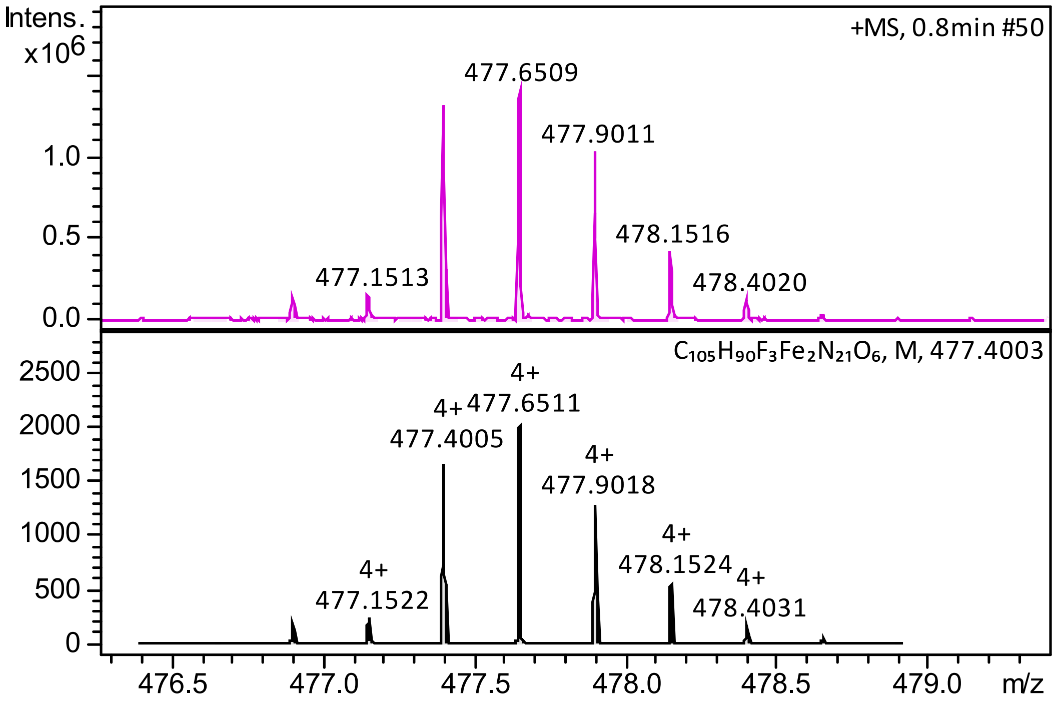

Supplementary Figure 20. High resolution ESI mass spectrum of Λ_{Fe},HHT-[Fe_2**L**^{3b}$_3$]Cl_4 showing the observed z = +4 charge (top), compared to the theoretical isotope pattern (bottom).

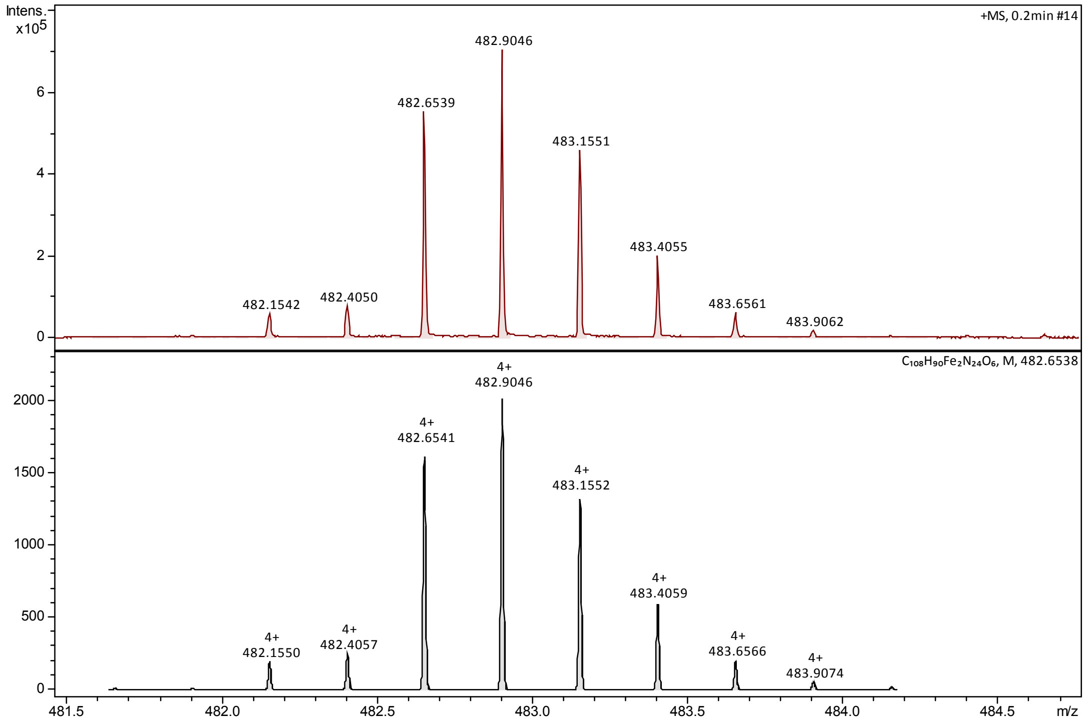

Supplementary Figure 21. High resolution ESI mass spectrum of Λ_{Fe},HHT-[Fe_2**L**^{3c}$_3$]Cl_4 showing the observed z = +4 charge (top), compared to the theoretical isotope pattern (bottom).

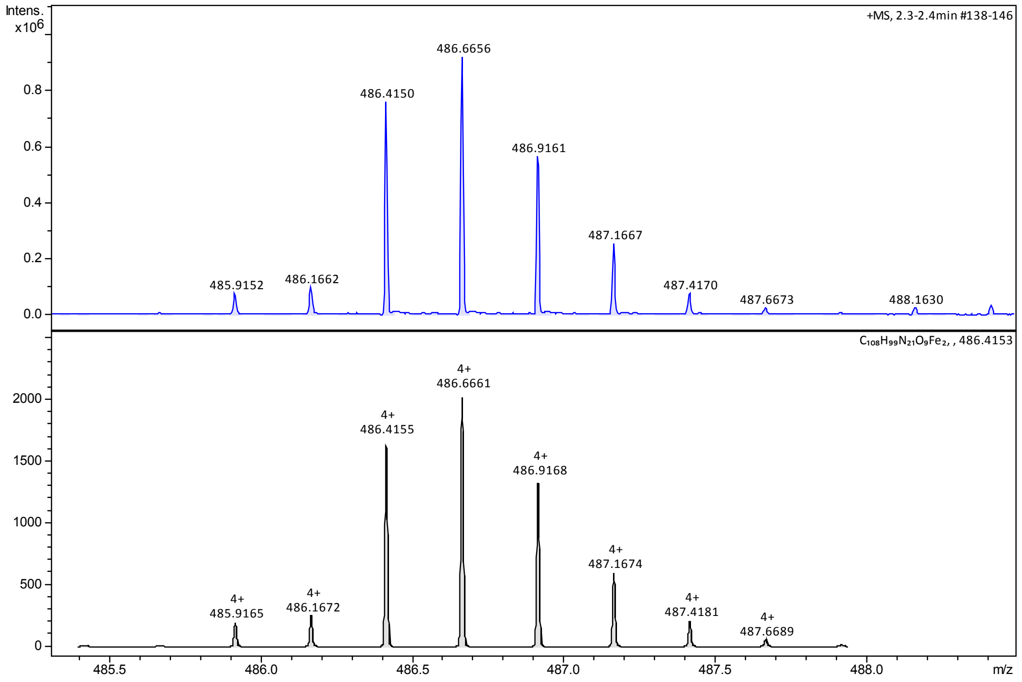

Supplementary Figure 22. High resolution ESI mass spectrum of Λ_{Fe},HHT-[Fe_2**L**^{3d}$_3$]Cl_4 showing the observed z = +4 charge (top), compared to the theoretical isotope pattern (bottom).

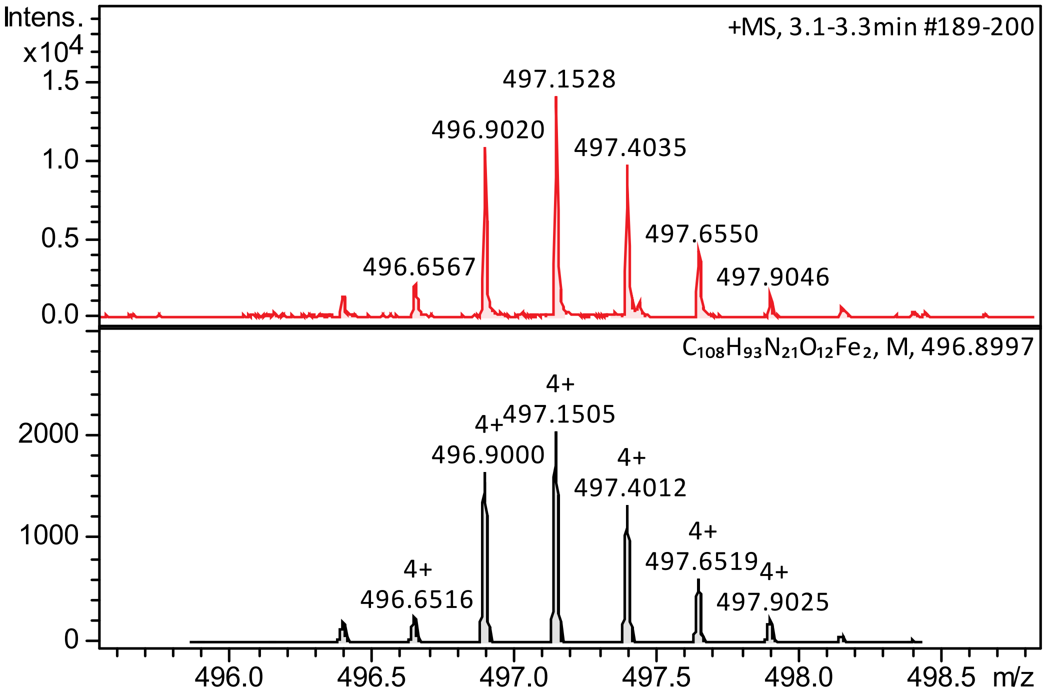

Supplementary Figure 23. High resolution ESI mass spectrum of Λ_{Fe},HHT-[Fe_2**L**^{3e}$_3$]Cl_4 showing the observed z = +4 charge (top), compared to the theoretical isotope pattern (bottom).

8. Anticancer Experiments

8.1 Chemosensitivity (MTT assay)

The cells were seeded in 96-well tissue culture plates at a density of 1×10^4 A2780/cisR cells/ml, 8×10^3 MCF-7 cells/ml, 5×10^3 HeLa cells/ml, 5×10^3 MDA-MB-231 cells/ml, 1×10^4 MRC-5 pd30 cells/ml, 2×10^3 HCT116 $p53^{+/+}$, 2×10^3 ARPE19 cells/ml, 3×10^3 WI-38 cells/ml. The cells were used until reaching 70-80% confluency in the stock flasks. Complete cell media containing DMEM, supplemented with 10% foetal calf serum and L-glutamine (2 mM), was used to prepare the desired cell concentration and reference wells. Plates containing cells were incubated for 24 h at 37°C (48 h for WI-38) in an atmosphere of 5% CO_2, prior to drug exposure. Cell media (200 µl) was added to the reference cells and differing concentrations (0 to 50 µM) of drug solution (200 µl) were added to the remaining wells. (All complexes were directly dissolved in cell media except for the [Fe_2**L**$^{\mathbf{3n}}_3$], which were dissolved in DMSO to a final DMSO concentration <0.1% due to low solubility in water.) The plates were incubated for a further 72 or 96 h at 37°C in an atmosphere of 5% CO_2. Due to these compounds being purple in colour, media is removed and replaced with fresh media prior to the assay. 3- (4,5-Dimethylthiazol-1-yl)-2,5-diphenyltetrazolium bromide (MTT) solution (0.5 mg/ml, 20µl per well) was added to each well and incubated for a further 4 h at 37°C in an atmosphere of 5% CO_2. Upon completion all solutions were removed from the wells and dimethyl sulfoxide (150 µl, {75 µl for WI-38}) was added to each well to dissolve the purple formazan crystals. A Thermo Scientific Multiskan EX microplate photometer was used to measure the absorbance at 540 nm. Lanes containing 100% cell media and untreated cells were used as a blank and 100% cell survival respectively. Cell survival was determined as the absorbance of treated cells minus the blank cell media, divided by the absorbance of the untreated control; this value was expressed as a percentage. The IC_{50} values were determined from a plot of percentage cell survival against drug concentration (µM). All assays were conducted in triplicate and the mean IC_{50} ± standard deviation was determined.

Supplementary Table 1 Antiproliferative activity and Selectivity index of aromatic clicked triplex $[Fe_2L^n{}_3]Cl_4$ (n = 3a-3e), unclicked alkyne triplex $[Fe_2L^2{}_3]Cl_4$ and the parent triplex $[Fe_2L^1{}_3]Cl_4$ in HCT116 $p^{53+/+}$ and ARPE-19 cell lines

		mean IC_{50} (µM)		Selectivity Index
		**HCT116 $p^{53+/+}$**	**ARPE-19**	
$[Fe_2\mathbf{L}^{3a}{}_3]Cl_4$	Λ	0.91 ± 0.28	8.82 ± 1.04	10
	Δ	2.16 ± 0.97	65.58 ± 6.82	30
$[Fe_2\mathbf{L}^{3b}{}_3]Cl_4$	Λ	1.00 ± 0.19	6.88 ± 1.20	7
	Δ	1.94 ± 0.84	25.37 ± 1.94	13
$[Fe_2\mathbf{L}^{3c}{}_3]Cl_4$	Λ	0.73 ± 0.30	3.08 ± 0.15	4
	Δ	2.25 ± 0.96	76.14 ± 3.66	34
$[Fe_2\mathbf{L}^{3d}{}_3]Cl_4$	Λ	2.13 ± 2.10	2.94 ± 0.31	1.4
	Δ	3.13 ± 0.47	59.60 ± 5.60	19
$[Fe_2\mathbf{L}^{3e}{}_3]Cl_4$	Λ	1.63 ± 1.13	8.75 ± 0.63	5
	Δ	10.15 ± 2.12	72.23 ± 10.52	7
$[Fe_2\mathbf{L}^{2}{}_3]Cl_4$	Λ	5.02 ± 0.21	73.81 ± 13.05	15
	Δ	2.87 ± 0.91	100.44 ± 4.67	35
$[Fe_2\mathbf{L}^{1}{}_3]Cl_4$	Λ	1.41± 0.39	10.0 ± 1.77	7
	Δ	21.4 ± 1.4	31.16 ± 11.63	1.4

8.2 Cell Cycle assay

ARPE-19 or HCT116 $p53^{+/+}$ cells were seeded (5×10^5 cells per well) in 6-well plates and incubated for 24 h at 310 K. Cells were then treated with the investigated compounds at 2 × IC_{50} concentration and incubated for a further 24 h. After the treatment period, cells were detached using 0.25% trypsin (Sigma), washed twice in PBS (Sigma), and then re-suspended in ethanol for 1 h on ice. A staining solution of 80 µg/mL RNAse (Sigma) and 50 µg/mL propidium iodide (Thermo Fisher) was prepared in PBS, and added to the cells (30 min, 298 K). Excess dye was removed, and cells were re-suspended in PBS before analysed under flow cytometry. Flow cytometry data was analysed on a Beckton Dickinson LSRII, using BD FACSDIVA V8.0.1 software. Samples were analysed as instrumental triplicates and the cell counter was placed to 1×10^5 counts for a single stain experiment. Samples and controls were analysed by flow cytometry then data processing on Flowjo V10. Welch's two-tailed t-test was carried out for statistical analysis.

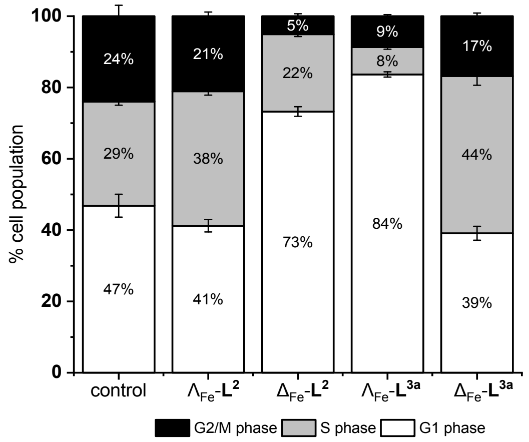

Supplementary Figure 24 (a) Cell cycle analysis by flow cytometry of propidium iodide-stained cells and quantification of the percentage of the cell population in different stages of the cell cycle for untreated ARPE19 cells, and following incubation with the metallohelices for 24 h (at a concentration of twice the 96 h ARPE19 IC_{50}).

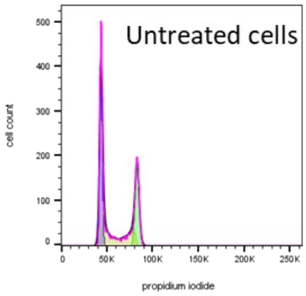

RMSD : 2.01
%G1 : 30.3
%S : 20.0
%G2 : 19.4
G1 Mean : 43183
G2 Mean : 82669
G1 CV : 4.14
G2 CV : 3.58
% less G1 : -0.25
% greater G2 : -0.78

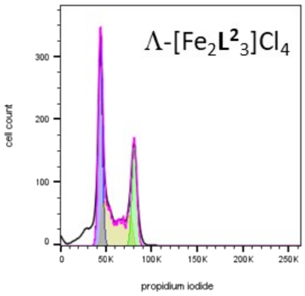

RMSD : 2.95
%G1 : 20.2
%S : 18.4
%G2 : 11.2
G1 Mean : 43378
G2 Mean : 80648
G1 CV : 7.41
G2 CV : 4.59
% less G1 : 7.04
% greater G2 : 1.31

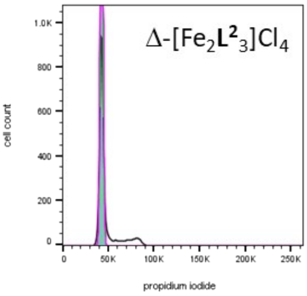

RMSD : 42.7
%G1 : 61.0
%S : -16.0
%G2 : 61.0
G1 Mean : 42451
G2 Mean : 42451
G1 CV : 4.84
G2 CV : 4.84
% less G1 : -23.1
% greater G2 : -21.3

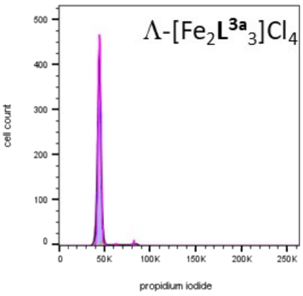

RMSD : 1.65
%G1 : 19.7
%S : 1.30
%G2 : 0.072
G1 Mean : 44053
G2 Mean : 81641
G1 CV : 4.84
G2 CV : 0.34
% less G1 : 0.38
% greater G2 : 0.29

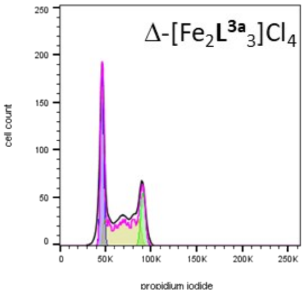

RMSD : 1.55
%G1 : 10.1
%S : 13.3
%G2 : 4.42
G1 Mean : 45960
G2 Mean : 90651
G1 CV : 5.32
G2 CV : 3.87
% less G1 : 1.35
% greater G2 : 0.35

Supplementary Figure 25 Raw data for cell cycle analysis by flow cytometry of propidium iodide-stained cells and quantification of the percentage of the cell population in different stages of the cell cycle for untreated ARPE19 cells, and following incubation with the metallohelices for 24 h (at a concentration of twice the 96 h ARPE19 IC_{50}).

8.3 Apoptosis assay (Annexin V assay)

Cells were prepared and treated as described for cell cycle assay. After detaching and washing, a staining solution of 1% v/v FITC Annexin V (Abcam) and 2% v/v propidium iodide was added to the cells (30 min, 298 K) before analysed under flow cytometry.

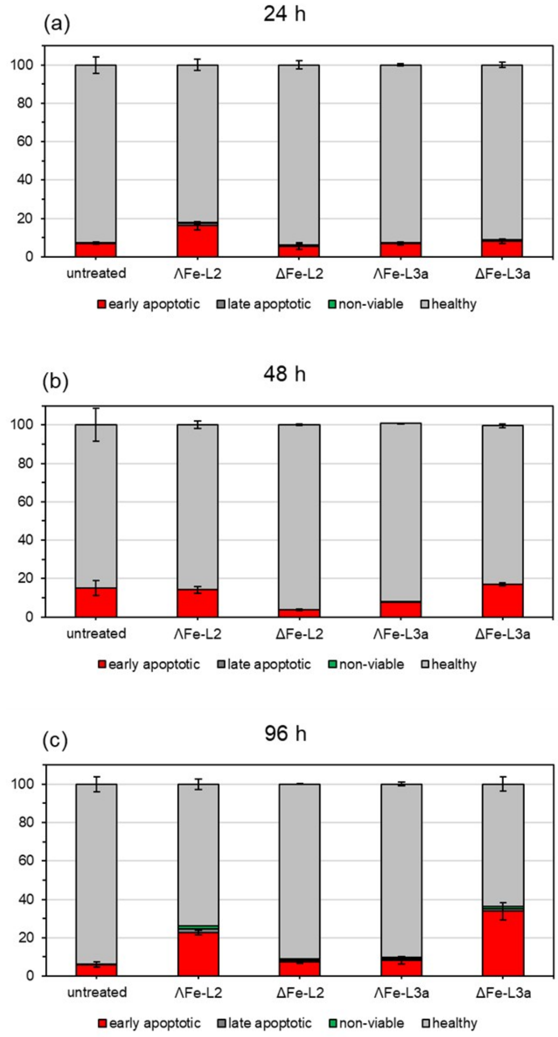

Supplementary Figure 26 Induction of apoptosis in HCT116 p53$^{+/+}$ cells following (a) 24 h (b) 48 h and (c) 96 h incubation with the indicated metallohelices, measured using an Annexin V assay.

One quantifiable marker of early apoptosis is an increase in membrane phosphatidylserine (PS), but this was not observed for HCT116 p53$^{+/+}$ cells that had been incubated with enantiomers of $[Fe_2\mathbf{L}^2]Cl_4$ and $[Fe_2\mathbf{L}^{3a}{}_3]Cl_4$ at 2 × IC_{50} for 24 and 48 h. This suggests that other mechanisms are contributing to their activity towards the HCT116 cancer cells. After 96 h at this concentration, Λ-$[Fe_2\mathbf{L}^2{}_3]Cl_4$ and Δ-$[Fe_2\mathbf{L}^{3a}{}_3]Cl_4$ induced modest levels of apoptosis.

8.4 Real-time cell growth monitoring

Real-time Cell Analyzer (RTCA) (xCELLigence RTCA SP Instrument, ROCHE) was employed for growth monitoring of cells in the absence and the presence of tested compounds. After the background of E-plates reading (100 μL medium), the cells were added ($5x10^3$ cells/well; 50 μL) and grown for 22-28 h. Tested compounds at varying concentrations were then added in 50 μL of media. The impedance was monitored for additional 80 h. An arbitrary unit CI (cell index) is a quantitative measure reflecting the status of the cells (number of attached cells and cell status such as morphology) in an electrode-containing well. Normalized CI at a given time point is given by dividing CI at the time point by CI at a reference time point.

8.5 Rubidium based assay for detection of Na^+/K^+ ATPase activity

The cells were seeded on 6-well plates (5×10^5 cells/dish) and incubated overnight. The cells were treated with the investigated compounds for 6 h at 10 μM concentration. Following the treatment, the medium was removed, and the cells were washed twice with PBS. Solution A (KCl) or solution B (RbCl) was added to the cells (solution A: 15 mM Hepes, 140 mM NaCl, 5.4 mM KCl, 1 mM $MgCl_2$, 5mM Glucose, 0.8 mM NaH_2PO_4 and 2 mM $CaCl_2$, pH 7.4 and solution B: 15 mM Hepes, 140 mM NaCl, 5.4 mM RbCl, 1 mM $MgCl_2$, 5mM Glucose, 0.8 mM NaH_2PO_4 and 2 mM $CaCl_2$, pH 7.4). Solution A contained KCl and no RbCl and was added to untreated control cells. Solution B contained no RbCl and no KCl and was added to untreated or treated cells. The cells were incubated with solutions A or B for 3 h. The cells were then harvested, washed trice with PBS, counted and pelleted. Rubidium content was determined with MS-ICP.

9. Antimetastatic study

9.1 Resistance to detachment assay

Following procedure was used to measure the ability of cells to resist detachment by trypsin. HCT116 or A2780 cells were seeded at a density of 5×10^3 cells/well in 200 μL complete medium in a 96-well plate. After 2 days at 37 °C in 5% CO_2 complete medium was replaced with serum-starved medium containing 0.1% w/v BSA (bovine serum albumin). After another 24 h, the medium was removed, and the cells were treated for 3 h with 10 μM and 20 μM metallohelices in the serum-starved medium. Following the incubation the medium was removed again, the cells were washed with PBS, and 30 μL of (0.005% w/v) trypsin solution was added to each well, and the plates were incubated at 37 °C for 12 min. At the end of the incubation, the trypsin solution was removed, and wells were washed with PBS. Cells that were

still adherent to the plates were fixed with 200 µL of 10% (w/v) cold TCA (trichloracetic acid) at 4 °C for 1 h. The adherent cells were detected by the sulforhodamine B (SRB) assay. Absorbance values were plotted against compound concentration.

9.2 Re-adhesion assay

The ability of HeLa and HCT116 cells treated with metallohelices to re-adhere after detachment was studied using the re-adhesion assay. The cells were seeded at a density of 2×10^5 cells/well (6-well plate) in 3 mL of the complete medium. After 48 h the medium was replaced with a serum-starved medium (0.1% w/v BSA), and the cells were grown for another 24 h. Then the medium was removed, and the cells were treated with metallohelices (10 µM) in the same medium for 3 h. The medium was removed, and the cells were washed twice with PBS. The cells were trypsinized (0.05% trypsin, 5 min), collected by centrifugation, re-suspended in serum-starved medium supplemented with 0.1% w/v BSA and kept for 30 min at room temperature for surface receptor reconstitution. The cells were seeded in octuplicates on 96-well plate at a density of 2×10^4 cells in 0.1 mL/well. Cells were incubated (37 °C, 5% CO_2) to adhere for 30 min. Following the incubation, the medium was removed, and the wells were twice gently washed twice with PBS. The adhered cells were determined by the sulforhodamine B (SRB) test.

9.3 Cell invasion test

For the invasion test, HCT116 $p53^{+/+}$ were seeded at a density of 3×10^5 cells in T25 flasks. Cells were incubated for 72 h at 37 °C in a 5% CO_2 humidified atmosphere in the complete culture medium (10% FBS), followed by 24 h incubation in a serum-free medium supplemented with 0.1% w/v. The cells were subsequently treated with the compounds at the concentrations corresponding to $2\times IC_{50}$ (72 h) for 2 h in PBS at 37 °C in a 5% CO_2 humidified atmosphere. The cells were washed with PBS, collected using trypsin-EDTA (0.25% in PBS), counted and seeded at the density of 5×10^4 cells per one insert (Corning; 8 µm pore size) coated with Matrigel. For the negative control, the bottom parts of the plate were filled with serum-free medium supplemented with 0.1% w/v BSA. The positive control was performed in the complete medium containing 10% FBS. Cells were left to invade for 96 h at 37 °C in a 5% CO_2 humidified atmosphere. Invaded cells were stained with crystal violet, and the absorbance was measured at 590 nm with an Absorbance Reader (SUNRISE TECAN, SCHOELLER). Values

of absorbance were normalized to an appropriate absorbance of control and expressed as normalized invasion (% of control).

9.4 Wound healing assay

HCT116 p53$^{+/+}$ cells were seeded on a 12-well plate at a density of 5×10^4 cells/well in 1 mL complete medium (10% FBS/DMEM/Gentamycin) and incubated (37 °C, 5% CO_2). Following removal of the medium, the bottom of the well was scratched with a 10 µl pipet tip. The wells were washed with PBS twice to remove any detached cells or cell debris. 550-650 µm gaps were obtained. Complete medium with or without metallohelices was added, and the plates were kept in the incubator at 37 °C for 24 h. In a different protocol, the complete medium was replaced for starving medium (0.1% BSA, gentamycin) 16 h before the treatment and the cells were treated and incubated for the rest of the assay in the same starving medium. Images were taken at several time intervals after scratching with a system consisting of Olympus CKX41 Inverted Microscope, Canon EOS 1200D camera, and QuickPHOTO MICRO 3.1 software. Automated analysis of wound healing was performed using TScratch software (MATLAB).

Cell Viability was measured under these assay conditions (i.e. following 24 h drug dose, and 72 h time lapse following the dose).

Supplementary Table 2. Quantification cell viability of HCT116 p53$^{+/+}$ cells untreated or treated with the investigated compounds under the wound healing assay conditions. Viability determined using the NC-3000 viability and total cell count analysis with Via-1 cassettes (pre-impregnated cassettes containing DAPI (stains non-viable cells) and acridine orange (stains whole population).

	Water control	DMSO control	Δ-[Fe_2**L**$^1{}_3$]Cl_4	Δ-[Fe_2**L**$^2{}_3$]Cl_4	Δ-[Fe_2**L**$^{3a}{}_3$]Cl_4
**Average total cell count (*n*=3)**	1.26x10^6	1.56 x10^6	1.4 x10^6	1.55 x10^6	1.2 x10^6
**Average total % cell count (SD) compared to control**	100	100	114.25 (35.7)	122.96 (31.1)	77.35 (41.6)
**Average % viability (SD) (% living cells not total)**	90.33 (2.20)	90.87 (3.57)	88.93 (4.38)	89.4 (4.85)	84.93 (8.76)

These viability tests confirm that non-toxic doses are used (cell viability = ca 90% in all cases), and thus inhibition of gap closure indicates migration inhibition.

10. Anti-cancer stem cell study

10.1 Inhibition of HCT116 colonsphere formation

HCT116 p53$^{+/+}$ (adherent) cells were treated with Δ-[Fe_2**L**$^1{}_3$]Cl_4, Δ-[Fe_2**L**$^{3a}{}_3$]Cl_4 and salinomycin (at their IC_{30} values) for 72 h. Cells were then washed with PBS, harvested by StemProAccutase (Gibco) and plated in ultralow-attachment 96w culture plates (Corning) (300cells per well). Cells were cultured in DMEM/F12 supplemented with B27 (-vitA, Gibco, ThermoFisher, 12587010), 4 mgmL^{-1} BSA (Sigma), 20 ngmL^{-1} EGF (Sigma, E5036) and 10 ngmL^{-1} FGF2 (Sigma, F0291) for 6 days without disturbing the plates and without replenishing the medium. The number and size of spheres were determined using an inverted microscope.

Supplementary Table 3. Quantification of colonospheres formation in HCT116 p53$^{+/+}$ cells untreated or treated with the investigated compounds at their respective IC_{30} values for 72 h.[a]

HCT116 p53$^{+/+}$	Spheres/1000cells	Diameter (μm)
**Control**	224 ± 14	96 ± 20
**Salinomycin**	142 ± 14	76 ± 17
Δ-[Fe_2**L**$^1{}_3$]Cl_4	134 ± 15	86 ± 18
Δ-[Fe_2**L**$^{3a}{}_3$]Cl_4	94 ± 22	82 ± 16

[a]The results are expressed as mean ± SD of three independent experiments.

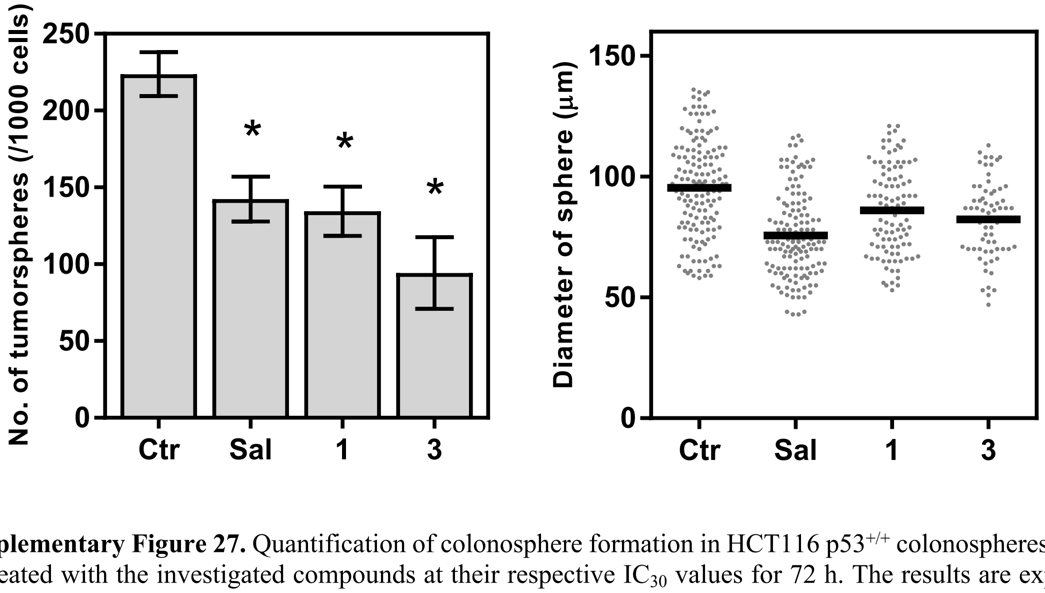

Supplementary Figure 27. Quantification of colonosphere formation in HCT116 p53$^{+/+}$ colonospheres untreated or treated with the investigated compounds at their respective IC_{30} values for 72 h. The results are expressed as mean ± SD of three independent experiments. The symbol (*) denotes significant difference ($p < 0.0001$) from the untreated control.

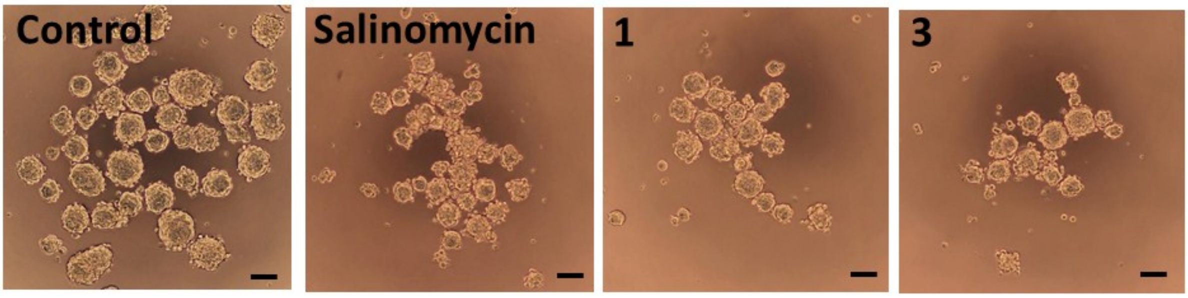

Supplementary Figure 28. Representative images of the HCT116 p53$^{+/+}$ colonospheres untreated or treated with the investigated compounds at their respective IC_{30} values for 72 h. Scale bars: 100 μm.

The results (Table 3 and Figure S26 and S27 show that Δ-[Fe_2**L**^{3a}$_3$]Cl_4 inhibits HCT116 p53$^{+/+}$ colonosphere formation similarly or more effectively than Δ-[Fe_2**L**^{1}$_3$]Cl_4 and salinomycin, a well-established CSC-potent agent. This observation suggests that Δ-[Fe_2**L**^{3a}$_3$]Cl_4 might be an effective agent exhibiting anti-CSC potency similar or even better than conventional salinomycin.

10.2 Separation of HCT116.CD133$^+$ and HCT116.CDD133$^-$ cells by magnetically activated cell sorting (MACS)

Positive selection with a single marker of CD133/1 (AC133) was used to enrich for CSCs. The cells were harvested by non-enzymatic Cell Stripper (Corning), prepared into single cell suspension (30 μm strainer) and counted. Approx. 2×10^7 cells were obtained for cell sorting. For a selection of CD133 single positive cells, the cells were stained with the APC-conjugated CD133 monoclonal antibody (Miltenyi Biotec, CD133/1 (AC133), 130-090-826) and incubated for 20 min at 4-8 °C. The cells were washed with buffer (PBS, 0.5% BSA, pH 7.2; prepared by diluting MACS BSA Stock Solution (130-091-376) 1:20 with auto MACS Rinsing Solution (130-091-222)) and incubated with Anti-APC Micro Bead (Miltenyi Biotec, 130-090-855) for 20 min at 4-8 °C and then washed with buffer and passed through pre-separation filter (Miltenyi Biotec, 30 μm, 130-041-407). The sorting MS column was fixed on a MACS stand (Miltenyi Biotec) and equilibrated by applying 500 μL of buffer. The cell suspension was applied on the MS column and the flow-through fraction was collected as HCT116.CD133$^-$ cells. After removal of the column from the magnetic field, the magnetically retained cells were eluted and collected as HCT116.CD133$^+$-enriched cells. After separation, the cell suspension went through a MACS MS column, which was placed in mini MACS, CD133$^+$ cells were enriched. After separation, HCT116.CD133$^+$ were maintained in serum-free medium (DMEM/F12, 0.4% BSA, B27, 10 ngmL^{-1} FGF, 20 ngmL^{-1} EGF) in ultralow-attachment culture flasks (CORNING) or used immediately for experiment.

10.3 Flow cytometric analysis of surface marker expression levels

Adherent HCT116 p53$^{+/+}$ cells and spheroid HCT116 p53$^{+/+}$ and HCT116.CD133^{+} cells were washed with Dulbecco's phosphate-buffered saline (DPBS) and dissociated by Cell Stripper (Corning) to single cell suspensions. Single cells were washed with Buffer (PBS, 0.5% BSA, pH 7.2, prepared by diluting MACS BSA Stock Solution (130-091-376) 1:20 with auto MACS Rinsing Solution (130-091-222)) and stained with the APC-conjugated CD133 monoclonal antibody (Miltenyi Biotec, CD133/1 (AC133), 130-090-826). FcR Blocking Reagent (130-059-901) was added and incubated for 20 min at 4-8 °C. After staining, the cells were washed three-times in Buffer and analyzed on BD FACSVerse flow cytometer (San Jose, CA, USA); the data were analyzed using BD FACSSuite software.

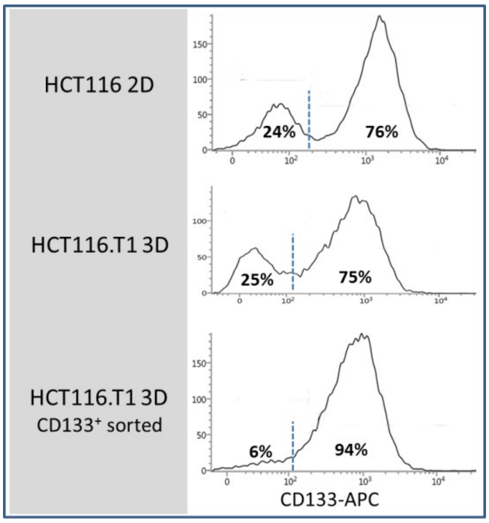

Supplementary Figure 29. Representative histograms displaying the red fluorescence emitted by anti-CD133-APC antibody stained HCT116 p53$^{+/+}$ cells: HCT116 2D – adherent, HCT116.T1 3D – colonospheres, HCT116.T1 3D CD133^{+} sorted – colonospheres from HCT116.CD133^{+} sorted cells, T1 denotes the first passage of the colonospheres = primary tumorspheres.

10.4 Inhibition of HCT116.CD133^{+} and/or HCT116.CD133^{-} colonsphere formation

Sorted HCT116.CD133^{+} cells were plated in 96w ULA culture plates (Corning) (300 cells per well) and immediately treated with Δ-[Fe$_2$**L**^{1}$_3$]Cl$_4$, Δ-[Fe$_2$**L**^{3a}$_3$]Cl$_4$ or salinomycin (at their IC$_{30}$ values) for 6 days. Cells were cultured in DMEM/F12 supplemented with B27 (-vitA, Gibco, ThermoFisher, 12587010), 4 mgmL^{-1} BSA (Sigma), 20 ngmL^{-1} EGF (Sigma, E5036) and 10 ng/mL FGF2 (Sigma, F0291) for 6 days without disturbing the plates and without replenishing the medium. The number and size of spheres were determined using an inverted microscope.

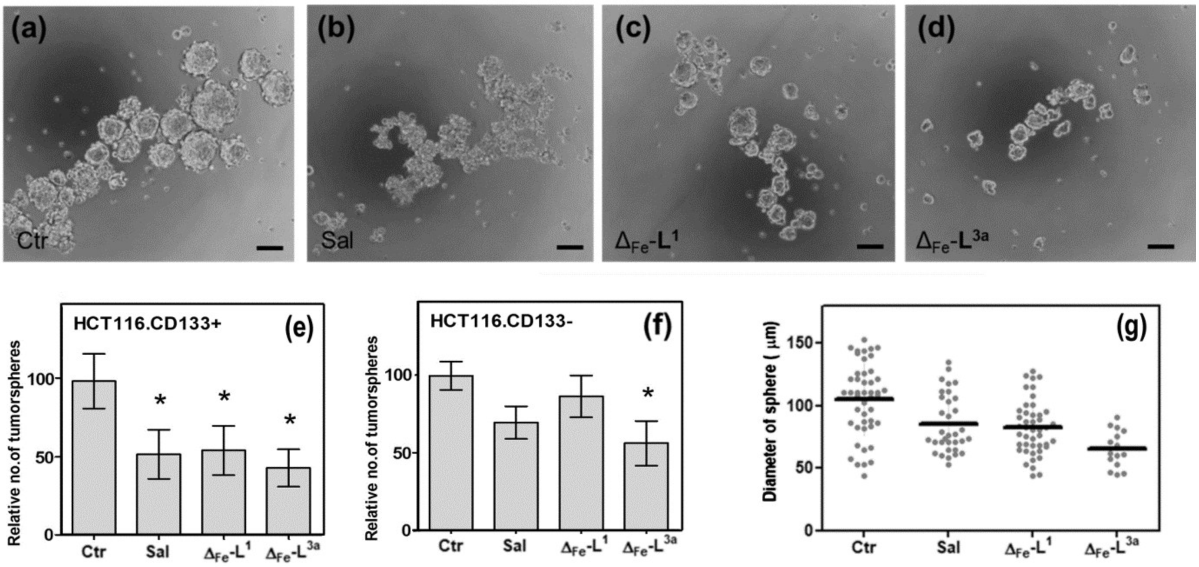

Supplementary Figure 30. Growth inhibitory effects in HCT116.CD133$^-$ and HCT116.CD133$^+$ cancer cells. Representative microscopy images of the HCT116.CD133$^-$ colonospheres in the absence (a) and presence of salinomycin (b), Δ-[Fe_2**L**$^1_3$]Cl_4 (c), and Δ-[Fe_2**L**$^{3a}_3$]Cl_4 (d), treated at their respective IC_{30} values for 6 days (scale bar: 100 µm). Quantification of colonosphere formation from HCT116.CD133$^+$ (e) and HCT116.CD133$^-$ (f,g) under the same conditions. Data represent the mean value and SD from three independent experiments. $p < 0.01$, versus control.

10.5 Cytotoxicity in HCT116.CD133$^+$ cells, SRB assay

Sorted HCT116.CD133$^+$ cells were plated in 96w culture plates (TPP) (3000 cells per well) and cultured for 3 days to allow cells to recover after sorting and to grow to the desired density. Cells were then treated with different concentrations of the investigated compounds, salinomycin, Δ-[Fe_2**L**$^1_3$]Cl_4 or Δ-[Fe_2**L**$^{3a}_3$]Cl_4 for 72 h. After the treatment period, the cells were washed twice with PBS and fixed with 10% trichloroacetic acid (TCA). After 1 h, the fixed cells were washed with MiliQ water and incubated with 0.4% SRB for 30 min. Wells were washed three times with 1% acetic acid, and SRB stained cellular proteins were dissolved with Tris base (pH 10.5). The absorbance at 570 nm was measured using a SUNRISE Tecan absorbance reader (Schoeller). The IC_{50} values were calculated from curves constructed by plotting cell survival (%) versus drug concentration (µM). The reading values were converted to the percentage of control (% cell survival). Cytotoxic effects were expressed as IC_{50}.

Supplementary Table 4. IC_{50} values of the investigated compounds in CSC enriched HCT116.CD133$^+$ cells determined with SRB assay.[a]

IC_{50} (µM)	HCT116.CD133$^+$	HCT116p53$^{+/+}$
**Salinomycin**	1.12 ± 0.23	1.48 ± 0.21
Δ-[Fe_2**L**$^1_3$]Cl_4	2.04 ± 0.39	3.28 ± 0.30
Δ-[Fe_2**L**$^{3a}_3$]Cl_4	1.21 ± 0.25	2.11 ± 0.41

[a]The cells were treated for 72 h. The results are expressed as mean ± SD of three independent experiments.

The cytotoxicity of Δ-$[Fe_2\mathbf{L}^{3a}{}_3]Cl_4$ was tested in isolated stem $CD133^+$ cells from the HCT116 $p53^{+/+}$ cell line, and an IC_{50} of 1.21 ± 0.25 μM was measured in HCT116.$CD133^+$, using the SRB assay following a 72 h exposure. This is *ca* 40% lower than the IC_{50} measured for HCT116$p53^{+/+}$ cells under the same conditions, similar to that of salinomycin.

10.6 Clonogenic assay / Colony formation assay

Sorted HCT116.$CD133^+$ cells were seeded in 6-well ULA plates (Corning) (10000 cells/well) and cultured for 4 days to allow cells to form pre-spheroids. Cells were then treated with different concentrations of the investigated compounds for 48 h. After the treatment the HCT116.$CD133^+$ spheroids were dissociated into single cell suspensions with StemProAccutase (Gibco) and seeded on normal 6-well plates (TPP) at the density of 3000 cells/well. After 8 days the staining of formed colonies was done with a methylene blue solution (1% in water/methanol 1:1) added for 30 min. After washing out excess dye, the plates were allowed to air dry before manually counting colonies containing 50 or more cells. The surviving fraction (SF) is the number of colonies that form after treatment relative to the number of cells seeded. SF is calculated as:

$$SF = \frac{number\ of\ colonies\ formed\ after\ treatment}{number\ of\ cells\ seeded\ x\ PE} \text{ x } 100\%$$

where PE is plating efficiency defined as the ratio of the number of colonies to the number of untreated cells seeded. For these HCT116.$CD133^+$ cells PE ~ approx. 0.12 (or 12%).

11. Supplementary References

1. P. Das, A. Ghosh, M. K. Kesharwani, V. Ramu, B. Ganguly and A. Das, *European Journal of Inorganic Chemistry*, 2011, **2011**, 3050-3058.
2. C. Dallaire, I. Kolber and M. Gingras, *Organic syntheses*, 2002, **78**, 42-50.
3. M. Seredyuk, A. Gaspar, V. Ksenofontov, Y. Galyametdinov, J. Kusz and P. Gütlich, *Journal of the American Chemical Society*, 2008, **130**, 1431-1439.
4. Y. Hsiao and L. S. Hegedus, *The Journal of Organic Chemistry*, 1997, **62**, 3586-3591.
5. S. E. Howson, G. J. Clarkson, A. D. Faulkner, R. A. Kaner, M. J. Whitmore and P. Scott, *Dalton Transactions*, 2013, **42**, 14967-14981.
6. A. D. Faulkner, R. A. Kaner, Q. M. Abdallah, G. Clarkson, D. J. Fox, P. Gurnani, S. E. Howson, R. M. Phillips, D. I. Roper, D. H. Simpson and P. Scott, *Nature Chemistry*, 2014, **6**, 797-803.
7. W. G. Kim, M. E. Kang, J. B. Lee, M. H. Jeon, S. Lee, J. Lee, B. Choi, P. M. Cal, S. Kang and J.-M. Kee, *Journal of the American Chemical Society*, 2017, **139**, 12121-12124.
8. Y.-W. He, C.-Z. Dong, J.-Y. Zhao, L.-L. Ma, Y.-H. Li and H. A. Aisa, *European journal of medicinal chemistry*, 2014, **76**, 245-255.

9. S. Lal, H. S. Rzepa and S. Diez-Gonzalez, *ACS Catalysis*, 2014, **4**, 2274-2287.
10. Y. Liu, Q. Xiao, Y. Liu, Z. Li, Y. Qiu, G. B. Zhou, Z. J. Yao and S. Jiang, *Eur J Med Chem*, 2014, **78**, 248-258.
11. M. I. Montanez, Y. Hed, S. Utsel, J. Ropponen, E. Malmstrom, L. Wagberg, A. Hult and M. Malkoch, *Biomacromolecules*, 2011, **12**, 2114-2125.
